# Thiosugar-functionalized gold(I)-NHC complexes as selective anticancer agents for potential targeted therapy

**DOI:** 10.3389/fchem.2026.1724206

**Published:** 2026-02-26

**Authors:** Ester Giorgi, Tarita Biver, Michele Mannelli, Tania Gamberi, Matteo Becatti, Giuseppina Sabatino, Elisa Peroni, Olivier Monasson, Damiano Cirri, Chiara Gabbiani, Alessandro Pratesi

**Affiliations:** 1 Department of Chemistry and Industrial Chemistry, University of Pisa, Pisa, Italy; 2 Department of Experimental and Clinical Sciences “Mario Serio”, University of Florence, Florence, Italy; 3 National Research Council (CNR) Crystallography Institute, Catania, Italy; 4 BioCIS UMR 8076, CNRS, CY Cergy Paris Université, Cergy-Pontoise, France; 5 BioCIS UMR 8076, CNRS, Université Paris-Saclay, Orsay, France

**Keywords:** bioconjugates, gold(I)-NHC complexes, metal-based drugs, ovarian cancer, targeting strategies

## Abstract

Four novel gold(I) N-heterocyclic carbene (NHC) complexes were synthesized and characterized; they are tuned in terms of the aromatic extension of the NHC scaffold and two of them contain a thiosugar residue to enhance their cellular uptake. To verify their potential interaction with human serum albumin (HSA), ESI-MS interaction analysis and fluorescence titrations were performed. Biological studies were carried out to evaluate their possible cytotoxic effect on three ovarian cancer cell lines, i.e., A2780 (both sensitive and cisplatin-resistant), and SKOV-3. Confocal microscopy and fluorescence-activated cell sorting tests were also carried out for the four complexes. Thiosugar conjugation proved to be an effective strategy to enhance potency and selectivity, resulting in a considerable improvement compared to the corresponding complexes lacking the thiosugar moiety. Furthermore, six bioconjugates containing targeting peptides were synthesized; in most cases, no significant improvement in either cytotoxic activity or selectivity was observed, except for the LHRH peptide conjugates, which showed a slight enhancement in both cytotoxicity and selectivity compared to the unconjugated complexes.

## 1 Introduction

Metal-based complexes have attracted considerable interest in the last decades in the search for new anti-cancer drugs, particularly due to their unique characteristics of geometric coordination, tunable redox properties, and ability to interfere with various key pathways exploited by tumor cells. Furthermore, metallorganic complexes, depending on the metal center and ligands, can exhibit diverse mechanisms of action, thereby expanding their range of applicability to various types of tumors (Gasser et al., 2011; Zhang and Sadler, 2017). However, intrinsic or acquired resistance of the cancer cells and the poor selectivity of the metal-based chemotherapy lead to the heaviest side effects in patients (Galluzzi et al., 2014; Santos et al., 2020; Tredan et al., 2007). For this reason, researchers are developing new generations of drugs exploiting different strategies in order to overcome the mentioned drawbacks. Among the various approaches used, some interesting ones are the design of metallodrugs acting as immunomodulatory agents, inducers of immunogenic cell death (ICD), and modulators of stress response in cancer cells or ferroptosis (Li et al., 2024; Lu et al., 2025; Wang et al., 2025).

Another appealing strategy involves the conjugation to targeting moieties to selectively reach the tumor site (Giorgi et al., 2022; Stine et al., 2022; Zhong et al., 2021). This approach aims to direct metallodrugs toward cancer-specific biomarkers or other biological targets that are overexpressed in cancer cells (Brunner and Barton, 2006; Erxleben, 2018; Giorgi et al., 2022; Wisnovsky et al., 2013). The drug is then accumulated preferentially or selectively in cancer cells, potentially lowering the undesired side effects typical of the classic platinum-based chemotherapy. In the last few decades, a significant interest in gold-based *N*-heterocyclic carbenes (NHCs) has risen, particularly for their promising anticancer activity (Brescia and Ronconi, 2023; Moreno-Alcántar et al., 2023; Nardon et al., 2014). The ability of gold(I) *N*-heterocyclic carbenes to interact with biomolecular targets such as DNA and human serum albumin (HSA) has been reported in several papers (Bertrand et al., 2014b; Guarra et al., 2020; Visbal et al., 2016). The study of these types of interactions, which include binding through intercalation or electrostatic forces and HSA interaction influencing biodistribution, is crucial to understanding their pharmacological behavior and cytotoxic activity.

**FIGURE 1:**
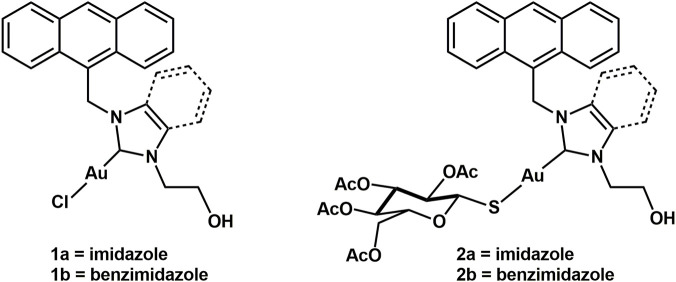
Chemical structures of complexes **1a-b** and **2a-b**.

The latter property seems mainly due to gold-based complexes’ ability to inhibit the mitochondrial thioredoxin reductases (TrxR2), even though it has also been discovered that other mechanisms of action seem to be relevant (Berners-Price and Filipovska, 2011; Bindoli et al., 2009). TrxR2 is a selenoenzyme responsible for maintaining redox homeostasis in normal cells in physiological conditions, and it is often overexpressed in tumors (Pratesi et al., 2014; Scalcon et al., 2018; Urig and Becker, 2006). TrxR2 inhibition in cancer cells has proven to play a crucial role in apoptosis evasion (Zhang et al., 2017). The lack of the TrxR2 enzymatic activity increases the mitochondrial concentration of reactive oxygen species (ROS), triggering significant swelling and a decrease of mitochondrial membrane potential (MMP) (Jia et al., 2019). These substantial alterations in redox signaling events consequently induce programmed cell death (Zhang et al., 2017). Within this framework, we present the synthesis, characterization and investigation of the biological activity of four new cytotoxic gold(I)-NHC complexes with potential anticancer properties against human ovarian cancer cells (Figure 1). For this purpose, two new Au(I)-NHC complexes were synthesized (**1a-b**, Figure 1) and then functionalized to 3,4,5,6-tetra-*O*-acetyl-1-thio-β-D-glucopyranose, obtaining a final panel of four molecules. Moreover, the complexes are endowed with a fluorescent anthracenyl tag. Several studies have reported the synthesis and characterization of anticancer Au(I)–NHC complexes conjugated to different fluorescent probes, such as acridine, coumarin, naphthalimide, and Ru (bipy)_3_, thus demonstrating the versatility of gold complexes for imaging applications (Bertrand et al., 2014a; Boselli et al., 2015; Meyer et al., 2014; Visbal et al., 2016). These studies reported in the literature highlight how the properties of the fluorophore can be crucial for addressing specific biological or photophysical needs. In the context of this project, the anthracenyl moiety was selected as the fluorescent probe due to its well-established photophysical properties, its chemical stability, and its straightforward functionalization and versatility, being a suitable model fluorophore for proof-of-concept studies. Moreover, anthracene has already been used successfully in similar compounds (Citta et al., 2013; Fabbrini et al., 2019; González et al., 2019; Guarra et al., 2020). Indeed, as a planar and hydrophobic moiety acting as a DNA intercalator, anthracene is able to modulate the interaction of metal complexes with both DNA and human serum albumin, influencing their cellular uptake and distribution (Binacchi et al., 2020; Visbal et al., 2016; Yan et al., 2010).

The approach consists of a passive-targeting strategy exploiting the Warburg effect; indeed, the conjugation of the two new gold(I) complexes with a biomimetic glucose substrate can make the molecule a semi-targeted metabolite, increasing the metal-complex uptake in tumor cells by exploiting the overexpressed GLUT-mediated transporters. This type of conjugation, where the glucose-mimicking moiety acts as a Trojan horse in the cancer cell, can lead to a higher drug accumulation in the diseased cells than in healthy cells (Chen et al., 2016; Dada et al., 2018; Liberti and Locasale, 2016; Vander Heiden et al., 2009). The selected sugar for this purpose was 3,4,5,6-tetra-*O*-acetyl-1-thio-β-D-glucopyranose. The use of the latter thiosugar in gold(I)-NHC carbenes has already been reported in the literature (Dada et al., 2018; Safir Filho et al., 2021). In addition to targeting metabolite-based approaches, conjugation to targeting peptides represents a versatile strategy to enhance the accumulation of metal-based drugs in tumors. The peptides can be designed to recognize transmembrane or intracellular receptors that are overexpressed in cancer cells, or to penetrate the cell membrane and deliver the drug to specific organelles such as the nucleus, mitochondria, or lysosomes, potentially increasing both selectivity and therapeutic efficacy of the drug (Giorgi et al., 2022).

## 2 Results and discussion

### 2.1 Synthesis and characterization

**SCHEME 1:**
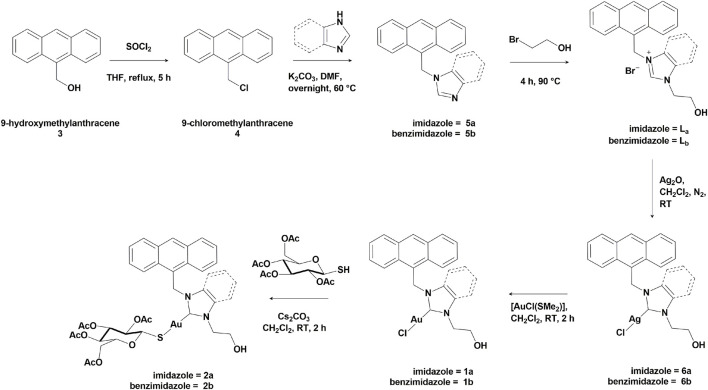
Synthetic route for the Au(I) complexes **1a-b** and **2a-b**.

The overall synthetic route for the preparation of the gold(I) complexes **1**-**2** is reported in Scheme 1.

#### 2.1.1 Synthesis of the ligands **L**_**a**_ and **L**_**b**_

The synthetic route for the obtainment of ligands **L**_**a**_ and **L**_**b**_ is reported in Scheme 1. The preparation of 9-chloromethylanthracene (**4**) was carried out following the procedure described in the literature (Fabbrini et al., 2019), that involves refluxing 9-hydroxymethylanthracene (**3**) and SOCl_2_ in THF under inert atmosphere for 5 hours. 9-chloromethylanthracene (**4**) was obtained as a light-yellow powder with 90% yield. The obtained NMR spectra were consistent with the ones reported in the literature (Fabbrini et al., 2019). Subsequently, **4** was reacted with imidazole in order to obtain compounds **5a**, and with benzimidazole to obtain compound **5b** (Scheme 1). A base (K_2_CO_3_) was used to allow the functionalization on the *N*-position, in DMF at 60 °C overnight, following the procedure described in the literature (Guarra et al., 2020). Both compounds **5a** and **5b** were obtained as yellow solids, with a yield of 80% and 84%, respectively.

To obtain ligands **L**_**a**_ and **L**_**b**_, compounds **5a** and **5b** were reacted with a large excess of bromoethanol, used as the reaction solvent, for 4 h at 90 °C, checking the reaction progress through ^1^H-NMR. After the necessary time had passed, the solution was dried under reduced pressure, a few milliliters of methanol were added, and then tetrahydrofuran was slowly added to allow a brownish solid to precipitate. After the solid had been washed with abundant THF, the final product was obtained, with a yield of 80% and 56% respectively for **L**_**a**_ and **L**_**b**_. The two solids both present a light-brown color and hygroscopic properties.

#### 2.1.2 Synthesis of Au(I) complexes **1a** and **1b**

The selected strategy to synthesize the gold(I) carbenes **1a** and **1b** was transmetalation through the corresponding silver(I) carbene, as shown in Scheme 1. Ag_2_O acts both as a base to deprotonate the acidic proton of the ligand and as a silver(I) precursor to insert the metallic center. The use of silver bases is a well-known synthetic strategy, and Ag_2_O is by far one of the most commonly used (Garrison and Youngs, 2005). Thus, coordination to silver was carried out through the treatment of the bromide salts **L**_**a**_ and **L**_**b**_ with Ag_2_O. An indication for the carbene obtainment was the disappearance of the characteristic acidic H signal in the ^1^H-NMR spectrum. A side product, the corresponding biscarbene, was found to form during this metalation step. Usually, to shift the reaction towards monocarbene formation, two main strategies are adopted: adjusting the solvent polarity, with CH_2_Cl_2_ preferred over CH_3_OH, and the use of an excess of Ag_2_O. Although both precautions were used in this reaction, a small amount of biscarbene was found to form along with the desired monocarbene product. The presence and the percentage of biscarbene was confirmed through NMR and elemental analysis; in particular, the monocarbene/biscarbene ratio for **6a** was 7:2, while for **6b** was 3:2. The reaction conditions were 0.7 equivalents of Ag_2_O in CH_2_Cl_2_, under inert atmosphere. The ideal reaction time is different for the two products: 16 h to obtain complex **6a** and 1 h to obtain complex **6b**. Since it was not possible to obtain pure **6a** and **6b** products, some purification techniques were considered.

For complex **6a** it was possible to obtain the purified monocarbene product by quickly washing the solid with 1 mL of water. In fact, since the corresponding biscarbene has a positive charge, it is soluble in water and it can be easily separated from the desired **6a** product.

The same purification process was not feasible with **6b**, since the additional benzene in the NHC scaffold renders the corresponding biscarbene insoluble in water. Unfortunately, monocarbene **6b** was found to readily convert into the corresponding biscarbene when kept in solution, even over relatively short periods of time. Removing the biscarbene side product from the solution pushes the equilibrium toward the formation of more biscarbene, especially when small amounts of methanol are present in the solution. In addition, excessive handling of the product for relatively long periods leads to its degradation.

Therefore, it was decided not to purify the product **6b** from the biscarbene side product, but, instead, to carry out the following step and only then purify the final gold(I)-based product **1b**. Gold(I) complexes, in fact, are more stable and more resistant than Ag(I) complexes to being manipulated for purification.

In order to obtain complexes **1a** and **1b**, 1 equivalent of [AuCl(S(CH_3_)_2_)] was used for both complexes, in CH_2_Cl_2_ for 2 h, and reacted. Then, the suspension was filtered over celite to eliminate the AgCl formed, concentrated, and a yellow solid was obtained through precipitation with diethyl ether. Complex **1a** was obtained as a yellow solid with a quantitative yield. The final product **1b** was obtained through purification by column chromatography on neutral aluminum oxide using a gradient elution from DCM to DCM/MeOH 95:5. The solid was dried and recollected as a fine bright-yellow powder, with a yield of 61%. The two complexes **1a** and **1b** were characterized through ^1^H-NMR, ^13^C-NMR and elemental analysis (SI, Supplementary Figures S10–S13).

#### 2.1.3 Synthesis of Au(I) complexes **2a** and **2b**

The gold(I) complexes **1a** and **1b** were conjugated to 2,3,4,6-tetra-*O*-acetyl-1-thio-β-D-glucopyranose, a biomimetic glucose substrate, through a nucleophilic substitution on the gold center, obtaining complexes **2a** and **2b** (Scheme 1). The reaction was conducted at room temperature for 2 hours in presence of Cs_2_CO_3_ as a base, then the suspension was filtered and concentrated. Finally, the product was obtained through precipitation, slowly adding hexane. Complexes **2a** and **2b** were obtained in the form of pale-yellow powders, with quantitative yields and characterized through ^1^H-NMR, ^13^C-NMR, and CHN elemental analysis (SI, Supplementary Figures S14–S17).

### 2.2 Solution studies

#### 2.2.1 Stability in DMSO/H_2_O

In pure water media, the complexes undergo precipitation phenomena in the minutes time range at 10^−5^ M concentration. The UV spectra of the four compounds can still be obtained to calculate the corresponding molar absorption coefficients (ε) in water. The stability of the systems can be increased by lowering concentration and/or adding small amounts of DMSO. A UV-Vis stability experiment of the complexes in a DMSO/H_2_O 1:1 solution was also performed and spectra were recorded for 24 h at 37 °C. In this medium, complexes **1a** and **2a** become fully stable over long periods of time (SI, Supplementary Figures S27 and S29). The benzimidazole-based complexes, i.e., complexes **1b** (SI, Supplementary Figure S28) and **2b** (SI, Supplementary Figures S30) still show some changes in the 275–285 nm absorption range, while no variations are observed in the anthracene region. There is an increase of the absorbance value over time, which is very limited for **1b**, and more significant in the case of **2b**. On the other hand, also for **2b** the profile can be considered stable in the first 3 h.

#### 2.2.2 LogP_O/W_ determination

LogP determination plays a crucial role in understanding the properties of a potential drug. LogP helps assess the compound’s lipophilicity, which impacts its solubility, permeability, uptake, and systemic distribution. This piece of information aids in predicting the drug’s absorption, bioavailability, and potential for crossing cellular membranes. From logP evaluation studies, the four **1a-b** and **2a-b** compounds were found to be rather lipophilic. The logP values have been reported in Table 1. The four new compounds are characterized by greater lipophilicity than auranofin, due to the presence of the anthracenyl moiety. A decrease in lipophilicity is observed when moving from the **1a-b** to **2a-b**, as expected from the presence of the thiosugar moiety. In addition, an increase in lipophilicity is noted when moving from the imidazole-based complexes (**1a** and **2a**) to their corresponding benzimidazole-based ones (**1b** and **2b**). Interestingly, all four complexes have a logP value within the range (0<logP<5) defined by Lipinski as optimal for a molecule to be considered a potential drug candidate (Lipinski et al., 1997).

**TABLE 1:** LogP_O/W_ values of complexes **1a-b** and **2a-b**.

Complex	LogP_O/W_
Auranofin	1.6^a^
**1a**	3.4
**1b**	4.7
**2a**	2.7
**2b**	3.9

^a^ LogP_O/W_ value retrieved from the literature. (Marzo et al., 2017).

### 2.3 Interaction with biomolecules

The mechanism of action of gold(I)-based drugs depends on the inherent soft acid character of the gold center. Consequently, biomolecules featuring functional groups with a soft base nature, such as thiols, selenols, or amines, emerge as promising targets for interaction with these complexes. Among the diverse arrays of biological targets, human serum albumin (HSA) stands out as the most abundant mammalian protein in the plasma, with a concentration of approximately 0.3 mM. Furthermore, HSA has gained attention due to its remarkable affinity for interacting with gold-based compounds, primarily through the free cysteine residue (Cys34), which can undergo modification through coordination to the gold metal center (Pratesi et al., 2018; Zoppi et al., 2020). Studying the interaction of gold complexes with HSA can provide useful information from several perspectives. First, it allows to understand whether the gold complexes can interact with a model protein with an exposed free cysteine. In addition, this parameter provides insight into the potential transport of the complexes in the bloodstream following administration. On this basis, interaction experiments between the complexes **1-2** and HSA were undertaken, using two main techniques, fluorescence emission and high-resolution ESI-MS.

#### 2.3.1 Fluorescence titrations

The interaction of the complexes **1a-b** and **2a-b** with human serum albumin model protein (HSA) was investigated through spectrofluorimetric titrations. HSA has an intrinsic fluorescence at λ_ex_ = 280 nm and maximum emission around λ_em_ = 340 nm. Excitation at 280 nm results in fluorescence of the metal complex, with an emission band extending from approximately 380 nm. For this reason, the titration was followed at λ_em_ = 330 nm to minimize possible signal interferences, i.e., following the protein signal change with negligible interference from the titrant. The titrations were performed starting from 10^−6^ M HSA solutions prepared in 2.5 mM sodium cacodylate buffer at pH = 7.0. An increasing amount of the gold(I) complex (from a 10^−3^ M stock solution in DMSO) was progressively added to the HSA solution, resulting in a final complex concentration of 1.12 × 10^−5^ M. The amount of DMSO in the titration solution does not exceed 1.1% of the total volume. Blank experiments confirmed that this limited DMSO content does not contribute to any signal change. The experiments showed a signal quenching that indicated interaction with HSA for the Au(I)-NHC complexes **1a** and **1b**; oppositely, no interaction was highlighted for the complexes **2a** and **2b**, functionalized with the 1-thio-β-D-glucose tetraacetate moiety. An example of these experiments is reported in SI, Supplementary Figures S32.

To enable a rough evaluation of the thermodynamic parameters of the metal complex/HSA system, the titrations were carried out at four different temperatures between 10.0 °C and 50.0 °C. The binding isotherm curve was plotted on a graph for each titration performed.

**FIGURE 2:**
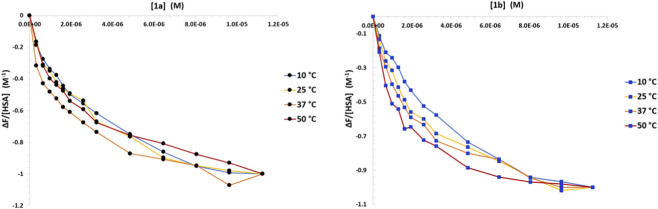
Average (mean of the three repetitions) binding isotherms for the interaction between HSA and **1a** (left) and **1b** (right) at different temperatures: 50 °C (red), 37 °C (orange), 25 °C (yellow), 10 °C (blue).

**FIGURE 3:**
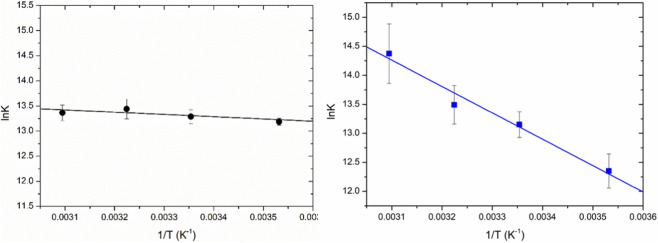
Graphical representation of Van’t Hoff equations for systems **1a**/HSA (left) and **1b**/HSA (right).

Figure 2 shows the binding isotherms’ averages at different temperatures for titrations with complex **1a** (left) and complex **1b** (right). A first qualitative observation is that, in the case of **1a**, no robust variation of the plots’ trend can be detected depending on T; on the other hand, for **1b**, the curvature seems to undergo a reliable decrease upon decrease of T. This finding qualitatively suggests that the behavior of the two systems is different and that the ΔH of the process is close to zero for **1a**/HSA, whilst different from zero for **1b**/HSA. To quantitatively analyze these data, the equilibrium constants (K) for the binding were calculated using the HypSpec® software. A 1:1 binding stoichiometry is found to be sufficient to describe the experimental data and such a model afforded the evaluation of the K values reported in Figure 3. In agreement with the previous comment, data confirm that these K values follow different trends for the titrations of **1a** or **1b**.

To further confirm the goodness of the 1:1 model used by HypSpec®, Equation 1 was used. Equation 1 is, indeed, an alternative form of the Scatchard equation and allows to evaluate *n*, intended here as the number of molecules bound to each protein.

$$\frac{C_{HSA}\left(C_{L}\Delta \varphi -\Delta F\right)}{\Delta F}=\frac{1}{nK_{app}}+\frac{\left(C_{L}\Delta \varphi -\Delta F\right)}{\Delta \varphi }\frac{1}{n}$$ (1)

Here, C_HSA_ is the total analytical concentration of HSA = [HSA] + [HSA-L], C_L_ is the analytical concentration of the metal complex L = [L]+[HSA-L], ΔF = F_HSA_ – (φ_HSA_C_HSA_), Δ φ = φ_HSA-L_–φ_HSA_, K_app_ = [HSA-L]/[HSA][L]. A straight line is displayed according to Equation 1 (SI, Supplementary Figures S33); n is the reciprocal slope, whereas K_app_ = slope/intercept.

For all titrations performed over the investigated temperature range, a value of n = 1 was obtained, confirming the adopted model.

Overall, complexes **1a** and **1b** exhibit different interaction modes with HSA. The binding constant K of complex **1a**, indeed, does not change with temperature, but remains stable around the same values. Conversely, complex **1b** shows a K with a rising trend as the temperature increases. Changing the temperature parameter allows to evaluate the enthalpy and entropy of the reaction using the Van’t Hoff equation. A detailed thermodynamic analysis of these systems is beyond the scope of the present work. On the other hand, an estimation of the ΔH and ΔS parameters, and in particular their sign, is essential to hypothesize a certain binding mode.

As a matter of fact, data interpolation according to the Van’t Hoff equation (Figure 3) show that **1a** has almost zero or indeterminate slope with respect to the calculated error (ΔH = 4 ± 2 kJ/mol, ΔS = 120 ± 10 J/molK), while **1b** presents a negative slope (ΔH = 37 ± 3 kJ/mol, ΔS = 230 ± 20 J/molK). Another element worth mentioning is that the lnK values at 25 °C and 37 °C for the two systems with **1a** and **1b** are quite similar.

The positive values of both ΔH and ΔS for the complex **1b**/HSA system are indicative of hydrophobic interactions, hinting at a predominant contribution of the benzimidazolic residue (Macii and Biver, 2021). The much higher ΔH obtained for **1b** agrees with the fact that **1b** possesses one more benzene cycle in its structure than complex **1a** and is, therefore, more hydrophobic. Overall, in agreement with the literature (Chaires, 2006; Macii and Biver, 2021), the thermodynamic signature of complex **1b** corresponds to hydrophobic forces. On the other side, for the imidazole-based complex **1a**, the interaction could be favored both by the hydrophobicity and electrostatic forces. Indeed, complexes **1a** and **1b** both feature an anthracenyl moiety that can engage in hydrophobic interactions, although this contribution is expected to be more significant for **1b**, which contains an additional benzene ring in its structure (Macii and Biver, 2021).

#### 2.3.2 HR-ESI-MS experiments

ESI mass spectrometry has become a valuable tool in recent years for characterizing adducts formed between metal-based complexes and biomolecules, especially proteins of various sizes (Massai et al., 2020; Zoppi et al., 2020). For comparison, the ESI-MS spectrum of HSA is provided (SI, Supplementary Figures S34). HSA mass spectrum presents a main peak at 66,436 Da, which corresponds to the molecular weight of the native protein, and two minor peaks corresponding to two post-translational modifications, i.e., cysteinylation on Cys34 at 66,555 Da, and glycosylation at 66,717 Da.

The reactions of complexes **1a-b** and **2a-b** with the protein HSA were investigated through ESI-MS measurements. The experiments were conducted according to a standard experimental setup, including preparing a protein solution (10^−4^ M) in 2 mM ammonium acetate at pH 6.8, adding a two-fold excess of the metal complex, and incubating for 4 h at 37 °C. The ESI mass spectra were subsequently recorded. All four complexes show interaction with HSA, as evidenced by the disappearance of the characteristic HSA peak at 66,436 Da and the appearance of new peaks corresponding to various adducts.

**FIGURE 4:**
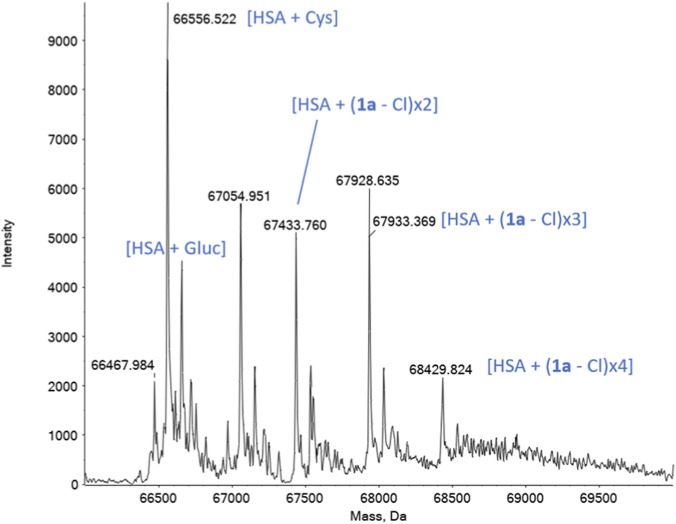
Deconvoluted ESI mass spectrum of HSA solution 10^−4^ M incubated for 4 h at 37 °C with **1a** (2:1 metal to protein ratio) in 20 mM ammonium acetate solution, pH 6.8.

Complex **1a** appears to form different adducts with multiple stoichiometries (Figure 4). In particular, it is possible to notice the presence of three new peaks, i.e. 67,434 Da, 67,933 Da, 68,430 Da. The mass increment present in these peaks is consistent with the mass of three different adducts, where the complex deprived of the chloride, [Au(NHC)]^+^, is present in different stoichiometries. In particular, two fragments of the complex are present in 67,434 Da, three fragments in 67,933 Da and four fragments in 68,430 Da. This type of coordination is coherent with previous studies conducted on auranofin and other analogues, where the [Au(PEt_3_)]^+^ fragment is the one observed in the adducts (Zoppi et al., 2020).

For complex **1b**, the spectrum reveals a new peak corresponding to an adduct (SI, Supplementary Figures S35). Also in this case, the fragment present is [Au(NHC)]^+^.

**FIGURE 5:**
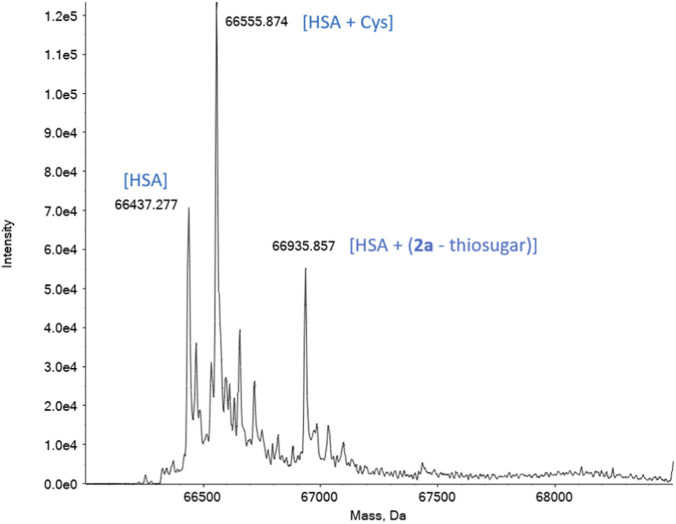
Deconvoluted ESI mass spectrum of HSA solution 10^−4^ M incubated for 4 h at 37 °C with **2a** (2:1 metal to protein ratio) in 20 mM ammonium acetate solution, pH 6.8.

In the case of both complexes **2a** and **2b**, it is possible to see two new peaks correlated with the presence of adducts between the complexes and albumin (66,936 Da in Figure 5 and 66,984 Da in Supplementary Figures S36). In particular, it is interesting to notice that the type of fragment present in the adducts is the same as in the experiments conducted with **1a** and **1b**, [Au(NHC)]^+^ (although with a different stoichiometry, in the case of **1a**). This result suggests that, in complexes bearing a thiosugar moiety, the Au–S bond is preferentially cleaved, while the Au–carbene bond remains intact. This observation suggests that the metal center in complexes **2a** and **2b**, despite being coordinated to a thiol ligand, retains a certain degree of reactivity that may enable interaction with free cysteine residues of the target proteins.

The results from fluorescence and ESI-MS tests indicate that the complexes form adducts with HSA through mechanisms that do not necessarily involve the same regions responsible for fluorescence quenching, reflecting different modes or sites of interaction.

### 2.4 Biological tests

#### 2.4.1 *In vitro* cytotoxicity

The *in vitro* cytotoxicity of complexes **1-2** was evaluated *via* the MTT assay in the human ovarian cancer cell lines A2780/S (cisplatin-sensitive), A2780/R (cisplatin-resistant), SKOV-3, and in the healthy cell line HSkMC (Human Skeletal Muscle Cells) after 72 h of incubation time. The resultant half-maximal inhibitory concentration (IC_50_) values (Riss and Moravec, 2004) are reported in Table 2. The IC_50_ values of auranofin and cisplatin are reported as well, the former as a comparison to evaluate the effectiveness of gold complexes, and the latter as a general reference for anticancer metal complexes. Complexes **1a** and **1b**, which do not present any passive-targeting moiety in their structures, exhibit good cytotoxicity on A2780/S cells, with IC_50_ values of 15 ± 3 μM and 7 ± 3 μM, respectively (Table 2). The value remains comparable also in cisplatin-resistant A2780 cells (A2780/R), confirming that gold complexes are able to bypass the resistance mechanisms against platinum-based complexes, a behavior aligned with that manifested by auranofin. Similar IC_50_ values are also exhibited in another type of human ovarian cancer cell line, SKOV-3. A particularly interesting result is the toxicity on the healthy cell line, HSkMC, used as a reference. Indeed, in that case, complexes **1a** and **1b** show very low toxicity, with IC_50_ values above 100 µM. This observation is particularly intriguing, given that gold-based complexes typically lack such selectivity, as exemplified by auranofin, which displays an IC_50_ of 10 ± 1 on HSkMC. However, when comparing auranofin with complexes **1a-b**, it should be taken into account that the selectivity index is comparable (>14 in the case of **1b**), or even lower (7 in the case of **1a**).

**TABLE 2:** Measured IC_50_ values (µM) obtained from cytotoxicity MTT assay after 72 h incubation period. The investigated cell lines are A2780/S (ovarian adenocarcinoma), A2780/R (ovarian adenocarcinoma cisplatin-resistant), SKOV-3 (ovarian cystadenocarcinoma) and HSkMC (human skeletal muscle cells, healthy cell line).

Complex		Cell line IC_50_ (µM) ± SD^a^				Selectivity index
		A2780/S	A2780/R	SKOV-3	HSkMC	HSkMC/A2780
Cisplatin		2.1 ± 0.2^b^	24.40 ± 0.10^c^	3.6 ± 0.8^d^	6.3 ± 0.8^d^	3
Auranofin		0.7 ± 0.2^d^	0.80 ± 0.05^d^	1.3 ± 0.5^d^	10 ± 1^d^	14
Free complexes	**1a**	15 ± 3	16 ± 3	17 ± 3	>100	>7
	**1b**	7 ± 3	7.2 ± 0.3	6.5 ± 0.4	>100	>14
Thiosugar conjugates	**2a**	5.5 ± 0.4	6.8 ± 0.2	4.3 ± 0.5	>100	>18
	**2b**	5.7 ± 0.7	5.0 ± 0.9	4.6 ± 0.6	>100	>18

^a^ Values are mean ± standard deviation (SD) of three biological independent experiments.

^b^ Value retrieved Casini et al. (2006).

^c^ Value retrieved Gamberi et al. (2021).

^d^ Value retrieved Giorgi et al. (2024).

In complexes **2a** and **2b**, where the conjugation with the thiosugar is present, two crucial elements can be extrapolated from the obtained outcomes. Firstly, the overall cytotoxic activity increases upon the introduction of thiosugar. This effect is particularly pronounced for imidazole-based complexes, where a three-fold decrease in IC_50_ is observed upon moving from **1a** to **2a** across all three cancer cell lines. As for the benzimidazole-based complexes, although a modest decrease in IC_50_ is observed from **1b** to **2b** in A2780/S, this difference should be considered marginal in light of the standard deviation associated with the IC_50_ value of **1b** in the same cell line. On the contrary, for the A2780/R and SKOV-3 cell lines, the IC_50_ decrease is evident after the introduction of the thiosugar, going from **1b** to **2b**. Interestingly, thiosugar-conjugated complexes **2a** and **2b** present the same cytotoxicity in the A2780/S and SKOV-3 cell lines, while there is a slight improvement of the cytotoxicity for **2b** in the A2780/R with respect to **2a**. A second important aspect is that the introduction of the thiosugar moiety does not affect the selectivity properties of complexes **2a** and **2b**. The latter, indeed, retain the property of their parent compounds not to affect healthy cells in the employed conditions. Considering also the lower IC_50_, the overall effect consists of an increased selectivity index (>18), which shows an improvement compared to auranofin.

#### 2.4.2 TrxR inhibition tests

**FIGURE 6:**
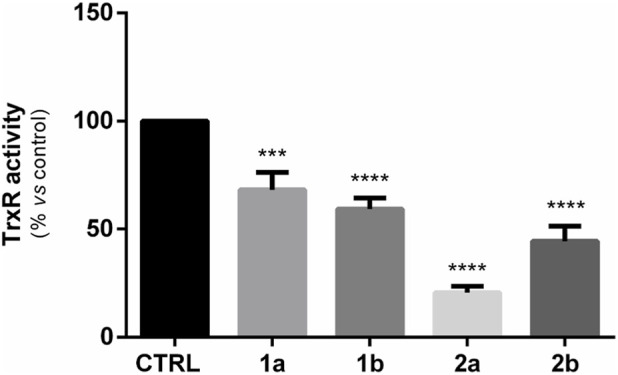
Measured TrxR activities after treatment for 6 h with **1a**, **1b**, **2a**, **2b**. TrxR activity is reported as a percentage compared to the control. The % ratio was done on U/mL values normalized for protein concentration (U/mg) (***p < 0.001; ****p < 0.0001).

The mechanisms of action behind the antiproliferative properties of gold complexes seem to involve the inhibition of TrxR. For these reasons, inhibition studies of TrxR were conducted on complexes **1a**, **1b**, **2a** and **2b**. The TrxR activity was evaluated through the colorimetric assay described in the experimental section, and the obtained results were collected after 6 h of treatment with the metal complexes at their 72 h-IC_50_ dose (Figure 6). The values obtained (U/mg) after treating the cells with the metal complexes have to be compared with a reference value (Control) that indicates the measured activity of the enzyme in untreated cells and is reported in Figure 6 as the percentage of enzyme activity relative to the control. The experiments show that all four gold(I)-based complexes **1a**, **1b**, **2a**, and **2b** cause inhibition of the enzyme TrxR. From the data shown in Figure 6, the inhibition effect of **2a**, which possesses a particular ability to inhibit the enzyme (nearly 20%), stands out in a superior way compared to the other complexes. This effect aligns with the cytotoxicity results, from which **2a** and **2b** appeared to be the most cytotoxic complexes. The outcome therefore indicates a correlation between stronger enzyme inhibition and increased cytotoxicity.

#### 2.4.3 Confocal microscopy experiments

**FIGURE 7:**
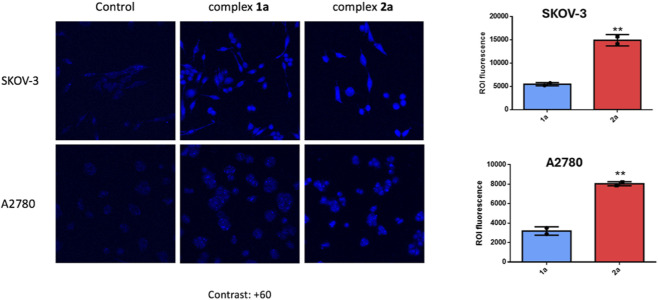
Fluorescence images, obtained through confocal microscopy, of A2780/S and SKOV-3 cells treated with complexes **1a** and **2a**, at a concentration of 10 µM for 20 min.

To investigate whether the different cytotoxic activity of the metal complexes was related to a difference in cellular drug accumulation, the uptake of the fluorescent compounds was first examined by confocal microscopy. The experiment was conducted in accordance with a previously reported protocol, with appropriate adaptations as required (Landini et al., 2017). Compounds **1a** and **2a** were evaluated on two ovarian carcinoma cell lines, A2780/S and SKOV-3. The results obtained *via* confocal microscopy are reported in Figure 7.

Both cell lines were treated with the compounds at a concentration of 10 µM for 20 min. Quantitative analysis using the ROI (region of interest) method revealed that compound **2a** exhibited higher fluorescence intensity than **1a** in both cell lines. Notably, in SKOV-3 cells, the fluorescence appeared more diffused, whereas in A2780/S cells it was more localized.

Regarding compounds **1b** and **2b**, the experiments were conducted on A2780/S cells treated with 10 µM of the respective complex for 20 min. As shown in Supplementary Figures S41, cells treated with **2b** displayed a stronger fluorescence signal compared to those treated with **1b**. The findings indicate that the complexes containing the thiosugar moiety, namely, **2a** and **2b**, are taken up by A2780/S and SKOV-3 cells more efficiently than their corresponding analogues lacking the thiosugar, **1a** and **1b**. Moreover, comparing the fluorescence intensities between cells treated with thiosugar-containing complexes **2a** and **2b**, a stronger signal was observed in those treated with the imidazole-based complex **2a** (Supplementary Figures S42).

#### 2.4.4 Fluorescence-activated cell sorting

**FIGURE 8:**
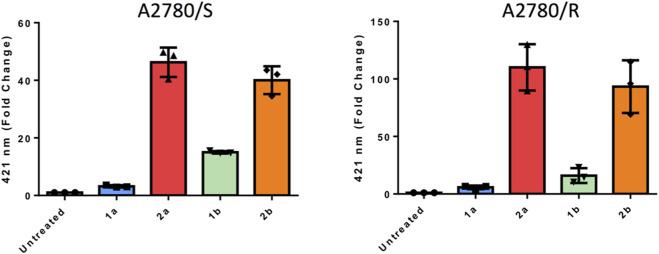
FACS results for the complexes **1a**, **2a**, **1b**, **2b** in the A2780/S cell line (left) and the A2780/R cell line (right).

To investigate the cellular uptake of the four compounds in A2780/S and A2780/R cell lines, fluorescence-activated cell sorting (FACS) analysis was carried out, taking advantage of the intrinsic fluorescence of the compounds. Cells were treated with the compounds at a concentration of 10 µM for 20 min. Figure 8 displays FACS results for both cisplatin-sensitive (A2780/S) and cisplatin-resistant (A2780/R) ovarian carcinoma cell lines. Notably, the thiosugar-conjugated compounds **2a** and **2b** displayed significantly higher cellular uptake in both cell lines compared to their non-conjugated analogues, **1a** and **1b.**

#### 2.4.5 GLUT inhibition tests

**FIGURE 9:**
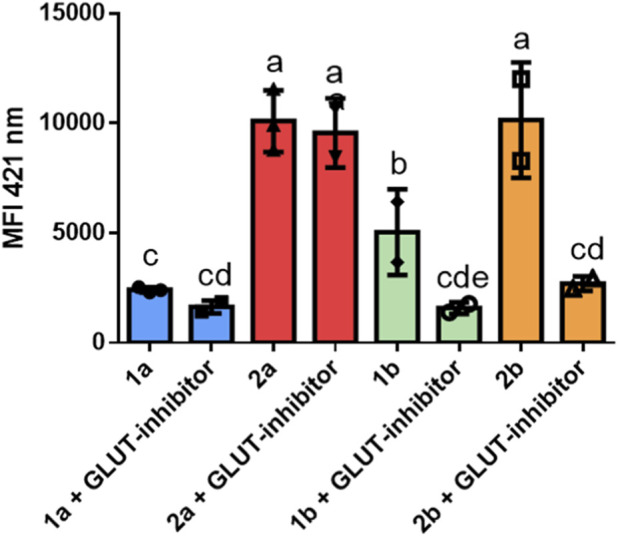
Median fluorescence intensity of A2780/S cells after co-treatment with the fluorescent compounds **1a**, **1b**, **2a**, **2b** and GLUT-1 inhibitor. Histograms with identical letters on the top are not significantly different, not having a *P*-value <0.05, according to the LSD post hoc test.

GLUT-inhibition tests were conducted to correlate the increased cytotoxicity of the thiosugar-conjugated complexes **2a** and **2b** with a specific targeting effect toward GLUT transporters overexpressed on the cancer cells’ membrane. A2780/S cells were pre-treated in adhesion with the GLUT-1 inhibitor 50 µM for 30 min. After detachment and suspension in PBS, the cells were treated with the fluorescent compounds (10 µM) and the GLUT-inhibitor (50 µM) for 10 min. Finally, the cells were washed in PBS and processed using the FACS procedure. The results obtained from FACS are reported in Figure 9, where histograms with identical letters on the top are not significantly different, not having a *P*-value <0.05, according to the LSD (Least Significant Difference) post hoc test.

The outcomes for benzimidazole-based complexes **1b** and **2b** seem to show a significant decrease in cells’ mean fluorescence intensity (MFI) after co-treatment with the GLUT-inhibitor compared to the MFI obtained after treatment with the cytotoxic complexes alone. The decrease in fluorescence intensity is two-fold and three-fold for complex **1b** and **2b**, respectively. The more pronounced decrease in the case of **2b** might suggest an entry partly mediated by GLUT-1 transporters operated by a recognition of the thiosugar moiety. However, it is essential to notice that, in the case of the imidazole-based complexes, this decrease in MFI is not significant for both complex **1a** and the complex with thiosugar, **2a**. This observation suggests that the increased cytotoxicity of complex **2a** compared with its thiosugar-free counterpart does not appear to be related to a targeting effect on glucose transporters, but other mechanisms of entry into the tumor cell are probably involved.

### 2.5 Peptide-gold complexes bioconjugates

Since conjugation with thiosugar enhanced the potency of the parent complexes without compromising their selectivity, conjugation with three distinct peptides, each targeting cancer cells through a different mechanism, was further investigated to evaluate whether this strategy could lead to even greater improvements. Three targeting peptides were selected from the literature.

**FIGURE 10:**
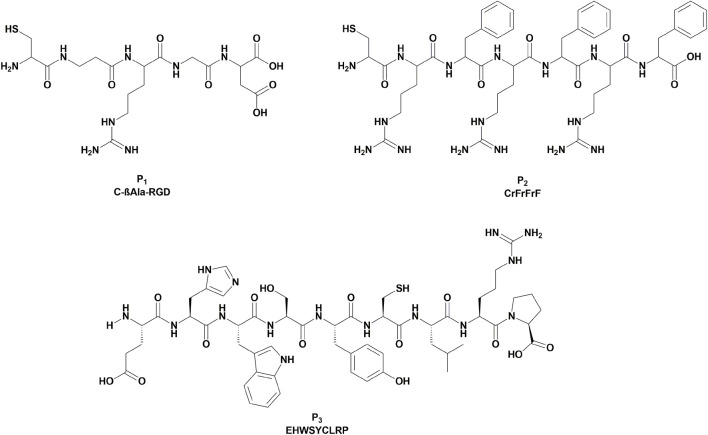
Chemical structures of the employed targeting peptides **P**_**1**_, **P**_**2**_, **P**_**3**_.

The first selected peptide is C-βAla-RGD (**P**_**1**,_ Figure 10). The selective interaction between the RGD-peptide and the integrin receptor can potentially lead to higher cytotoxicity and lower side effects, exploiting the selective recognition of the integrin receptor exposed on the cancer cell’s surface with a significant overexpression (Ahmad et al., 2021; Dal Pozzo et al., 2010; Fu et al., 2019; Hamidi et al., 2016; Hamidi and Ivaska, 2018). Various examples of metal-based anticancer complexes conjugated to a cyclic RGD according to this targeting strategy were reported in the literature (Dal Pozzo et al., 2010; Echigo et al., 2022; Fu et al., 2019; Gandioso et al., 2015; Hahn et al., 2017; Miura et al., 2013; Muhammad and Guo, 2014; Zhang et al., 2018; Zhao et al., 2019). Moreover, the alanine was inserted in the aminoacidic sequence as a spacer, while the cysteine was used for the conjugation to the gold complex.

The second selected peptide, CrFrFrF (**P**_**2,**_ Figure 10), allows preferential entry into cancer cells, exploiting the more significant potential difference of such cells’ mitochondrial membrane (Armstrong, 2006; Cullen et al., 2007; Erxleben, 2018; Horton et al., 2008). Indeed, mitochondria-targeting agents can overcome resistance mechanisms and activate cell-death programs (Köster et al., 2012).

The third selected peptide is an analogue of the LHRH peptide, EHWSYCLRP (**P**_**3**_, Figure 10), which had already demonstrated to retain the targeting properties of LHRH, despite the structural modifications (Dharap et al., 2005). The LHRH receptor, also known as gonadotropin-releasing hormone receptor, involved in growth and proliferation, is overexpressed in several cancer cell lines, such as prostate, endometrial, breast and ovarian cancer (Emons et al., 1990). The selective overexpression of LHRH receptors on cancer cells compared to healthy tissues makes them favorable targets for drug delivery (Ghanghoria et al., 2018).

**FIGURE 11:**
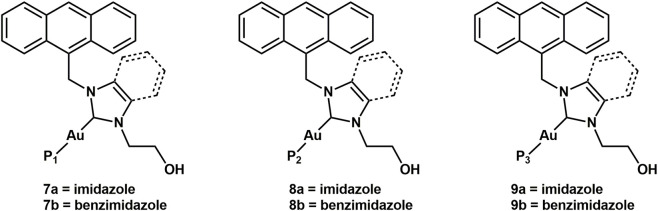
Chemical structures of the six bioconjugates **7-9**.

Thus, a panel of six bioconjugates (**7–9,** Figure 11) was synthesized.

#### 2.5.1 Synthesis

##### 2.5.1.1 Synthesis of peptides **P**_**1**_, **P**_**2**_, **P**_**3**_

The peptides **P**_**1**_ and **P**_**2**_ were obtained by a conventional Fmoc/tBu solid-phase peptide synthesis (SPPS) using the coupling reagent HBTU in the presence of DIPEA. This approach requires around 1 h for the coupling of each amino acid.

**P**_**3**_ was synthesized by microwave-assisted solid-phase peptide synthesis (MW-SPPS), a fully automated strategy able to reduce the synthesis time and the solvent demand. Using DIC (*N*,*N*′-diisopropylcarbodiimide) and Oxyma, both deprotection and coupling reactions can reach 90 °C and the peptide chain can be elongated in a very short time (around 4 min for each amino acid). Cys and His couplings were carried out at a lower temperature (50 °C) due to the increased risk of epimerization.

After the SPPS, the peptides were cleaved from the resin using trifluoroacetic acid in the presence of appropriate additives: triisopropylsilane, widely used to scavenge cations, and the almost odorless (3,6-dioxa-1,8-octanedithiol, DODT) as an alternative to the stinking thiols (i.e., EDT) for the cleavage of peptides containing cysteine. The peptides were characterized by LC-MS (SI, Supplementary Figures S18–S20). The MS characterization (*m/z*) is reported in Supplementary Table S1, SI.

##### 2.5.1.2 Synthesis of Au(I)-bioconjugates **7-9**

The synthesis of peptide-conjugates **7–9** (Figure 11) involves the treatment of complexes **1a** and **1b** with three different peptides, **P**_**1**_, **P**_**2**_, and **P**_**3**_, in DMF for 4 hours in the presence of TEA as a base, at room temperature and protected from the light. The solution was then dried under reduced pressure. The crude bioconjugate was purified by column chromatography on C18 reverse phase eluting with a mixture of water/acetonitrile, and the final product was analyzed through LC-MS (SI, Supplementary Figures S21–S26).

#### 2.5.2 *In vitro* cytotoxicity for bioconjugates 7-9

Cell viability experiments were performed for the bioconjugates **7-9** on A2780/S, A2780/R and SKOV-3 cancer cell lines (Table 3). In the context of complexes conjugated with the targeting peptides **P**_**1**_ and **P**_**2**_, the outcomes exhibit variations compared to the previous findings. Indeed, complexes **7a-b** and **8a-b** do not retain selectivity for cancer cells. The cytotoxic properties are modestly good, but unfortunately, there is no enhancement in the cytotoxic activity in comparison with the free complexes **1a** and **1b**.

**TABLE 3:** Measured IC_50_ values (µM) obtained from cytotoxicity MTT assay after 72 h incubation period. The investigated cell lines are A2780/S (ovarian adenocarcinoma), A2780/R (ovarian adenocarcinoma cisplatin-resistant), SKOV-3 (ovarian cystadenocarcinoma) and HSkMC (human skeletal muscle cells, healthy cell line).

Complex		Cell line IC50 (µM) ± SD^a^				Selectivity index
		A2780/S	A2780/R	SKOV-3	HSkMC	HSkMC/A2780
C-βAla-RGD bioconjugates	**7a**	13 ± 3	10.4 ± 1.9	21 ± 3	13 ± 2	1
	**7b**	15.3 ± 0.4	15.6 ± 1.7	28.0 ± 0.8	14 ± 3	1
CrFrFrF bioconjugates	**8a**	12 ± 3	9.2 ± 1.9	12 ± 2	13.3 ± 0.5	1
	**8b**	15.2 ± 0.6	14.8 ± 1.3	15.8 ± 1.6	12 ± 2	1
EHWSYCLRP bioconjugates	**9a**	7 ± 4	8.7 ± 1.9	14.2 ± 1.7	>100	>14
	**9b**	4.9 ± 1.6	6.7 ± 1.9	26 ± 4	>100	>20

^a^ Values are mean ± standard deviation (SD) of three biological independent experiments.

The conjugation of complexes **1a** and **1b** to the **P**_**3**_ peptide produced a result unaligned with that of the other peptides, showing a slight enhancement of the cytotoxic activity, especially for **9a** when compared to the IC_50_ of the corresponding **1a**, lacking the targeting moiety. Moreover, considering the IC_50_ for the A2780/S cell line, conjugates **9a** and **9b** show an improvement in the selectivity index compared to free complexes **1a** and **1b**, with a value greater than 14 for **9a** and greater than 20 for **9b**. Indeed, as well as the free complexes and the thiosugar conjugates, the **P**_**3**_ conjugates do not exhibit cytotoxic effects toward the tested healthy cell line. Comparing the cytotoxicity of **P**_**3**_-conjugated **9a–b** complexes with that of thiosugar-conjugated **2a–b** complexes, and taking statistical error into account, it is possible to observe that their cytotoxic properties are comparable in A2780/S and resistant cells, whereas in SKOV cells **9a–b** show diminished cytotoxicity.

It is useful to compare the biological activity and behavior of the peptide-conjugates **7-9** with that of previously reported gold-peptide bioconjugates. In the article published by Köster and collaborators in 2012, Au(I)-peptide conjugates bearing mitochondria-targeting sequences showed enhanced cellular uptake and cytotoxicity, which was also attributable to a peptide-mediated intracellular delivery (Köster et al., 2012). Another interesting paper was published by Doulain and coworkers, where gold(I) theranostic complexes functionalized with sugar or peptide biovectors were reported (Doulain et al., 2015). In this context, the nature of the biovector played a crucial role in the modulation of uptake, cytotoxicity, and selectivity. In contrast, in this study, the conjugation of Au(I)-NHC complexes to peptides **P**_**1**_ and **P**_**2**_ did not result in improved cytotoxicity or cancer cell selectivity compared to the non-conjugated complexes **1a** and **1b**. Only for the **P**_**3**_-conjugated complexes (**9a-b**) it was possible to notice a modest improvement in the cytotoxicity and selectivity. These results highlight the fact that conjugation with targeting peptides does not ensure an improvement in the biological activity.

Since the peptide–conjugated compounds **7a-b** and **8a-b** showed only modest activity, and **9a-b** did not display any advantage over their thiosugar-conjugated counterparts, further studies on this series of peptide-conjugated compounds were not pursued; therefore, the focus was shifted to the thiosugar conjugates.

## 3 Conclusion

Firstly, two gold(I) complexes were synthesized and characterized, which were subsequently conjugated to a passive targeting moiety, i.e., the thiosugar 2,3,4,6-tetra-*O*-acetyl-1-thio-β-D-glucopyranose.

Indeed, a panel of four thiosugar-conjugated gold(I) complexes with potential cytotoxicity and targeting action on ovarian cancer cells was obtained. The complexes were characterized through various techniques, including NMR spectroscopy, elemental analysis, and LC-MS, as appropriate.

Complexes **1a-b** and **2a-b** were studied in their interaction with a model protein bearing a free cysteine, HSA, by fluorescence titrations and interaction assays analyzed through ESI-MS. Fluorescence titrations showed that only complexes **1a** and **1b**, lacking thiosugar, were able to reversibly enter the protein pocket according to a process that is mainly hydrophobicity-driven. Conversely, mass spectrometry analysis revealed that all four complexes exhibited peaks corresponding to different adducts formed with the protein.

The four gold(I)-complexes were tested on A2780/S, A2780/R, SKOV-3 tumor cell lines, and the healthy HSkMC cell line. The four complexes **1a-b**, **2a-b** exhibit good cytotoxicity with values in the low micromolar range and excellent selectivity towards tumor cells. The analyzed healthy cell line is, indeed, not damaged. Interestingly, the thiosugar complexes **2a-b** show better cytotoxicity than complexes **1a-b** without the targeting moiety, with selectivity indexes higher than those of auranofin.

The data suggest that complexes **2a** and **2b** exhibit similar cytotoxic properties on the three cancer cell lines tested and have significant advantages over other compounds and even the progenitor of this compound class, auranofin, with excellent cytotoxicity and selectivity. Complex **2a** penetrates A2780 cells the most in experiments conducted with confocal microscopy and FACS. Furthermore, complex **2a** exhibits the best inhibition of thioredoxin reductase among the compounds tested in this study.

Through an experiment inhibiting the glucose transporter GLUT-1, however, it has emerged that complex **2a** does not appear to enter tumor cells through these channels. Therefore, no clear targeting activity towards these membrane proteins is observed, but the uptake and the consequent selectivity are related to different kinds of mechanisms. On the other side, in the same experiment benzimidazole-based complexes (**1b** and **2b**) seem to take advantage of the presence of GLUT channels when entering the cancer cell, and the effect is more important when the thiosugar moiety is present.

Subsequently, conjugation with three different targeting peptides [C-Ala-RGD (**P**_**1**_), CrFrFrF (**P**_**2**_), and EHWSYCLRP (**P**_**3**_)] selected from the literature was undertaken and cell viability studies were carried out on the six bioconjugates. For bioconjugates **7a-b** and **8a-b**, the IC_50_ values remain constant compared to the counterparts without the targeting moiety, while the selectivity is wholly lost. Unlike the other bioconjugates with peptides **P**_**1**_ and **P**_**2**_, bioconjugates **9a-b** retain the selectivity properties of complexes **1a-b**, with cytotoxicity properties comparable to those of the other bioconjugates in terms of potency, exhibiting excellent selectivity indexes (>14 for **9a** and >20 for **9b**). In conclusion, this study highlights the potential of rationally designed gold(I) complexes, particularly the two thiosugar-conjugated complexes **2a** and **2b**, as promising anticancer agents with high potency and selectivity. The conjugation of the thiosugar moiety significantly improved both cytotoxicity and cellular uptake of the gold(I) complexes, highlighting its key role in enhancing passive tumor selectivity. Although the GLUT-mediated entry mechanism seems to be predominant only for complex **2b**, the presence of the thiosugar appears to modulate cellular uptake also through alternative pathways, thereby contributing to the compounds’ excellent anticancer efficacy and favorable selectivity profile. Overall, these findings support further investigation of gold-based conjugates for selective cancer therapy.

## 4 Materials and methods

### 4.1 General remarks

Unless otherwise stated, the reactions were not performed under inert atmosphere. Vacuum (10^−2^ mmHg) was obtained with a mechanical oil pump. Solvents and reagents were purchased from Merck and used without previous treatment. When used anhydrous, the solvents were distilled and kept on molecular sieves. The gold(I) precursor [AuCl(S(CH_3_)_2_)] was synthesized according to the procedure reported in the literature (Brandys et al., 2000). K_2_CO_3_ was dried in the oven at 120 °C for 3 h and cooled in the dryer before use.

### 4.2 NMR experiments

All NMR spectra were acquired on a JEOL 400 YH spectrometer (resonating frequencies: 400, 160 and 100 MHz for ^1^H, ^31^P and ^13^C, respectively) and a 500 MHz (resonating frequencies: 500 and 125 MHz for ^1^H and ^13^C, respectively). All spectra were recorded at room temperature (25 ± 2 °C) in solvents with a deuteration degree of 99.8% and calibrated on solvent residual signals (Fulmer et al., 2010). All deuterated solvents were purchased from Deutero.de (https://www.deutero.de/). ^1^H, ^31^P and ^13^C characterization spectra were recorded in DMSO-d_6_, CDCl_3_, C_2_D_2_Cl_4_, as indicated.

### 4.3 General synthesis of the gold complexes

#### 4.3.1 Synthesis of 1-(9-anthracenylmethyl)imidazole **5a** and synthesis of 1-(9-anthracenylmethyl)benzimidazole **5b**

The synthesis has been carried out by adapting the procedure described in the literature (Guarra et al., 2020). 17.64 mmol of benzimidazole (or imidazole) and 19.40 mmol (2.682 g) of previously dried K_2_CO_3_ were suspended in 17 mL of DMF and stirred at room temperature for 30 min. Subsequently, a solution of 4.000 g of 9-(chloromethyl)-anthracene (17.64 mmol) in 37 mL of DMF was added dropwise in 30 min. The resulting suspension was heated up to 60 °C and stirred overnight. After cooling down the mixture, 140 mL of distilled water were added. The suspension was extracted with three aliquots of 230 mL of CH_2_Cl_2_. The combined organic phases were washed with distilled water for eight times and dried over Na_2_SO_4_. The orange solution was concentrated under reduced pressure and subsequently added dropwise to cold hexane, allowing the precipitation of a yellow microcrystalline solid. 3.650 g (yield = 80%) of 1-(9-anthracenylmethyl)imidazole **5a** were obtained, and 4.553 g (yield = 84%) of 1-(9-anthracenylmethyl)benzimidazole **5b**.

##### 4.3.1.1 1-(9-anthracenylmethyl)imidazole **5a**

^1^H-NMR (CDCl_3_, 500 MHz): δ (ppm) = 8.31 (s, 1H, anthracenyl H10), 7.94 (d, ^2^J = 8.5, 2H, anthracenyl, H1 and H8), 7.82 (d, ^2^J = 8.5, 2H, anthracenyl, H4 and H5), 7.34-7.26 (m, 5H, (1H imidazole NCHN and 4H anthracenyl H2, H3, H6 and H7)), 6.77 (s, 1H, imidazole, H4′), 6.62 (s, 1H, imidazole, H5′), 5.78 (s, 2H, imidazole-CH_2_-anthracenyl). ^13^C-NMR (CDCl_3_, 125 MHz): δ (ppm) = 137.06 (NCHN, imidazole), 131.65 (anthracenyl, 4a and 10a or 8a and 9a), 130.99 (anthracenyl, 4a and 10a or 8a and 9a), 129.70 (anthracenyl, C4, C5 and C10), 129.57 (imidazole C4′), 127.59 (anthracenyl, C3 and C6 or C2 and C7), 125.54 (anthracenyl, C3 and C6 or C2 and C7), 124.93 (anthracenyl, ^quat^C, C9), 123.20 (anthracenyl C1 and C8), 119.15 (imidazole C5′), 43.25 (imidazole-CH_2_-anthracenyl).

##### 4.3.1.2 1-(9-anthracenylmethyl)benzimidazole **5b**

^1^H-NMR (CDCl_3_, 400 MHz): δ (ppm) = 8.61 (s, 1H, anthracenyl H10), 8.11-8.07 (m, 4H, anthracenyl, H1, H8, H4, and H5), 7.84 (d, ^2^J = 8, 1H, benzimidazole H4′ or H7′), 7.70 (d, ^2^J = 8, 1H benzimidazole, H4′ or H7′), 7.53-7.50 (m, 4H, anthracenyl, H2, H3, H6, and H7), 7.45-7.35 (m, 2H, benzimidazole, H5′ and H6′), 7.26 (s, 1H, benzimidazole NCHN), 6.14 (s, 2H, benzimidazole-CH_2_-anthracenyl). ^13^C-NMR (CDCl_3_, 100 MHz): δ (ppm) = 144.17 (^quat^C), 142.46 (benzimidazole, NCHN), 134.49 (^quat^C), 131.74 (^quat^C), 131.35 (^quat^C), 130.06 (^quat^C), 129.84 (anthracenyl, C1 and C8 or C4 and C5), 127.88 (anthracenyl C2 and C7 or C3 and C6), 125.74 (anthracenyl, C2 and C7 or C3 and C6), 123.87 (benzimidazole, C5′ or C6′), 123.42 (benzimidazole, C5′ or C6′), 123.26 (anthracenyl C1 and C8 or C4 and C5), 122.81 (^quat^C), 120.83 (benzimidazole C4′ or C7′), 109.86 (benzimidazole C4′ or C7′), 41.66 (benzimidazole-CH_2_-anthracenyl).

#### 4.3.2 Synthesis of ligands **L**_**a**_ and **L**_**b**_

7.347 mmol of **5a** or **5b** were dissolved in 2.086 mL of bromoethanol, the solution was heated up to 90 °C and stirred for 4 h. After the cooling of the dark-brown mixture, 5 mL of methanol were added, and the obtained solution was added dropwise to 250 mL of THF, stirring at low temperature. The precipitation of the resulting light brown solid was aided by ultrasound and low temperature. The final solid product was filtered and washed with abundant THF. The resultant yield was 80% for **L**_**a**_ and 56% for **L**_**b**_.

##### 4.3.2.1 1-(9-anthracenylmethyl)-3-(2-hydroxyethyl)imidazolium bromide (**L**_**a**_)

^1^H-NMR (DMSO-d_6_, 400 MHz): δ (ppm) = 8.95 (s, 1H, NCHN), 8.85 (s, 1H, anthracenyl H10), 8.48 (d, ^2^J = 8.8, 2H, anthracenyl H1 and H8), 8.23 (d, ^2^J = 8.4, 2H, anthracenyl H4 and H5), 7.71-7.60 (m, 6H, anthracenyl H2, H3, H6, H7, and imidazole H4′ and H5′), 6.50 (s, 2H, anthracenyl-CH_2_-imidazole), 5.07 (b, 1H, OH), 4.13 (t, ^2^J = 4.8, 2H, imidazole-CH_2_-*CH*_*2*_-OH), 3.63 (m, 2H, imidazole-*CH*_*2*_-CH_2_-OH). ^13^C-NMR (DMSO-d_6_, 100 MHz): δ (ppm) = 135.93 (NCHN), 131.08 (anthracenyl, ^quat^C, C4a and C10a or C8a and C9a), 130.64 (anthracenyl, ^quat^C, C4a and C10a or C8a and C9a), 130.11 (anthracenyl C10), 129.40 (anthracenyl C4 and C5), 127.79 (anthracenyl C2 and C7 or C3 and C6), 125.59 (anthracenyl C2 and C7 or C3 and C6), 123.59 (anthracenyl, ^quat^C, C9), 123.46(anthracenyl C1 and C8), 122.93 (imidazole, C4′), 122.19 (imidazole, C5′), 59.21 (imidazole-CH_2_-*CH*_*2*_-OH), 51.55 (imidazole-*CH*_*2*_-CH_2_-OH), 44.79 (anthracenyl-CH_2_-imidazole).

##### 4.3.2.2 1-(9-anthracenylmethyl)-3-(2-hydroxyethyl)benzimidazolium bromide (**L**_**b**_)

^1^H-NMR (DMSO-d_6_, 400 MHz): δ (ppm) = 8.98 (s, 1H, NCHN), 8.92 (s, 1H, anthracenyl H10), 8.38 (d, ^2^J = 8, 2H, anthracenyl H1 and H8), 8.28-8.25 (m, 3H, anthracenyl H4 and H5, and 1C benzimidazole), 8.09 (d, ^2^J = 7.6, 1H, benzimidazole H4′ or H7′), 7.80-7.72 (m, 2H, benzimidazole or anthracene), 7.67-7.60 (m, 4H, benzimidazole or anthracene), 6.74 (s, 2H, anthracenyl-CH_2_-benzimidazole), 4.98 (s, 1H, OH), 4.40 (t, ^2^J = 4.8, 2H, imidazole-CH_2_-*CH*_*2*_-OH), 3.64 (t, ^2^J = 4.8, 2H, benzimidazole-*CH*_*2*_-CH_2_-OH). ^13^C-NMR (DMSO-d_6_, 100 MHz): δ (ppm) = 141.71 (NCHN), 131.67 (anthracenyl C10), 131.15 (2C, 1C benzimidazole and 1C anthracenyl), 131.05 (benzimidazole), 130.47 (^quat^C, anthracenyl), 129.45 (anthracenyl C4 and C5), 127.84 (anthracenyl, C2 and C7 or C3 and C6), 126.68 (benzimidazole), 126.60 (benzimidazole), 125.65 (anthracenyl, C2 and C7 or C3 and C6), 123.52 (anthracenyl C1 and C8), 122.01 (^quat^C anthracenyl, C9), 114.25 (benzimidazole C4′ or C7′), 114.13 (benzimidazole C4′ or C7′), 58.66 (benzimidazole-*CH*_*2*_ *-*CH_2_-OH), 49.35 (benzimidazole-CH_2_-*CH*_*2*_-OH), 43.53 (anthracenyl-CH_2_-benzimidazole).

#### 4.3.3 Synthesis of 1-(9-anthracenylmethyl)-3-(2-hydroxyethyl)imidazole-2-ylidene silver bromide complex **6a**

1.094 g of **L**_**a**_ (2.850 mmol) were suspended in 15 mL of dry CH_2_Cl_2_, under inert atmosphere of argon, then 462 mg (1.99 mmol) of Ag_2_O were added and the reaction vessel was kept in the absence of light. The suspension was stirred at room temperature for 40 min, after which a significant lowering of the quantity of Ag_2_O was noticed, with the formation of a light-brown solid. The suspension was filtered through celite to eliminate the excess of Ag_2_O. The solution was concentrated under reduced pressure, and diethyl ether was added to precipitate the solid. The resultant solid was filtered and washed with 2 mL of H_2_O for two times to obtain a brown solid (615 mg, yield = 44%). ^1^H-NMR (CD_2_Cl_2_, 400 MHz): δ (ppm) = 8.59 (s, 1H, anthracenyl, H10), 8.31 (d, ^2^J = 8.4 Hz, 2H, anthracenyl, H1 and H8), 8.08 (d, ^2^J = 8.4 Hz, 2H, anthracenyl, H4 and H5), 7.6-7.48 (m, 4H, anthracenyl, H2, H3, H6 and H7), 6.95 (s, 1H, imidazole), 6.55 (s, 1H, imidazole), 6.23 (s, 2H, anthracenyl-CH_2_-imidazole), 4.23 (t, ^2^J = 5.6 Hz, 2H, imidazole-CH_2_CH_2_OH), 3.91 (t, ^2^J = 5.2 Hz, 2H, imidazole-CH_2_CH_2_OH).

#### 4.3.4 Synthesis of 1-(9-anthracenylmethyl)-3-(2-hydroxyethyl)imidazole-2-ylidene gold chloride complex **1a**

The reaction was conducted protected from the light. The 1-(9-anthracenylmethyl)-3-(2-hydroxyethyl)imidazole-2-ylidene silver bromide complex **6a** (306 mg, 0.623 mmol) was dissolved in 15 mL of CH_2_Cl_2_, then 1 equivalent (183 mg) of [AuCl(S(CH_3_)_2_)] was added. The solution was stirred for 4 h at room temperature, checking the progress through ^1^H-NMR. Subsequently, the suspension was filtered through celite to remove the grey solid, then the celite filter was washed with abundant CH_2_Cl_2_. The yellow solution was concentrated, and cold diethyl ether was added to allow the precipitation of a yellow solid (298 mg, yield 89%) ^1^H-NMR (C_2_D_2_Cl_4_, 500 MHz): δ (ppm) = 8.62 (s, 1H, anthracenyl, H10), 8.30 (d, ^2^J = 8.5 Hz, 2H, anthracenyl, H1 and H8), 8.11 (d, ^2^J = 9 Hz, 2H, H4 and H5), 7.66-7.63 (m, 2H, anthracenyl, H2 and H7), 7.58-7.55 (m, 2H, anthracenyl, H3 and H6), 6.91 (s, 1H, imidazole, H4′), 6.37 (s, 1H, imidazole, H5′), 6.35 (s, 2H, anthracenyl-CH_2_-imidazole), 4.36 (t, ^2^J = 5, 2H, imidazole-CH_2_-*CH*_*2*_-OH), 4.04 (t, ^2^J = 5 Hz, 2H, imidazole, imidazole-*CH*_*2*_ *-*CH_2_-OH). ^13^C NMR (C_2_D_2_Cl_4_, 125 MHz): δ (ppm) = 169.73 (carbene, NCN), 131.17 (anthracenyl), 130.78 (anthracenyl), 130.06 (anthracenyl), 130.03 (anthracenyl), 129.48 (anthracenyl), 127.79 (anthracenyl), 125.44 (anthracenyl or imidazole), 123.54 (anthracenyl), 122.93 (anthracenyl or imidazole), 119.59 (anthracenyl or imidazole), 62.02 (imidazole-CH_2_-*CH*_*2*_ *-*OH), 53.45 (imidazole-*CH*_*2*_-CH_2_-OH), 47.08 (anthracenyl-CH_2_-imidazole). Elemental analysis for C_20_H_18_AuClN_2_O: [calc. %] C: 44.92; H: 3.39; N: 5.24 [found %] C: 44.53 H: 3.36; N: 4.98.

#### 4.3.5 Synthesis of 1-(9-anthracenylmethyl)-3-(2-hydroxyethyl)benzimidazol-2-ylidene gold chloride complex **1b**

500 mg of **L**_**b**_ (1.15 mmol) were suspended in 15 mL of dry CH_2_Cl_2_, under inert atmosphere of argon, then 0.806 mmol of Ag_2_O were added and the reaction vessel was kept in the absence of light. The suspension was stirred at room temperature for 60 min, after which a significant lowering of the quantity of Ag_2_O was noticed, with the formation of a light-brown solid. The suspension was filtered through celite to eliminate the excess of Ag_2_O. The solution was concentrated under reduced pressure, and diethyl ether was added to precipitate the solid. The solid was filtered and dried under reduced pressure. An aliquot of the solid was collected (0.802 mmol) and was dissolved in 15 mL of CH_2_Cl_2_, then, 1 equivalent (236.2 mg) of [AuCl(S(CH_3_)_2_)] was added. The solution was stirred for 4 h at room temperature, under argon, protected from the light, checking the progress through ^1^H-NMR. Subsequently, the suspension was filtered through celite to remove the grey solid, then the celite filter was washed with abundant CH_2_Cl_2_. The yellow solution was concentrated, and cold diethyl ether was added to allow the precipitation of a light-yellow solid. The final product was obtained through purification by chromatography on neutral aluminum oxide using a gradient elution from DCM to DCM/MeOH 95:5 (yield 61%). ^1^H-NMR (DMSO-d_6_, 400 MHz): δ (ppm) = 8.77 (s, 1H, anthracenyl, H10), 8.51 (d, ^2^J = 8.4 Hz, 2H, anthracenyl H1 and H8), 8.17 (d, 2J = 9.2 Hz, 2H, anthracenyl H4 and H5), 7.78 (d, ^2^J = 8 Hz, 1H, benzimidazole H4′ or H7′), 7.61-7.53 (m, 4H, anthracenyl, H2, H3, H6 and H7), 7.32 (t, ^2^J = 8 Hz, 1H, benzimidazole H5′ or H6′), 7.13 (t, ^2^J = 7.2 Hz, 1H, benzimidazole H5′ or H6′), 7.04 (d, ^2^J = 8.4 Hz, 1H, benzimidazole H4′ or H7′), 6.74 (s, 2H, anthracenyl-CH_2_-benzimidazole), 5.05 (b, 1H, OH), 4.54 (m, 2H, benzimidazole-CH_2_-*CH*_*2*_ *-*OH), 3.85 (m, 2H, benzimidazole-*CH*_*2*_-CH_2_-OH). ^13^C-NMR (DMSO-d_6_, 100 MHz): δ (ppm) = 178.06 (carbene, NCN), 133.72 (anthracenyl or benzimidazole), 132.96 (anthracenyl or benzimidazole), 131.01 (anthracenyl or benzimidazole), 130.98 (anthracenyl or benzimidazole), 129.55 (anthracenyl or benzimidazole), 127.25 (anthracenyl or benzimidazole), 125.32 (anthracenyl or benzimidazole), 124.84 (anthracenyl or benzimidazole), 124.15 (anthracenyl or benzimidazole), 124.09 (anthracenyl or benzimidazole), 124.00 (anthracenyl or benzimidazole), 123.80 (anthracenyl or benzimidazole), 112.92 (benzimidazole), 111.90 (benzimidazole), 60.18 (anthracenyl-CH_2_-imidazole), 51.19 (benzimidazole-CH_2_-CH_2_-OH), 46.17 (benzimidazole-CH_2_-CH_2_-OH). Elemental analysis for C_24_H_20_AuClN_2_O: [calc. %] C: 49.29; H: 3.45; N:4.79 [found %] C: 50.25; H: 3.47; N: 4.93.

#### 4.3.6 Synthesis of (1-(9-anthracenylmethyl)-3-(2-hydroxyethyl)imidazole-2-yliden) (3,4,5,6-tetra-O-acetyl-1-thio-beta-D-glucopyranose) gold complex **2a**

Complex **1a** (70.0 mg, 0.131 mmol) was dissolved in 7 mL of CH_2_Cl_2_ with 52 mg (1.1 equivalent) of 2,3,4,6-tetra-*O*-acetyl-1-thio-beta-D-glucopyranose and 640 mg (15 equivalents) of Cs_2_CO_3_. The suspension was stirred for 2 h at room temperature, in the absence of light. Subsequently, the suspension was filtered through celite to remove the Cs_2_CO_3_ in excess, then the celite filter was washed with abundant CH_2_Cl_2_. The light-yellow solution was concentrated and added dropwise to 30 mL of hexane at low temperature. The resultant solid was filtered and washed with 2 mL of cold diethyl ether for two times to eliminate the unreacted thiosugar. A pale-yellow solid was obtained (107 mg, yield 95%). ^1^H-NMR (C_2_D_2_Cl_4_, 400 MHz): δ (ppm) = 8.61 (s, 1H, anthracenyl H10), 8.38 (d, ^2^J = 8.8 Hz, 2H, anthracenyl, H1 and H8), 8.11 (d, ^2^J = 8.4 Hz, 2H, anthracenyl H4 and H5), 7.67-7.63 (m, 2H, anthracenyl, H2 and H7 or H3 and H6), 7.58-7.54 (m, 2H, anthracenyl, H2 and H7 or H3 and H6), 6.83 (d, ^2^J = 1.6, 1H, imidazole H4′), 6.41-6.39 (m, 3H, imidazole H5′ and anthracenyl-CH_2_-imidazole), 5.20-5.12 (m, 4H, glucopyranose), 4.42-4.40 (m, 2H, imidazole-CH_2_-*CH*_*2*_ *-*OH), 4.25-4.19 (m, 2H, glucopyranose), 4.04 (m, 2H, benzimidazole-*CH*_*2*_ *-*CH_2_-OH), 3.78-3.71 (m, 1H, glucopyranose), 2.76 (b, 1H, OH), 2.16 (s, 3H, thiosugar, CH_2_OCOCH_3_), 2.04 (s, 3H, thiosugar, OCOCH_3_), 2.01 (s, 3H, thiosugar, OCOCH_3_), 2.00 (s, 3H, thiosugar, OCOCH_3_). ^13^C-NMR (C_2_D_2_Cl_4_, 125 MHz): δ (ppm) = 182.43 (NCN), 170.78 (glucopyranose, O(*C=O*)CH_3_), 170.54 (glucopyranose, O(*C=O*)CH_3_), 170.11 (glucopyranose, O(*C=O*)CH_3_), 169.78 (glucopyranose, O(*C=O*)CH_3_), 131.18 (imidazole or anthracene), 130.80 (imidazole or anthracene), 129.83 (imidazole or anthracene), 129.39 (anthracenyl C4 and C5), 127.67 (anthracenyl, C2 and C7 or C3 and C6), 125.40 (anthracenyl C2 and C7 or C3 and C6), 124.14 (imidazole or anthracene), 123.16 (anthracenyl, C1 and C8), 120.83 (imidazole or anthracene), 119.57 (imidazole or anthracene), 83.02 (glucopyranose), 77.80 (glucopyranose), 75.64 (glucopyranose), 68.77 (glucopyranose), 62.79 (glucopyranose), 61.98 (imidazole-CH_2_-CH_2_-OH), 53.16 (imidazole-CH_2_-CH_2_-OH), 46.63 (anthracenyl-CH_2_-imidazole), 21.36 (glucopyranose, CH_2_O(C=O)*CH*_*3*_), 20.81 (glucopyranose, O(C=O)*CH*_*3*_), 20.73 (glucopyranose, O(C=O)*CH*_*3*_), 20.69 (glucopyranose, O(C=O)*CH*_*3*_). Elemental analysis of C_34_H_37_AuN_2_O_10_S: [calc. %] C: 47.34; H: 4.32; N: 3.25; S: 3.72 [found %] C: 47.86 H: 4.27; N: 3.07; S: 3.30.

#### 4.3.7 Synthesis of (1-(9-anthracenylmethyl)-3-(2-hydroxyethyl)benzimidazol-2-yliden) (3,4,5,6-tetra-O-acetyl-1-thio-beta-D-glucopyranose) gold complex **2b**

52 mg of complex **1b** were dissolved in 4 mL of CH_2_Cl_2_ with 35 mg (1.1 equivalents) of 2,3,4,6-tetra-*O*-acetyl-1-thio-beta-D-glucopyranose and 400 mg (15 equivalents) of Cs_2_CO_3_. The suspension was stirred for 2 h at room temperature, in the absence of light. Subsequently, the suspension was filtered through celite to remove the Cs_2_CO_3_ in excess, then the celite filter was washed with abundant CH_2_Cl_2_. The light-yellow solution was concentrated and added dropwise to 20 mL of hexane at low temperature, obtaining a powder. The solid was filtered and then washed with 1–2 mL of cold diethyl ether. The resulting light-yellow microcrystalline solid was dried under reduced pressure (yield 95%).

^1^H-NMR (DMSO-d_6_, 400 MHz): δ (ppm) = 8.76-8.72 (m, 3H, anthracenyl H10, H1 and H8), 8.17 (d, ^2^J = 8, 2H, anthracenyl, H4 and H5), 7.73 (d, ^2^J = 7.6, 1H, benzimidazole H4′ or H7′), 7.65 (t, ^2^J = 7.2, 2H,anthracenyl), 7.56 (t, ^2^J = 7.6, 2H, anthracenyl), 7.25 (t, ^2^J = 7.6 Hz, 1H, benzimidazole H5′ or H6′), 6.99 (t, ^2^J = 8, 1H, benzimidazole H5′ or H6′), 6.85 (s, 2H, anthracenyl-CH_2_-benzimidazole), 6.74 (d, ^2^J = 7.6 Hz, 1H, benzimidazole H4′ or H7′), 5.13-5.00 (m, 2H, glucopyranose), 4.86-4.76 (m, 2H, glucopyranose), 4.62-4.60 (m, 2H, benzimidazole-CH_2_-CH_2_-OH), 4.01-3.85 (m, 4H, glucopyranose and benzimidazole-CH_2_-CH_2_-OH), 3.48 (m, 1H, glucopyranose), 1.95 (s, 3H, glucopyranose OCO*CH*_*3*_), 1.92 (s, 3H, glucopyranose OCO*CH*_*3*_), 1.86 (s, 3H, glucopyranose OCO*CH*_*3*_), 1.82 (s, 3H, glucopyranose OCO*CH*_*3*_).

^13^C-NMR (DMSO-d_6_, 125 MHz): δ (ppm) = 189.75 (NCN), 169.99 (glucopyranose, O(*C=O*)CH_3_), 169.55 (glucopyranose, O(*C=O*)CH_3_), 169.19 (glucopyranose, O(*C=O*)CH_3_), 169.00 (glucopyranose, O(*C=O*)CH_3_), 134.28 (anthracenyl or benzimidazole), 132.74 (anthracenyl or benzimidazole), 130.95 (anthracenyl or benzimidazole), 130.91 (anthracenyl or benzimidazole), 129.52 (anthracenyl), 127.32 (anthracenyl), 125.36 (anthracenyl), 125.02 (anthracenyl or benzimidazole), 123.96 (anthracenyl), 123.82 (anthracenyl or benzimidazole), 123.77 (anthracenyl or benzimidazole), 112.98 (benzimidazole), 111.85 (benzimidazole), 81.66 (glucopyranose), 77.00 (glucopyranose), 74.30 (glucopyranose), 73.38 (glucopyranose), 68.52 (glucopyranose), 62.30 (glucopyranose), 60.82 (anthracenyl-CH_2_-benzimidazole), 50.62 (benzimidazole-CH_2_-CH_2_-OH), 46.35 (benzimidazole-CH_2_-CH_2_-OH), 20.85 (glucopyranose, O(C=O)*CH*_*3*_), 20.41 (glucopyranose, O(C=O)*CH*_*3*_), 20.36 (2 CH_3_, glucopyranose, O(C=O)*CH*_*3*_). Elemental analysis of C_38_H_39_AuN_2_O_10_S: [calc. %] C: 50.00; H: 4.31; N: 3.07; S: 3.51 [found %] C: 49.56 H: 3.94; N: 3.46; S: 3.19.

### 4.4 Synthesis of peptides **P**_**1**_, **P**_**2**_ and **P**_**3**_

Peptides **P**_**1**_ and **P**_**2**_ were synthesized by a conventional manual SPPS in a PTFE reaction vessel (20 mL) with a filter, following the Fmoc/tBu procedure on 0.1 mmol of the preloaded Fmoc-Asp(OtBu)-, Fmoc-Phe-Wang resin, respectively (0.22 mmol/g, 450 mg, 100–200 Mesh). After resin swelling in DMF (1 mL/100 mg of resin) for 40 min Fmoc-protected amino acids were introduced through the following protocol: Fmoc-deprotection with a solution of 20% piperidine in DMF (1 mL/100 mg of resin) (1 × 5 min +3 × 15 min); washes: DMF (3 × 3 mL); coupling reaction: Fmoc amino acids/HBTU/DIPEA (5/5/6 equiv) dissolved in DMF with a final dilution of 1 mL DMF for 100 mg of resin for 45 min at room temperature; washes: DMF (3 × 3 mL).

Peptide **P**_**3**_ was synthesized by a fully automated microwave-assisted (MW) SPPS on a Liberty Blue™ automated peptide synthesizer (CEM Corporation, Matthews, NC, United States) according to the Fmoc/tBu strategy on a Fmoc-Glu(OtBu)-Wang resin (0.22 mmol/g, 450 mg, 100–200 Mesh) in a 0.1 mmol scale. After resin swelling in DMF, Fmoc-amino acids were introduced through the following protocol: 1) Fmoc-deprotection in 20% piperidine in DMF; 2) washes (3×) with DMF; 3) couplings with Fmoc-amino acid (5 eq, 0.2 M in DMF), Oxyma pure (5 eq, 1M in DMF) and DIC (5 eq, 0.5 M in DMF) prepared in separated bottles; 4) washes (3×) with DMF. Both deprotection and coupling reactions were performed in a Teflon vessel with microwave energy and nitrogen bubbling, reaching 90 °C (Supplementary Table S2) except 50 °C for Cys and His coupling (Supplementary Table S3). Reaction temperatures were monitored by an internal fiberoptic sensor. The resin was exposed to the microwave-assisted cycle described in Supplementary Tables S2 and S3.

Final cleavage from the resin, with concomitant sidechains deprotection, was achieved by treatment of resin-bound peptide with a TFA/TIS/H_2_O/DODT solution (94:2:2:2, 1 mL mixture/100 mg of resin). The mixture was stirred for approximately 3 h at room temperature. The resin was filtered and then rinsed with TFA (2 × 1 mL). The peptide solution was added to the washes and the product was precipitated from this solution by the addition of ice-cold Et_2_O (30 mL). The precipitated material was washed with ice-cold Et_2_O (3 × 30 mL) and dried under vacuum. The solid peptide was then dissolved in H_2_O (5 mL), iced and lyophilized.

The crude peptide was purified by flash chromatography (Teledyne Isco) using a rediSep C18 gold column (15.5 g) at a flow of 30 mL/min^−1^. Peptides were characterized by RP-UPLC ESI-MS (Waters Acquity UPLC coupled to a Waters 3100 ESI-SQD MS) at a flow of 0.5 mL/min^−1^ (detection λ 215 and 254 nm) using a Luna Omega C18 column (1.6 micron, 50 × 2.1 mm) for **P2** and **P3** and using an Aeris widepore XB-C8 column (3.6 micron, 150 × 4.6 mm) at a flow of 1 mL/min^−1^ for **P1**. Experimental parameters are summarized in Supplementary Table S4, analytical data are reported in SI, Supplementary Figures S18–S20 and Supplementary Table S1.

### 4.5 General synthesis for the bioconjugates 7, 8, 9

A stock solution in DMF was prepared for the three peptides **P**_**1**_, **P**_**2**_, **P**_**3**_. 1 × 10^−3^ mmol of peptide, 1 equivalent of the appropriate complex (either **1a** or **1b**), and 10 equivalents of triethylamine were solubilized in 500 µL of DMF. The obtained solution was stirred for 4 h, then the solvent was evaporated under reduced pressure. The obtainment of the final products was checked through LC-MS. The yield is quantitative, and the final products were obtained as white semi-solids. The bioconjugates were obtained with purity above 95%.

### 4.6 LC-MS instrumentation and analysis for the characterization of the bioconjugates

In order to characterize the bioconjugates **7-9**, LC-MS tests were carried out. LC-MS analyses were performed using a 1290 Infinity II LC system coupled to a 6495 Triple Quadrupole mass spectrometer, equipped with a Jet Stream electrospray (ESI) ionization source (Agilent Technologies, United States). Chromatographic separation was achieved by using an Acquity UPLC Peptide BEH C18 column, (2.1 mm I.D. × 100 mm length, 1.7 µm, 130Å pore size) and a gradient elution at 0.3 mL/min with a mobile phase consisting of formic acid (0.1% v/v) aqueous solution (A) and 50:50 v/v methanol:acetonitrile (B). The composition of the mobile phase changed over time as follows: 5% (B) for 0.5 min, from 5% to 90% (B) in 10 min, isocratic for 2.5 min, re-equilibration to initial conditions in 0.1 min, and isocratic up to 18 min. The multisampler and the column compartment were set at 4 °C and 25 °C, respectively. The injection volume was 2 μL. The 6495 Triple Quadrupole mass spectrometer detector operated in ESI positive ionization and full scan (MS^2^Scan) acquisition mode. For all the analytes, the ESI operation conditions were: drying gas temperature of 240 °C, drying gas flow of 18 L/min^−1^, nebulizer gas pressure of 30 psi, sheath gas temperature of 360 °C, sheath gas flow of 12 L/min^−1^, capillary voltage of 3000 V. Fragmentor voltage was fixed at 380 V. MS^2^Scan spectra were acquired in the range 100 < *m/z* < 1,200. Hence, the obtainment of the final products was checked through the described method. **7a**: *m/z* 510 [M+2H]^2+^, 1,018 [M + H]^+^, 1,040 [M + Na]^+^. **7b**: *m/z* 535 [M+3H]^3+^, 809 [M+2H]^2+^, 820 [M + H + Na]^2+^, 1,068 [M + H]^+^. **8a**: *m/z* 383 [M+4H]^4+^, 510 [M+3H]^3+^, 765 [M+2H]^2+^, 776 [M + H + Na]^3+^, 787 [M + H + K]^2+^. **8b**: *m/z* 395 [M+4H]^4+^, 527 [M+3H]^3+^, 790 [M+2H]^2+^. **9a**: *m/z* 563 [M+3H]^3+^, 845 [M+2H]^2+^. **9b**: *m/z* 580 [M+3H]^3+^, 870 [M+2H]^2+^ (SI, Supplementary Figures S21–S26).

### 4.7 Fluorescence titrations with HSA

Lyophilized HSA was purchased from Merck and used without further purification or manipulation. Stock solution (10^–3^ M) of HSA was prepared by dissolving a known quantity of the solids in ultrapure water, and the molar concentration was assessed spectrophotometrically (*ε* = 36,000 M^−1^ cm^−1^ at 280 nm).

The fluorescence data were recorded with an LS55 Perkin-Elmer spectrofluorometer. The excitation light is provided by a pulsed Xenon lamp (50 Hz). The instrument has a jacketed cell holder providing temperature control within ±0.1 °C. All spectra were recorded using quartz cuvettes of 500 µL minimum volume. Fluorescence experiment conditions were carefully chosen (high dilution, wavelengths) and checked to ensure direct proportionality between reading and fluorophore concentration.

Fluorescent titrations follow the fluorescent species’ emission changes upon increasing addition of the quencher. A solution of HSA (10^−6^ M) in aqueous buffer (NaCacodylate 2.5 mM, pH = 7.0, T = 25.0 °C) was put in a cuvette. After measuring the spectrum of protein alone, increasing amounts of the metal complex were added directly to the cuvette containing the HSA solution. A spectrum was recorded upon each addition. The titration was followed by plotting the binding isotherm (*y*-axis: ΔF/C_HSA_ where ΔF = F- φ_HSA_*C_HSA_; *x*-axis: C_complex_). Once the binding isotherm reaches the plateau, the titration is concluded. All the spectra shown are corrected for dilution factors and for the inner-filter effect. Fluorescence emission was corrected for the inner filter effect according to Equation 2, where F_obs_ is the measured fluorescence, while A_λex_ and A_λem_ represent the absorbances at the excitation wavelength (280 nm) and at the emission wavelength (330 nm), respectively, of the mixture at any point of the titration; (Lakowicz, 2006); note that A_λex_ and A_λem_ refer to the process of absorption of the exciting and emitted light beam thus need to consider the dimensions of the fluorimetric cell (1.0 cm in the excitation direction, 0.2 cm in the emission direction).

$${\;F}_{corr}=F_{obs}\times 10^{\left(\frac{A_{\lambda ex}+A_{\lambda em}}{2}\right)}$$ (2)

The data are finally treated with the appropriate equations to obtain the desired thermodynamic parameters. The HypSpec software was purchased from Hyperquad (http://www.hyperquad.co.uk/). Three replicates for each titration were done.

### 4.8 LogP determination

LogP values were determined through a modification of the shake-flask method (Cirri et al., 2017). Water (50 mL, distilled after milli-Q purification) and n-octanol (50 mL) were shaken together for 72 h to allow saturation of both phases. About 1–3 mg of the complex were dissolved in a 15 mL Falcon tube in the octanol phase and then an equal volume of water was added. The mixture was manually shaken for 10 minutes and then centrifuged for 5 minutes at 6,000 rpm to allow separation. Subsequently, 0.5 mL of each phase were moved in two different mineralization PE tubes together with 1 mL of metal-free concentrated nitric acid. The mixtures were heated overnight at 90 °C, then diluted with 4.5 mL of ultrapure water. Gold concentration in both phases was determined by ICP-OES. Reported logP is defined as log [complex]_o_/[complex]_w_. Final values were reported as the mean of three determinations.

### 4.9 ESI-MS acquisition for interaction tests with HSA

DMSO was purchased from Fluka. ESI-MS materials (water, methanol, and ammonium acetate) were purchased from Sigma-Aldrich. Lyophilized HSA was purchased from Sigma-Aldrich and used without further purification or manipulation.

Stock solution of HSA 10^−3^ M was prepared by dissolving the protein in LC-MS grade water. Stock solutions 10^−2^ M of the metal compounds were prepared by dissolving the samples in DMSO. Suitable amounts of the previously prepared stock solutions were mixed in an Eppendorf tube in a 2:1 metal:protein ratio and diluted with a 20 mM ammonium acetate solution until a final protein concentration of 10^−4^ M. The solutions were kept at 37 °C for 4 h and then ESI-MS analysis was carried out. All samples were diluted to a final concentration of 10^−6^ M for HSA with a 20 mM ammonium acetate and 0.1% of formic acid before injection into the mass spectrometer.

All the ESI mass spectra for interaction tests evaluation were recorded using a TripleTOF^®^ 5600^+^ high-resolution mass spectrometer (Sciex, Framingham, MA, United States), equipped with a DuoSpray^®^ interface operating with an ESI probe. The spectra were acquired through direct injection at a 7 μL/min^−1^ flow rate. In general, the ESI source parameters are as follows: positive polarity; ion-spray voltage floating (ISVF) 5500 V; temperature (TEM) 37 °C; ion source gas 1 (GS1) 40 L/min^−1^; ion source gas 2 (GS2) 0 L/min^−1^; curtain gas (CUR) 30 L/min^−1^; declustering potential (DP) 200 V; collision energy (CE) 10 V; acquisition range 800–1,600 *m/z*.

For acquisition, Analyst TF software 1.7.1 (Sciex) was used and deconvoluted spectra were obtained by using the Bio Tool Kit micro-application v.2.2 embedded in PeakView™ software v.2.2 (Sciex).

### 4.10 Biological experiments

#### 4.10.1 Cell culture

A2780 human ovarian cancer cell line was purchased from Creative Bioarray (NY 11967, United States): A2780/S sensitive to cisplatin (Lot N° CSC-C9491J) and A2780/R resistant to cisplatin (Lot N° CSC-C9492J); SKOV-3 human ovarian cancer cell line purchased from Cell Lines Service GmbH (Germany) (Lot 300342-1420SF), and normal human skeletal myoblasts (HSkMC) was purchased from Thermo Fisher Scientific Inc (Lot N° 2007603). A2780 and SKOV-3 cell lines were grown in RPMI1640 medium supplemented with 10% FBS, 1% glutamine and 1% antibiotics at 37 °C and sub-cultured twice weekly. Split 1: 5 (3–6 × 10^4^ cells per mL). Cryopreserved HSkMC cells were directly plated in DMEM medium supplemented with 10% FBS, 1% glutamine and 1% antibiotics and used for cytotoxicity experiments only. All cell lines have been routinely tested for *mycoplasma* (Lonza’s MycoAlert® *Mycoplasma* Detection Assays).

#### 4.10.2 *In vitro* cytotoxicity

RPMI 1640 cell culture medium, fetal bovine serum (FBS), and phosphate-buffered saline (PBS) were obtained from Euroclone (Milan, Italy). Thiazolyl blue tetrazolium bromide (MTT) was obtained from Merck Life Science (Milan, Italy). The human ovarian cancer cell lines A2780/S, A2780/R and SKOV-3 were maintained in RPMI-1640 supplemented with 10% FBS and antibiotics (penicillin, 100 U/mL; streptomycin, 100 μg/mL) at 37 °C in a 5% CO_2_ humidified atmosphere and subcultured twice weekly. Split 1:5 (3-6 × 10^4^ cells per mL). Cryopreserved HSkMC cells were directly plated in DMEM medium supplemented with 10% FBS, 1% glutamine and 1% antibiotics and used for cytotoxicity experiments only.

The cytotoxic effects of complexes in A2780/S, A2780/R, SKOV-3 and HSkMC cell lines were determined by using the colorimetric MTT (3-(4,5-dimethylthiazol-2-yl)-2,5-diphenyltetrazolium bromide) assay. Viable cells can metabolize MTT into formazan, a blue-colored compound that is detectable at 595 nm. Exponentially growing cells were seeded in 96-well plates at a density of 10^4^ cells/well. After 24 h, cells were treated with increasing concentrations of metal compounds ranging from 100 nM to 100 μM and each concentration was tested in triplicate. After 72 h, 0.5 mg/mL of MTT was added to each well and maintained at 37 °C for 1 h. Following precipitation, blue formazan was dissolved in 100 μL of DMSO, and the optical density was read in the microplate reader BioTek Synergy H1 (Agilent). From the absorbance measurements, the half-maximal inhibitory concentration (IC_50_) value of each compound on ovarian cancer cells was calculated using GraphPad Prism software version 6.0 (Graphpad Holdings, LLC, Boston, MA, United States). Each MTT assay was performed at least in triplicate and the results are reported in Tables 2 and 3 as IC_50_ mean values ±standard deviation (SD).

#### 4.10.3 TrxR activity inhibition

Exponentially growing cells were seeded in 20 mm^2^ Petri dishes at a density of 6 × 10^5^ cells/dish for 24 h. Thereafter, A2780 cells were treated with metal compound concentrations corresponding to their 72 h exposure IC_50_ doses for 24 h. At treatment, cells were lysed with RIPA buffer (50 mM Tris-HCl, pH 7.5, 150 mM NaCl, 100 mM NaF, 2 mM EGTA, 1% Triton X-100) containing 10 μL/mL protease and phosphatase inhibitors (Sigma Aldrich). Lysates were centrifuged at 4 °C, 14,000 RPM for 15 min, and supernatants were collected. After protein quantification with Bradford Assay, 30 µg of proteins were used for the assay. TrxR activity was measured by using a commercial colorimetric assay kit (Sigma Aldrich CS0170) based on the reduction of 5,5′-dithiobis (2-nitrobenzoic acid) (DTNB) with NADPH to 5-thio-2-nitrobenzoic acid (TNB) at 412 nm. This kit also contains a solution of mammalian TrxR inhibitor, which is necessary to determine the reduction of DTNB due only to the TrxR activity, since even other enzymes present in biological samples could reduce this substrate. The statistical analysis was performed using GraphPad Prism software version 6.0 (Graphpad Holdings, LLC, Boston, MA, United States). Results were normalized to the cellular protein content and reported as the percentage of enzyme activity compared to the untreated cells (control). The experiments were carried out in triplicate.

#### 4.10.4 Confocal microscopy experiments

1 × 10^5^ cells were plated on glass coverslips. After 24 h, the cells were labelled with fluorescent compounds (10 µM) for 20 min and immediately observed. The emitted fluorescence was analyzed by using a confocal fluorescence microscope Leica TCS SP8. Quantitative analysis was performed using ImageJ software by applying a region of interest (ROI)-based single-cell analysis. Briefly, individual cells were manually identified and selected as ROIs, and the mean fluorescence intensity was measured for each single cell. Background fluorescence was subtracted from all measurements. For each experimental condition, at least five independent microscopic fields were analyzed, and fluorescence values were averaged over all analyzed cells. This approach allowed normalization of fluorescence intensity on a per-cell basis, thereby compensating for possible variations in cell number and cell density among different images and ensuring an accurate comparison between experimental conditions. The statistical analysis was performed using GraphPad Prism software version 6.0 (Graphpad Holdings, LLC, Boston, MA, United States).

#### 4.10.5 Fluorescence-activated cell sorting

To assess cellular uptake of complexes, the presence of the fluorescent tag was exploited and the FACS CANTO II flow cytometer was used. Briefly, 4 × 10^5^ cells were plated in 20 mm^2^ Petri dishes. After 24 h, cells were detached and labelled with 10 µM complexes at 37 °C for 20 min. Cells were then washed with PBS, aliquoted in FACS tubes and immediately subjected to cytofluorometric analysis. In particular, emission in the Pacific Blue channel (421 nm) was analyzed. The Median Fluorescence Intensity (MFI) of each sample was normalized using FSC-A (forward scatter), an index of cell size. The statistical analysis was performed using GraphPad Prism software version 6.0 (Graphpad Holdings, LLC, Boston, MA, United States).

#### 4.10.6 GLUT inhibition experiments

GLUT Transporter Inhibitor IV (3-Fluoro-1,2-phenylene bis(3-hydroxybenzoate)), also known as WZB117, purchased from Sigma Aldrich, was employed for this experiment. Cells were seeded as reported in FACS analysis (see above). After 24 h, cells were pre-treated in adhesion with 50 µM of the GLUT-1 inhibitor for 30 min. This dose was selected after viability test (data not shown) and correspond to the highest concentration that does not include cytotoxic effect. The cells were then detached, suspended in PBS and co-treated with the fluorescent compounds (10 µM) and the GLUT-1 inhibitor (50 µM) for 10 min. The cells were finally washed in PBS and processed using FACS (Fluorescence-Activated Cell Sorting) procedure described above. The statistical analysis was performed using GraphPad Prism software version 6.0 (Graphpad Holdings, LLC, Boston, MA, United States).

## Data Availability

The original contributions presented in the study are included in the article/Supplementary Material, further inquiries can be directed to the corresponding author.
