# Supplementary material for: Thiosugar-functionalized gold(I)-NHC complexes as selective anticancer agents for potential targeted therapy
Source: Front Chem. 2026 Feb 26;14:1724206. doi: 10.3389/fchem.2026.1724206 (PMC12978790; doi:10.3389/fchem.2026.1724206)
Supplement: Supplementary file 1 [file DataSheet1.docx]

Supplementary Material

Thiosugar-Functionalized Gold(I)-NHC Complexes as Selective Anticancer Agents for Potential Targeted Therapy

Ester Giorgi^1^, Tarita Biver^1^, Michele Mannelli^2^, Tania Gamberi^2^, Matteo Becatti^2^, Giuseppina Sabatino^3^, Elisa Peroni^4,5^, Olivier Monasson^4,5^, Damiano Cirri^1^, Chiara Gabbiani^1^, Alessandro Pratesi^1^*

^1^Department of Chemistry and Industrial Chemistry, University of Pisa, Pisa, Italy

^2^Department of Experimental and Clinical Sciences “Mario Serio”, University of Florence, Florence, Italy

^3^National Research Council (CNR) Crystallography Institute, Catania, Italy

^4^BioCIS UMR 8076, CNRS, CY Cergy Paris Université, Cergy Pontoise, France

^5^BioCIS UMR 8076, CNRS, Université Paris-Saclay, Orsay, France

**Content**

[1 NMR characterisation spectra 2](#_Toc215665725)

[2 LC-MS characterization spectra 11](#_Toc215665726)

[3 Stability studies in DMSO/H_2_O 16](#_Toc215665727)

[4 Fluorescence interaction studies with HSA 18](#_Toc215665728)

[5 Mass spectrometry interaction studies with HSA 19](#_Toc215665729)

[6 Cytotoxicity curves 21](#_Toc215665730)

[7 Confocal microscopy studies 25](#_Toc215665731)

[8 Fluorescence-Activated Cell Sorting (FACS) studies 26](#_Toc215665732)

[9 Experimental parameters for peptide synthesis and purification 27](#_Toc215665733)

# NMR characterisation spectra


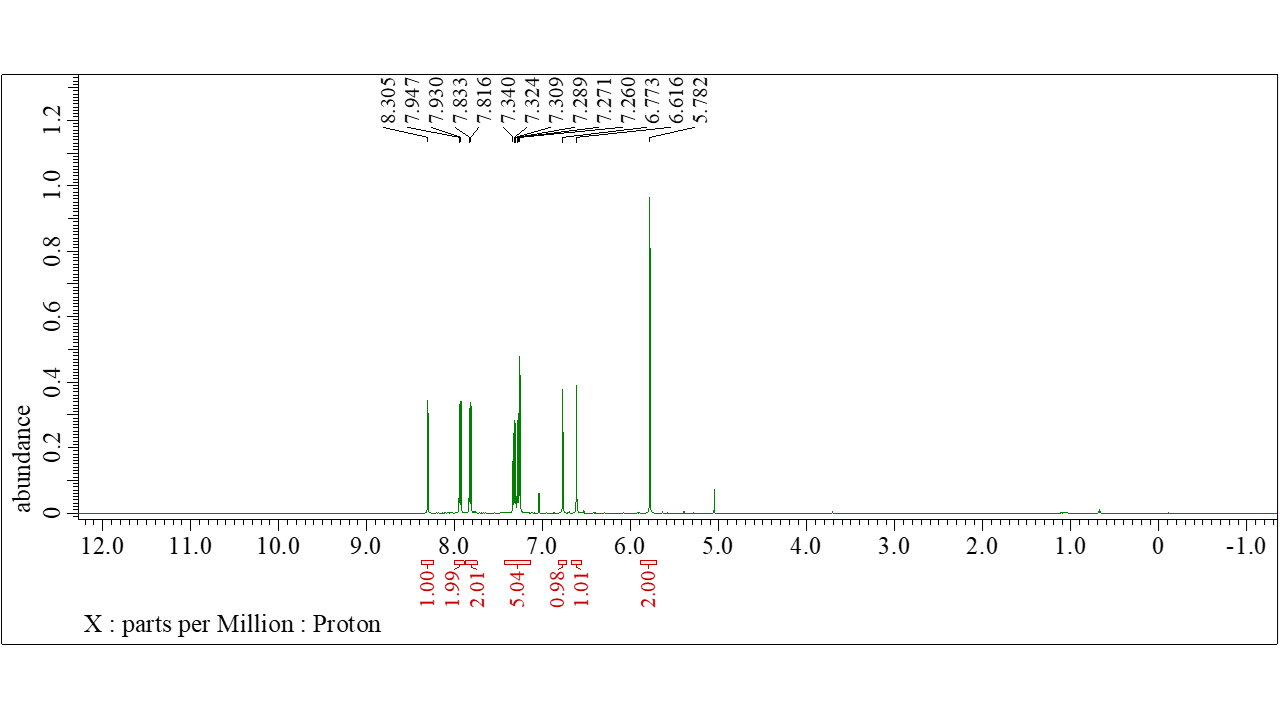


**Figure S1.** ^1^H-NMR spectrum of **5a** in CDCl_3_


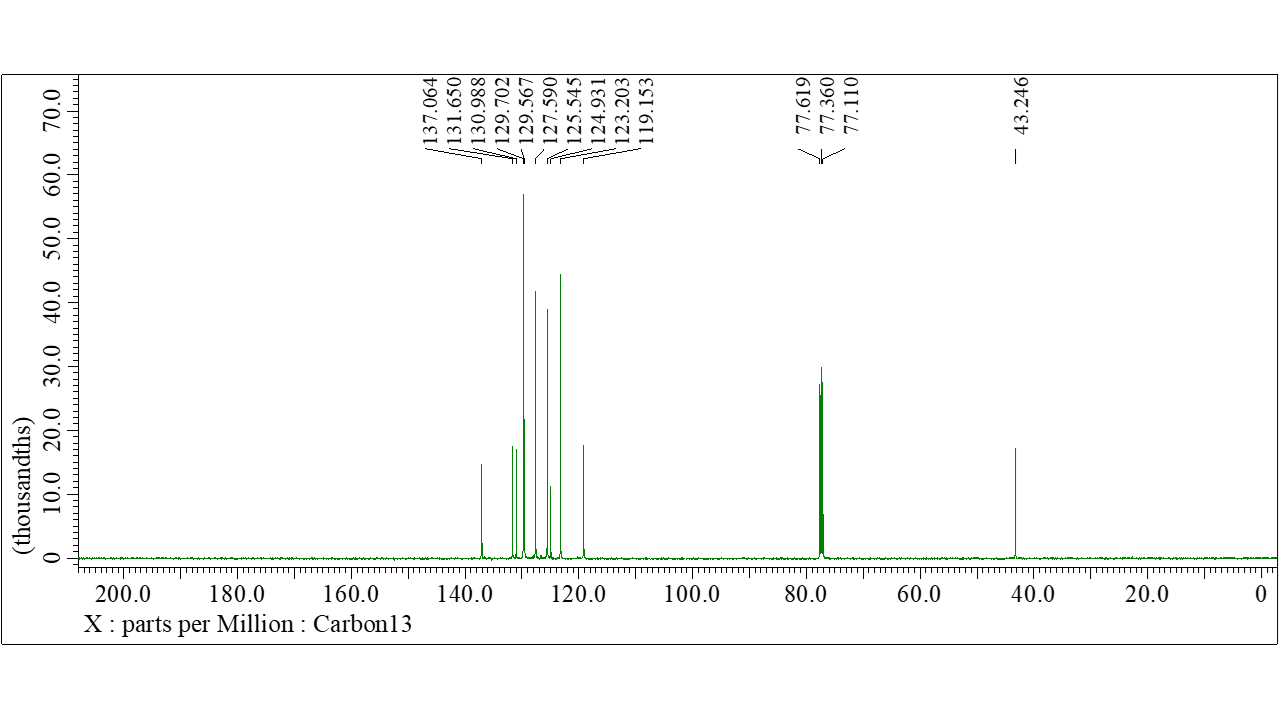


**Figure S2.** ^13^C-NMR spectrum of **5a** in CDCl_3_


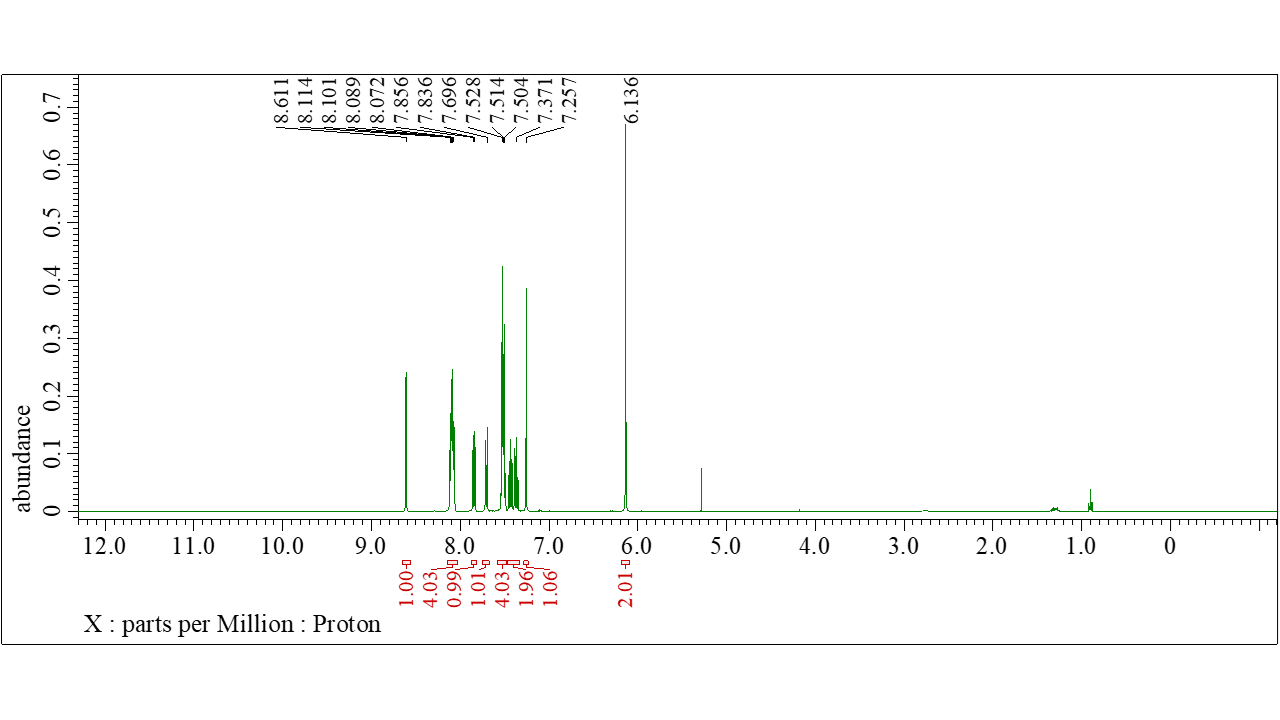


**Figure S3.** ^1^H-NMR spectrum of **5b** in CDCl_3_


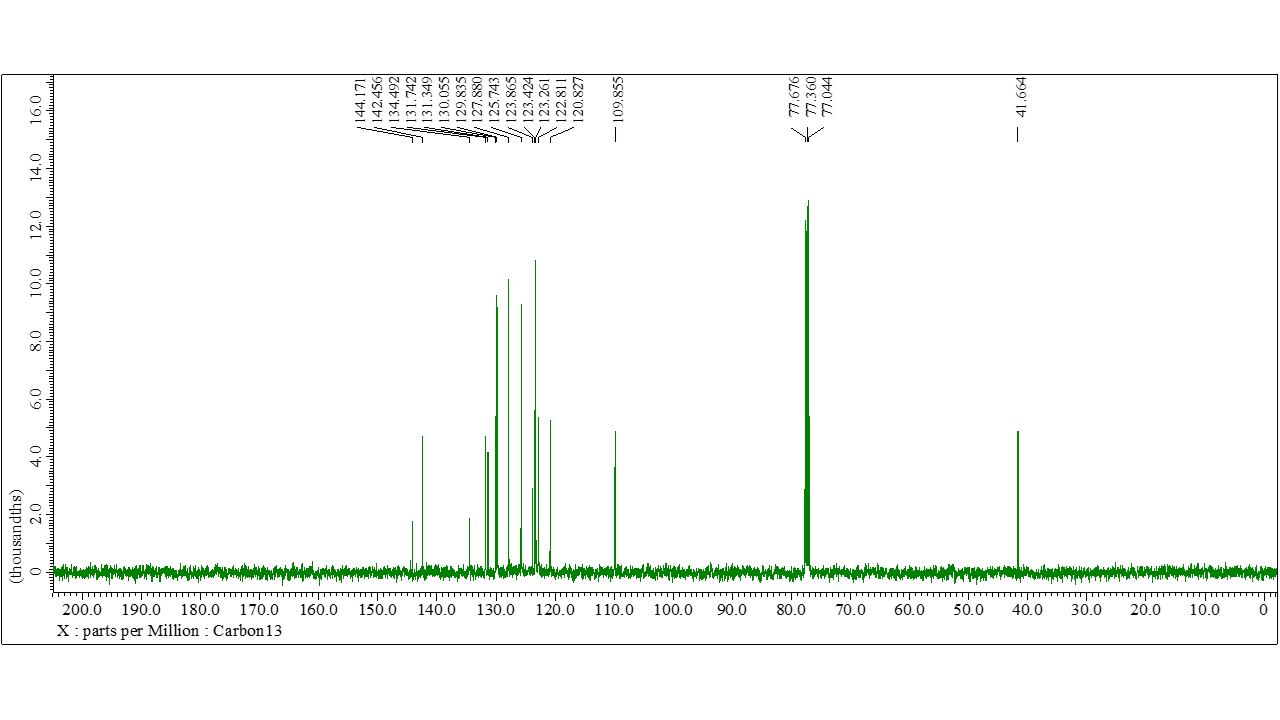


**Figure S4.** ^13^C-NMR spectrum of **5b** in CDCl_3_


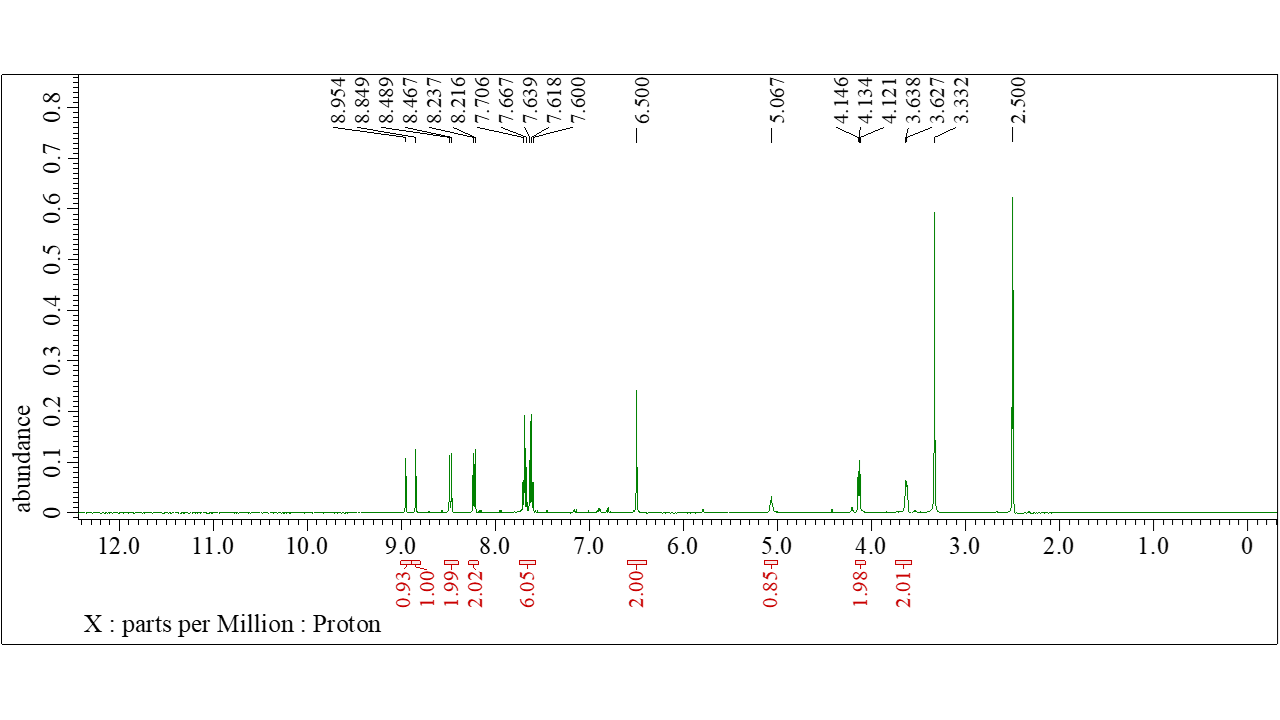


**Figure S5.** ^1^H-NMR spectrum of **L_a_** in DMSO-d_6_


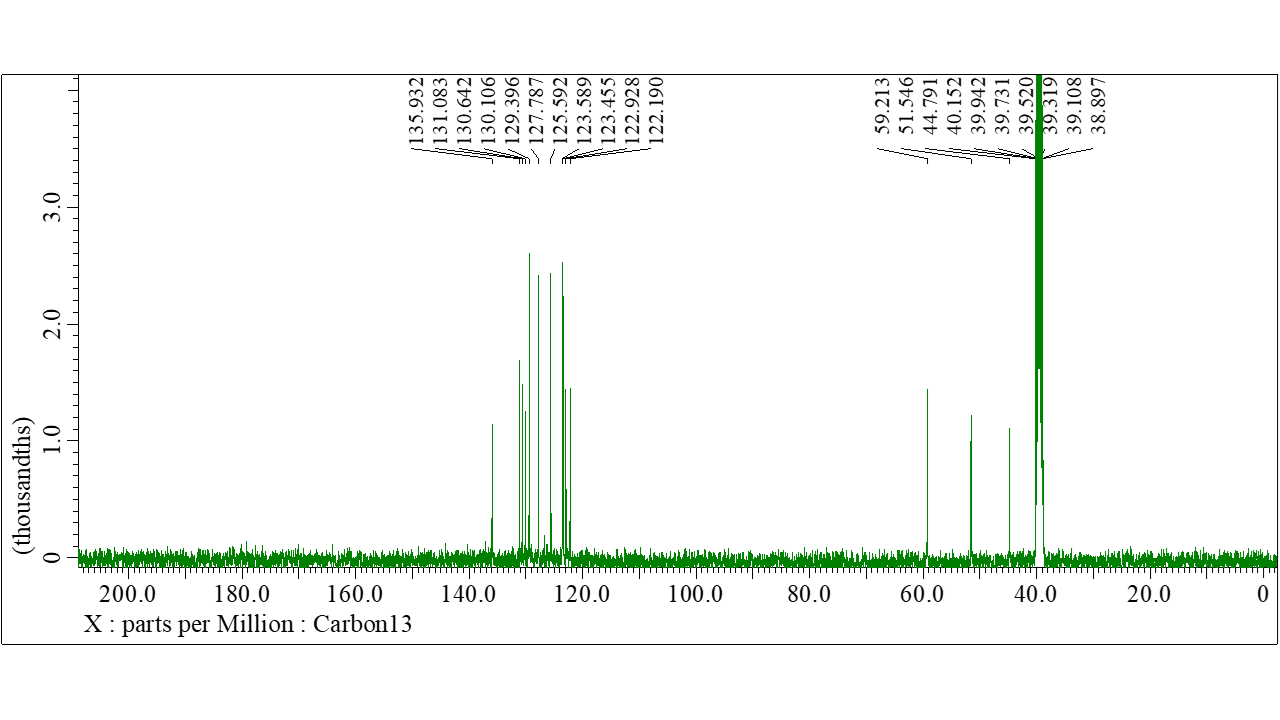


**Figure S6.** ^13^C-NMR spectrum of **L_a_** in DMSO-d_6_

*
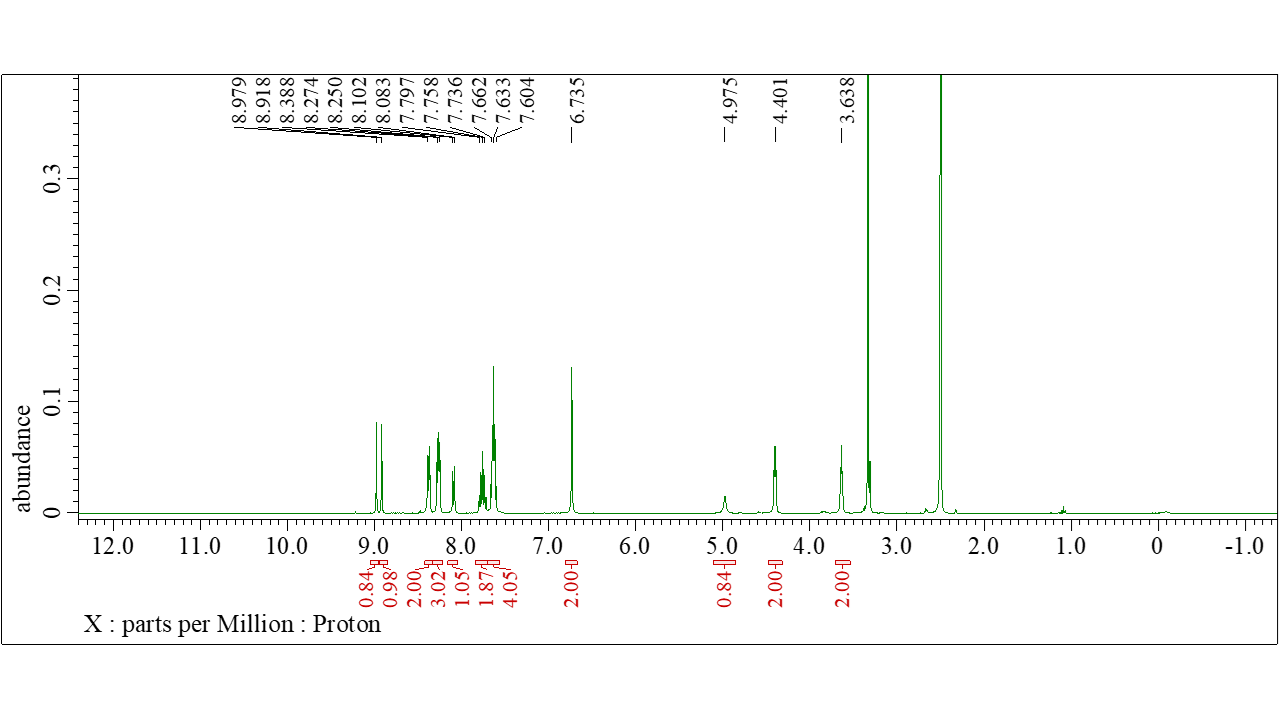
*

**Figure S7.** ^1^H-NMR spectrum of **L_b_** in DMSO-d_6_

*
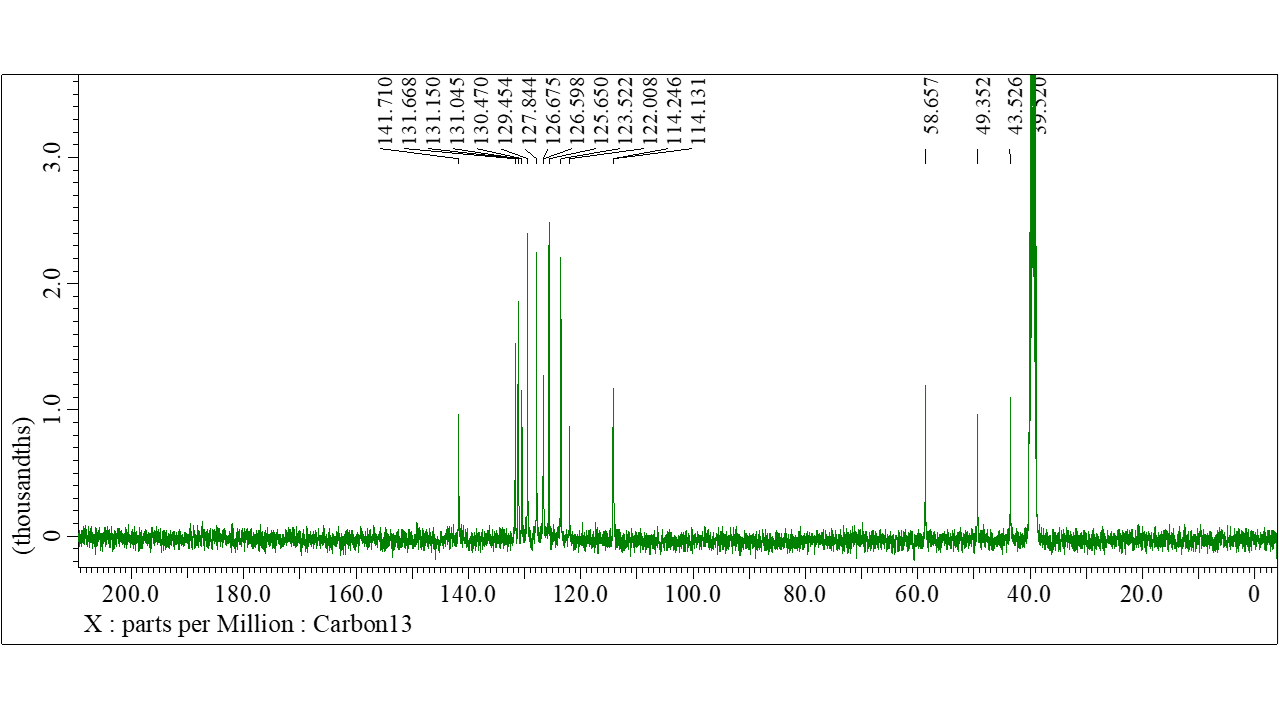
*

**Figure S8.** ^13^C-NMR spectrum of **L_b_** in DMSO-d_6_

__

**Figure S9.** ^1^H-NMR spectrum of **6a** in CD_2_Cl_2_


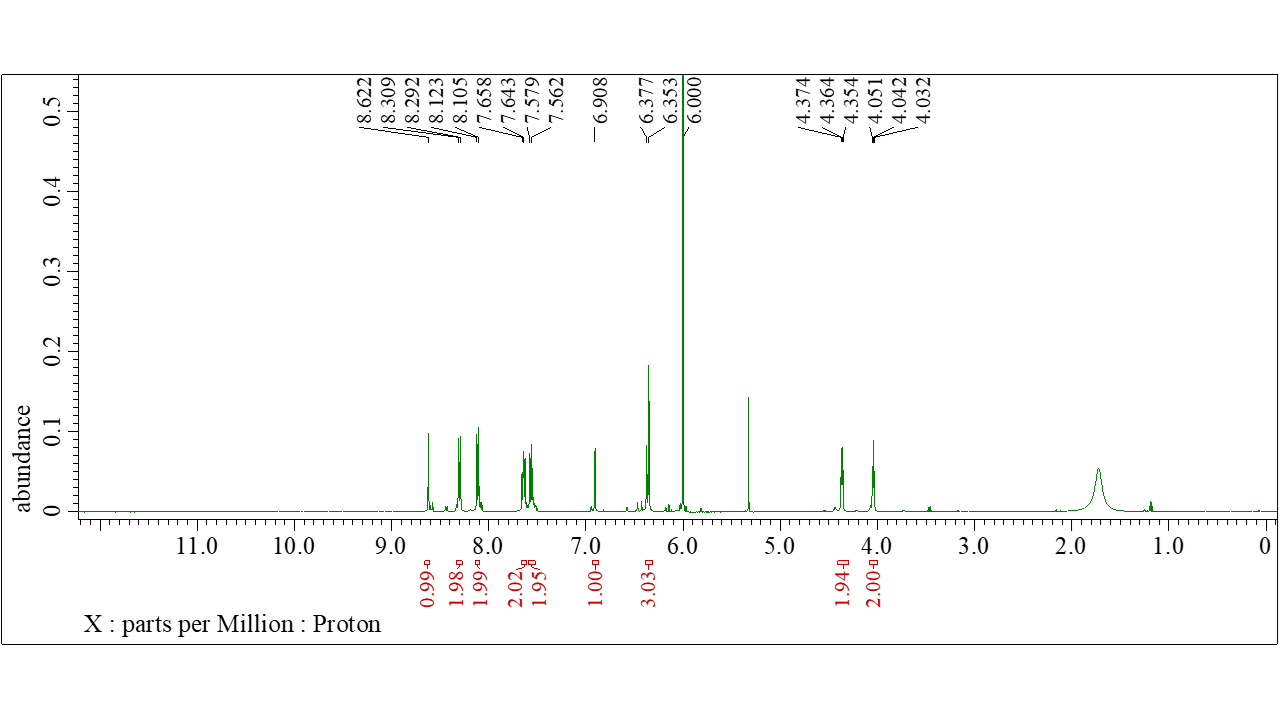


**Figure S10.** ^1^H-NMR spectrum of **1a** in C_2_D_2_Cl_4_


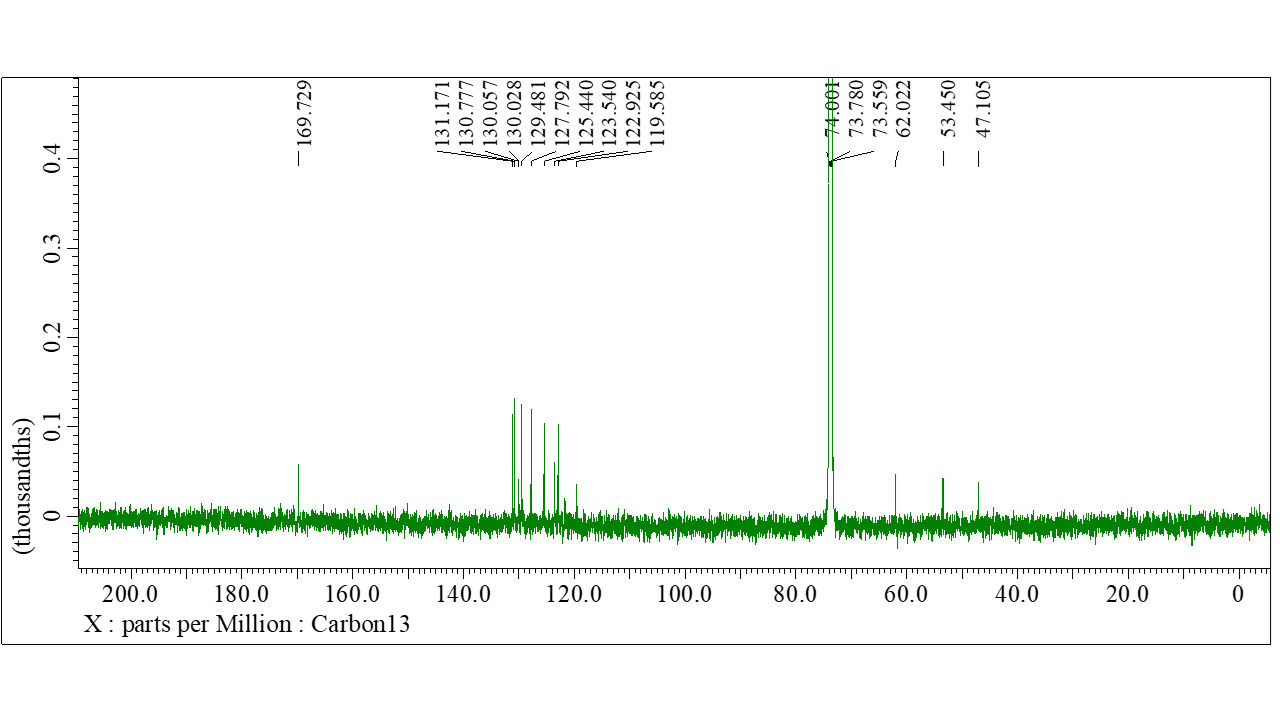


**Figure S11**. ^13^C-NMR spectrum of **1a** in C_2_D_2_Cl_4_


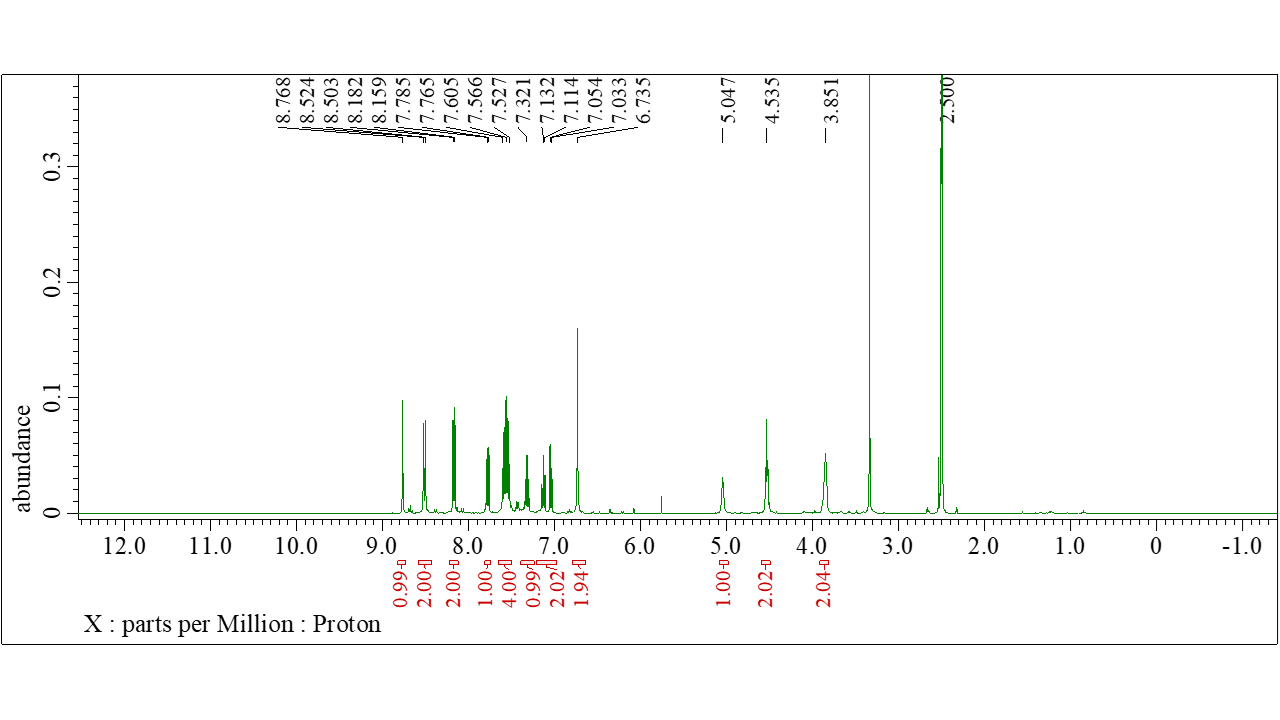


**Figure S12.** ^1^H-NMR spectrum of **1b** in DMSO-d_6_

*
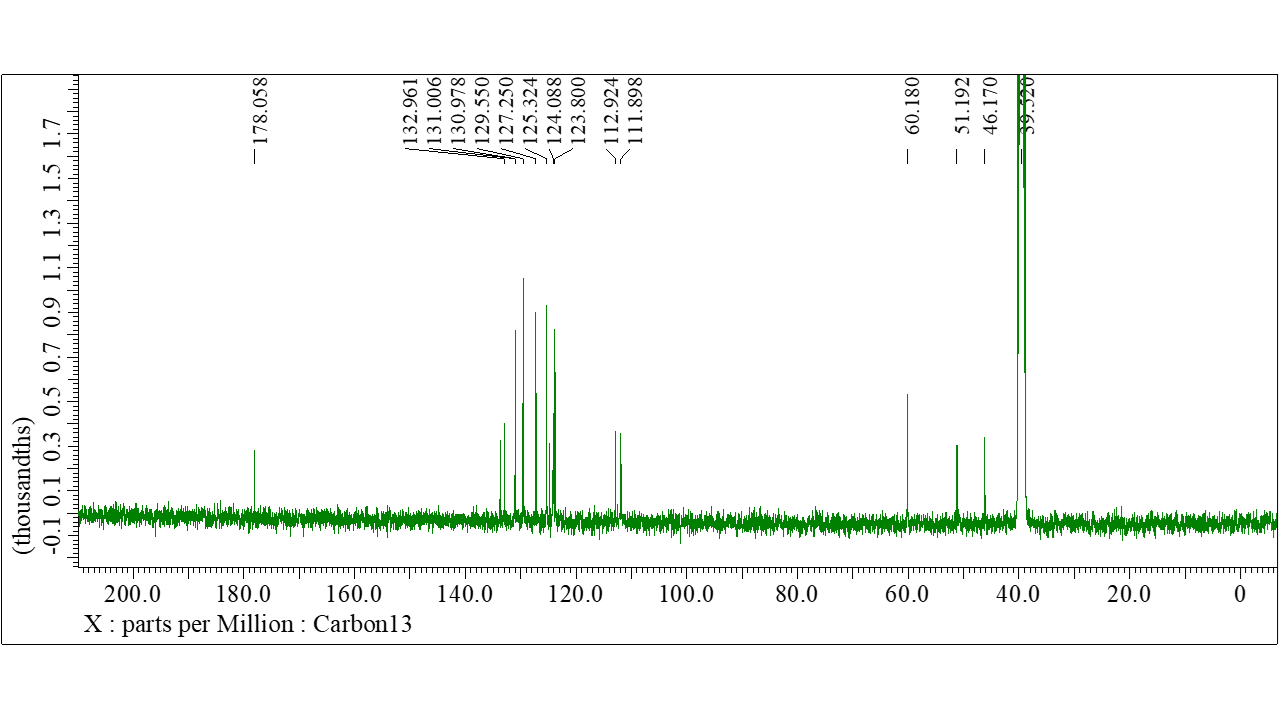
*

**Figure S13.** ^13^C-NMR spectrum of **1b** in DMSO-d_6_


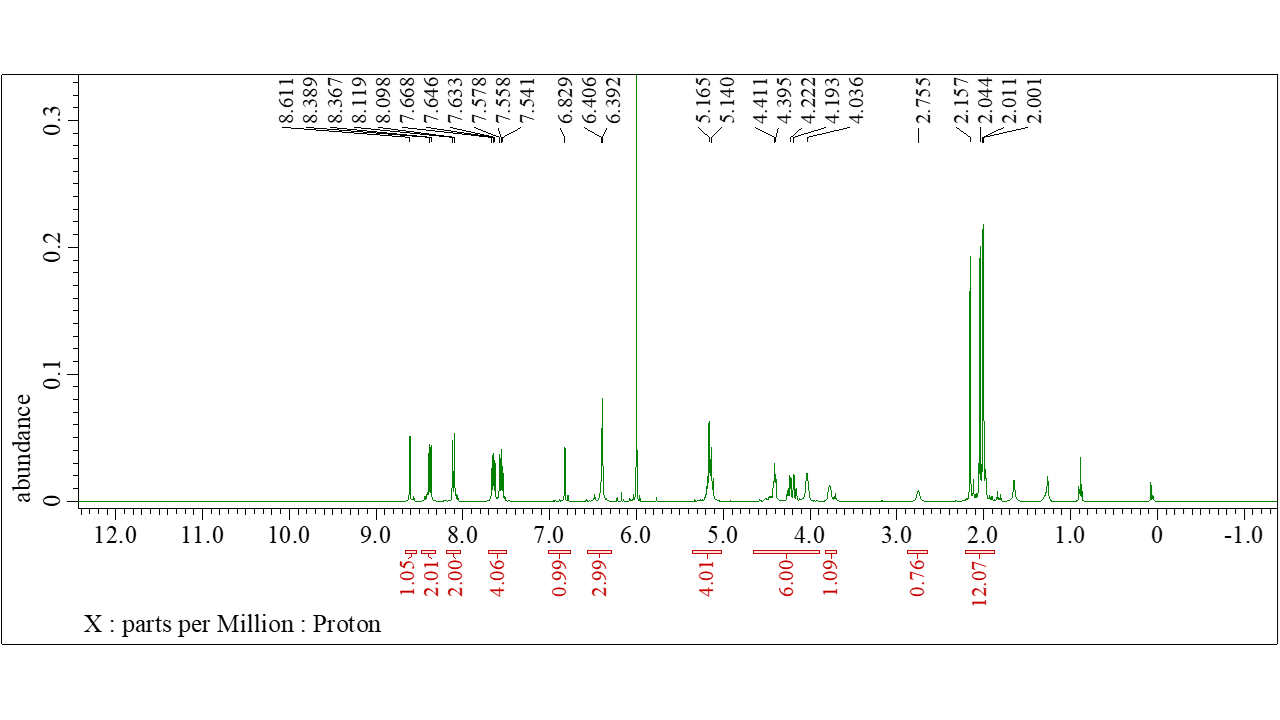


**Figure S14.** ^1^H-NMR spectrum of (1-(9-anthracenylmethyl)-3-(2-hydroxyethyl)imidazol-2-yliden)(3,4,5,6-tetra-O-acetyl-1-thio-beta-D-glucopyranose) gold complex **2a** in C_2_D_2_Cl_4_


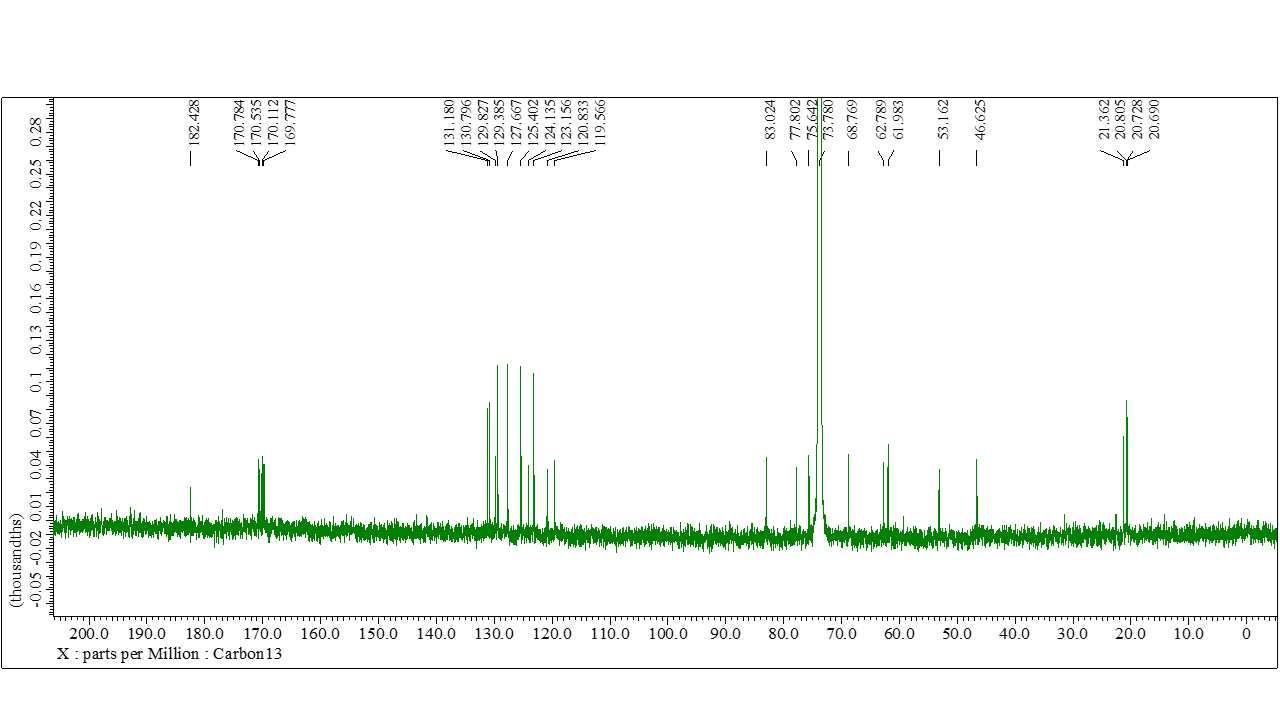


**Figure S15.** ^13^C-NMR spectrum of (1-(9-anthracenylmethyl)-3-(2-hydroxyethyl)imidazol-2-yliden)(3,4,5,6-tetra-*O*-acetyl-1-thio-beta-D-glucopyranose) gold complex **2a** in C_2_D_2_Cl_4_


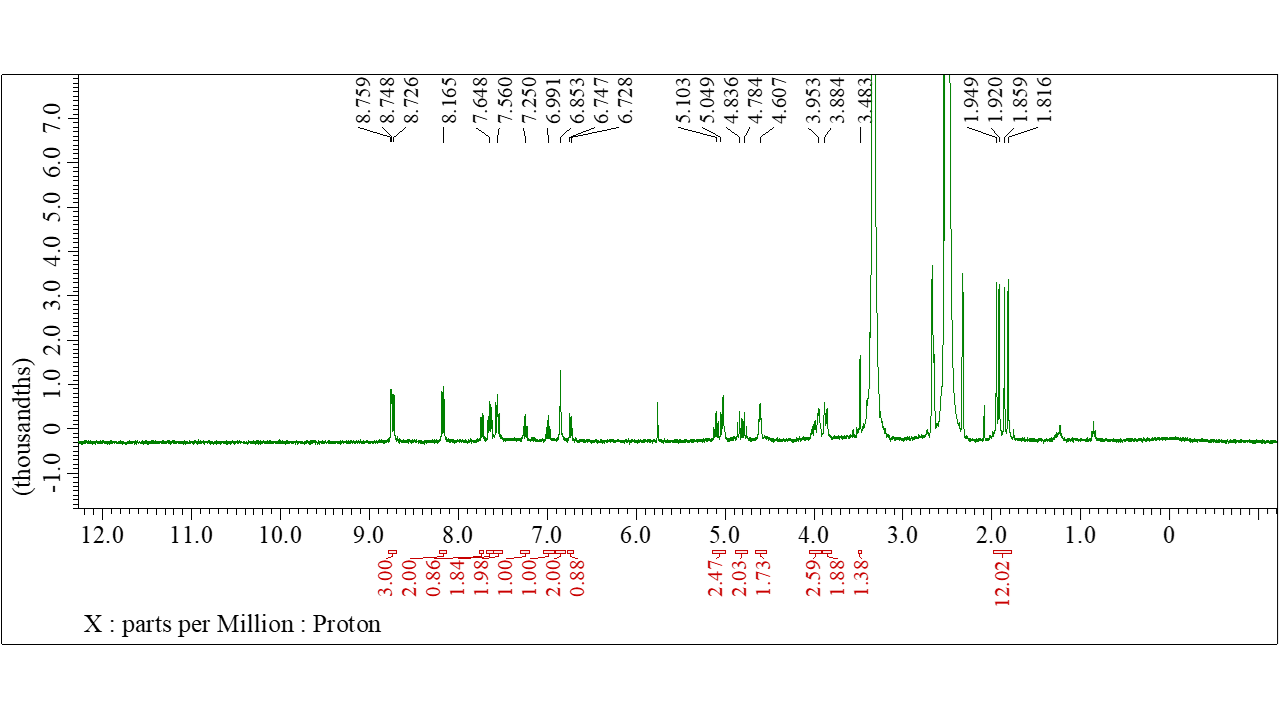


**Figure S16.** ^1^H-NMR spectrum of (1-(9-anthracenylmethyl)-3-(2-hydroxyethyl)benzimidazol-2-yliden)(3,4,5,6-tetra-*O*-acetyl-1-thio-beta-D-glucopyranose) gold complex **2b** in DMSO-d_6_


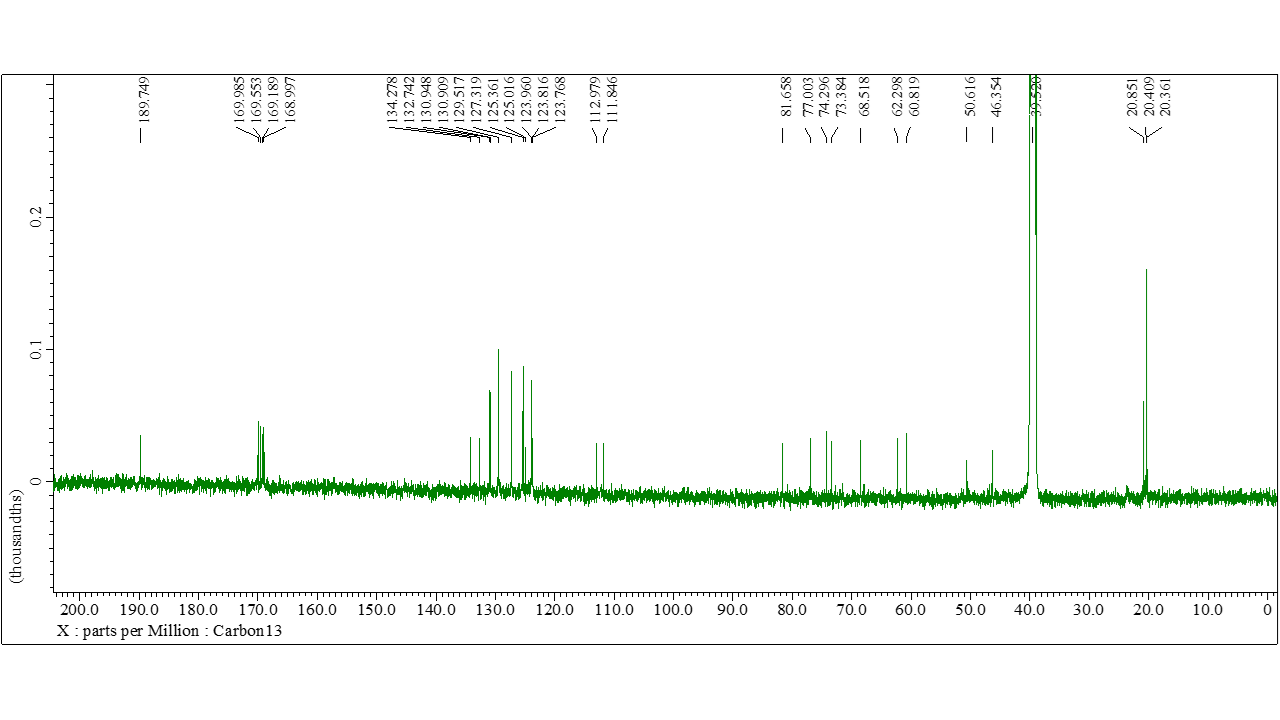


**Figure S17.** ^13^C-NMR spectrum of (1-(9-anthracenylmethyl)-3-(2-hydroxyethyl)benzimidazol-2-yliden)(3,4,5,6-tetra-O-acetyl-1-thio-beta-D-glucopyranose) gold complex **2b** in DMSO-d_6_

# LC-MS characterization spectra

**
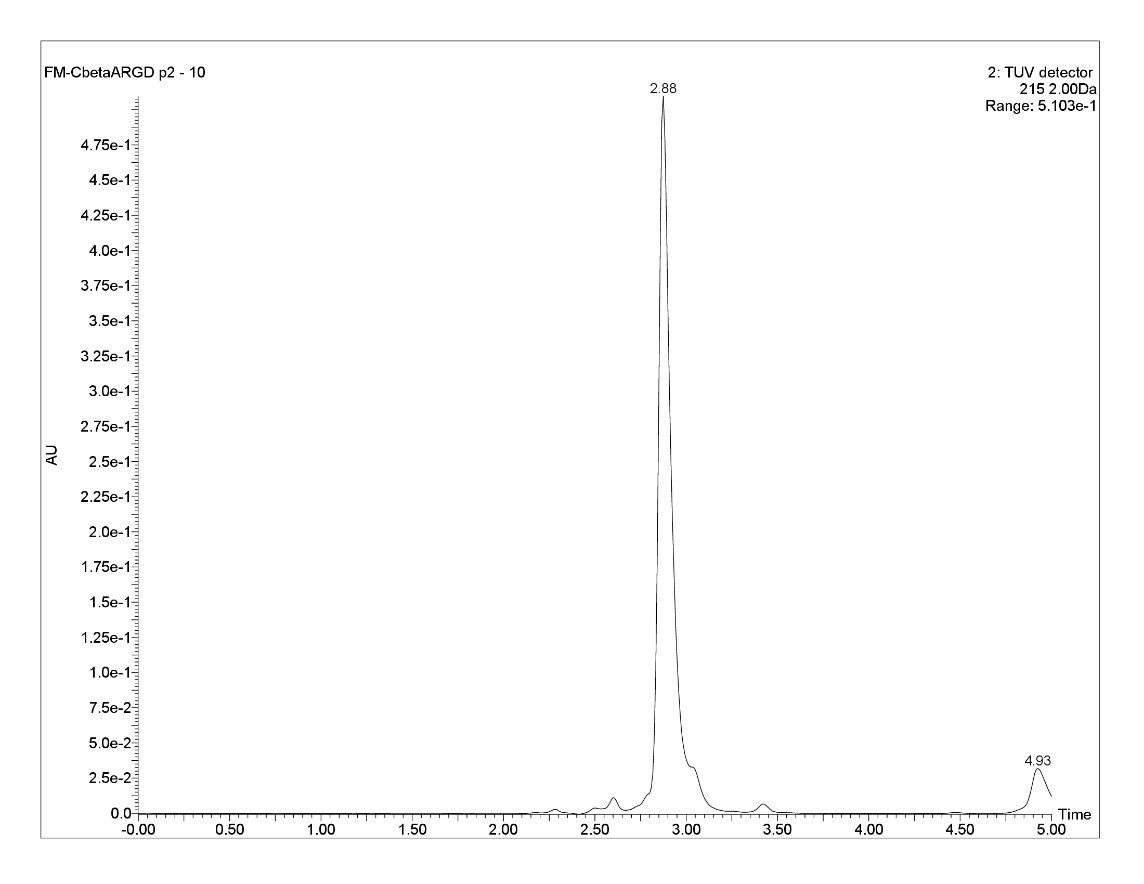
**

**
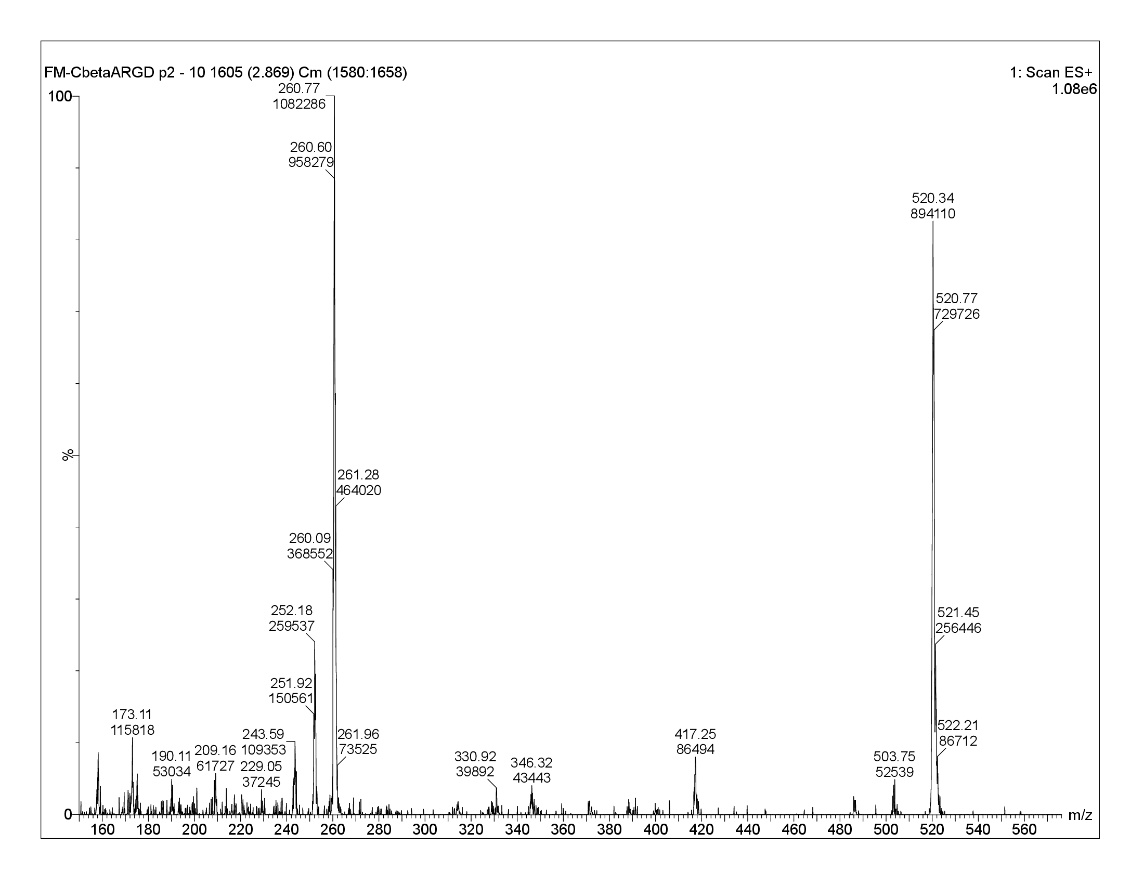
**

**Figure S18.** LC-MS characterization of peptide C-βAla-RGD (**P_1_**)

**
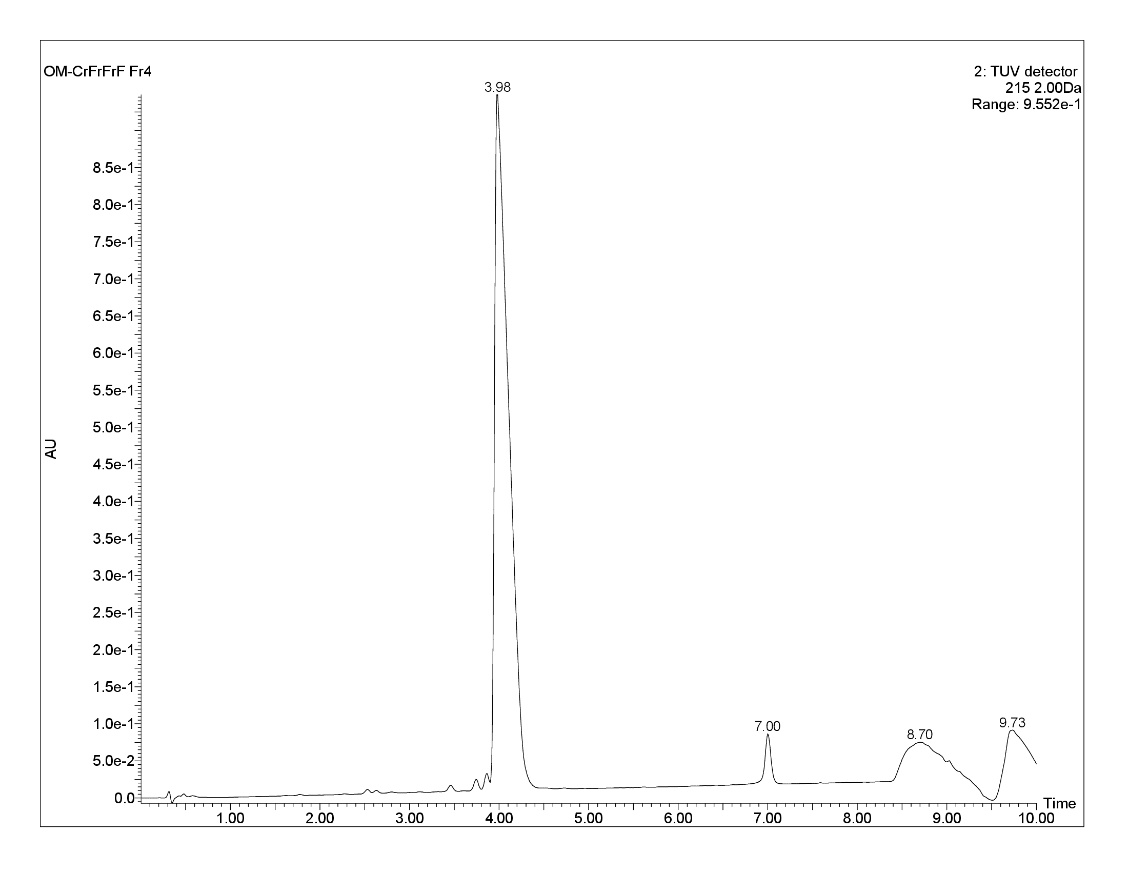
**

**
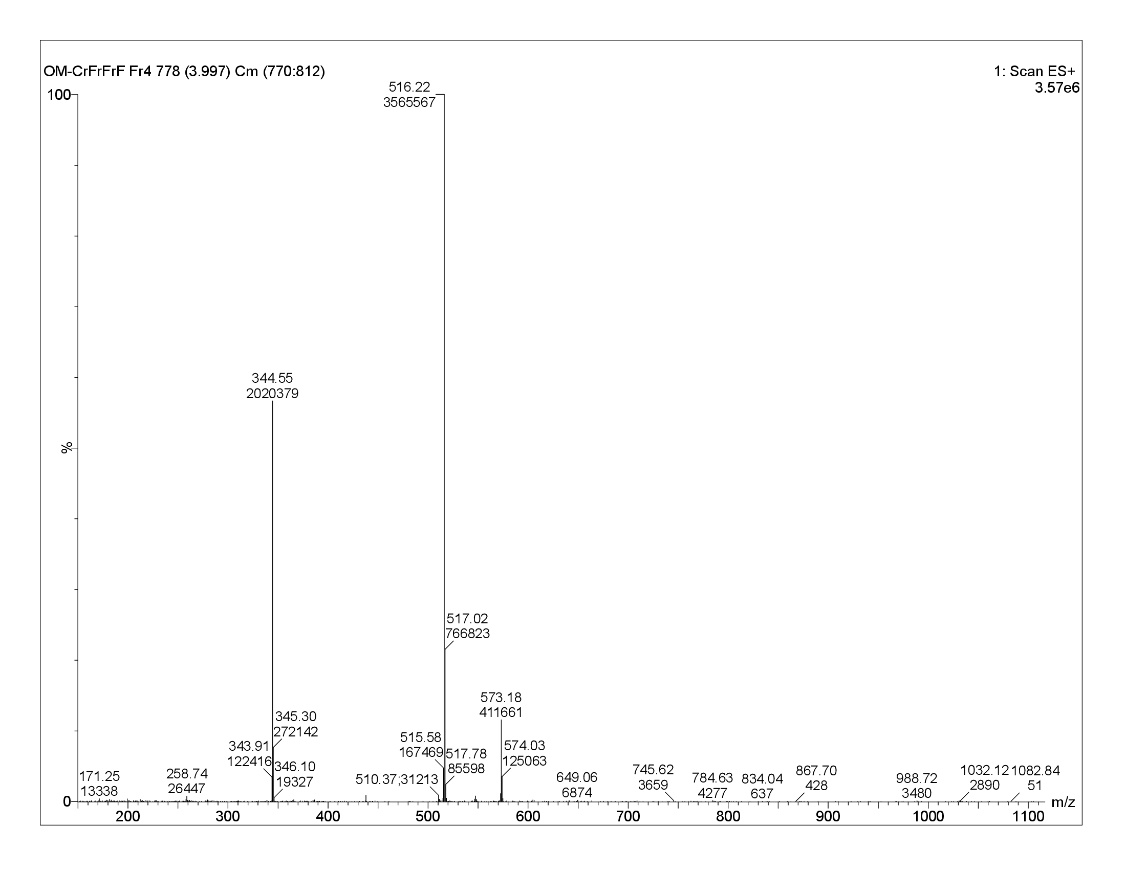
**

**Figure S19**. LC-MS characterization of peptide CrFrFrF (**P_2_**)

**
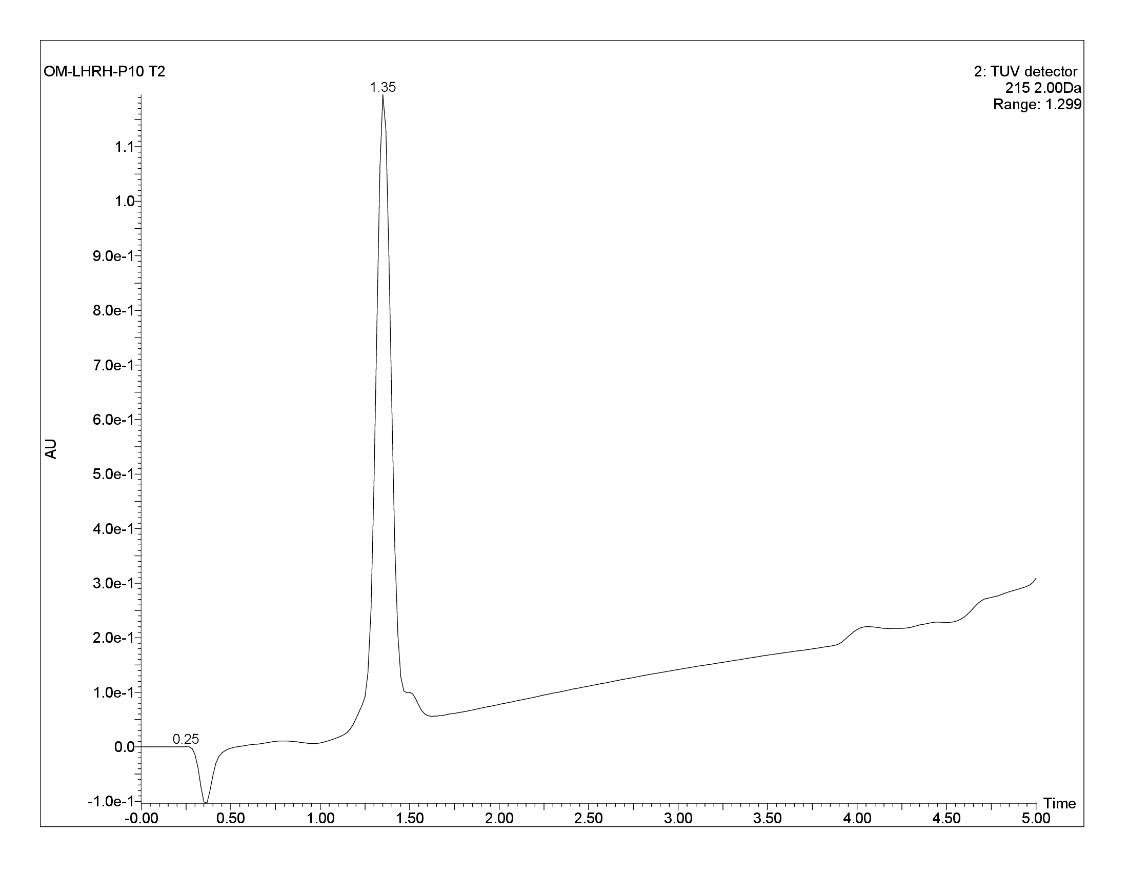
**

**
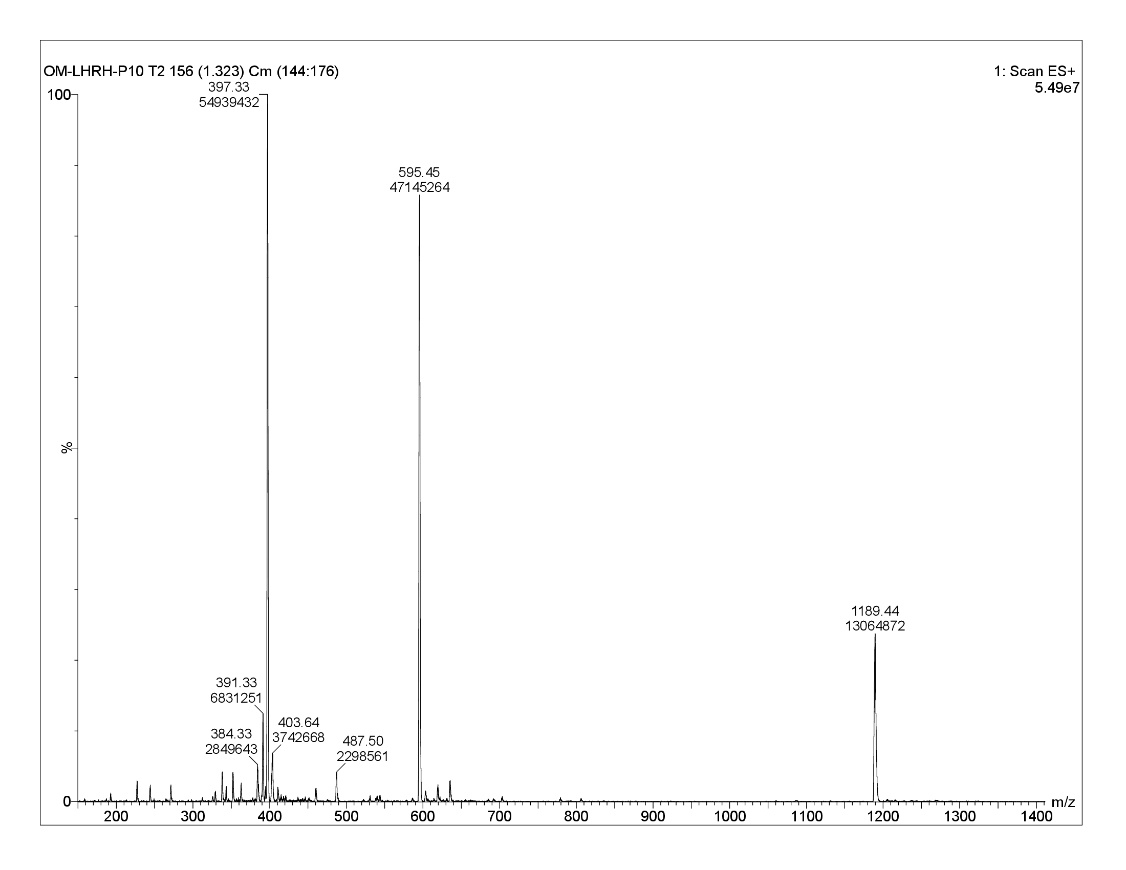
**

**Figure S20.** LC-MS characterization of peptide EHWSYCLRP (**P_3_**)

**Table S1.** MS characterization (*m/z*) for peptides P**_1_**-P**_3_**.

|  | **Calc [M+H]^+^** | **Calc [M+2H]^2+^** | **Calc [M+3H]^3+^** | **Found [M+H]^+^** | **Found [M+2H]^2+^** | **Found [M+3H]^3+^** |
| --- | --- | --- | --- | --- | --- | --- |
| **C-βAla-RGD** | 521.57 | 261.29 |  | 520.34 | 260.77 |  |
| **CrFrFrF** | 1032.26 | 516.63 | 344.76 |  | 516.22 | 344.55 |
| **EHWSYCLRP** | 1191.36 | 596.18 | 397.79 | 1189.44 | 595.45 | 397.33 |


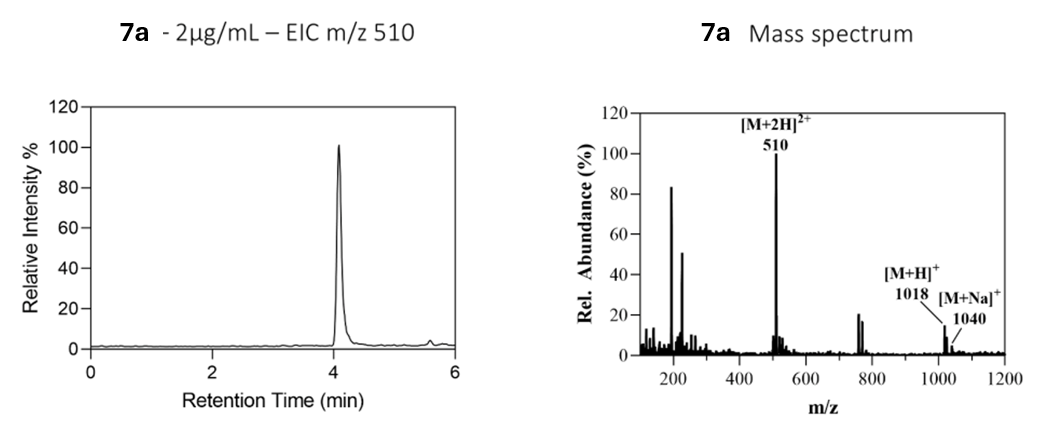


**Figure S21.** LC-MS characterization of bioconjugate **7a**


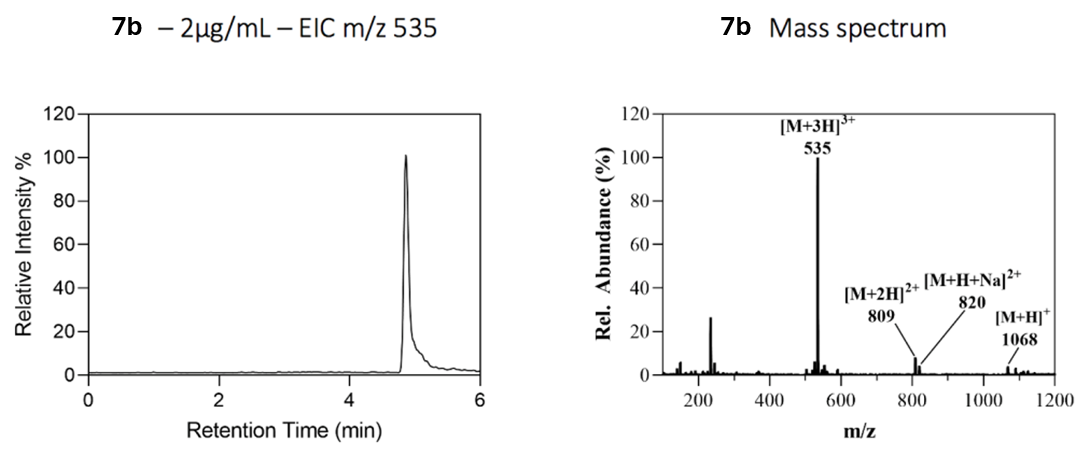


**Figure S22.** LC-MS characterization of bioconjugate **7b**


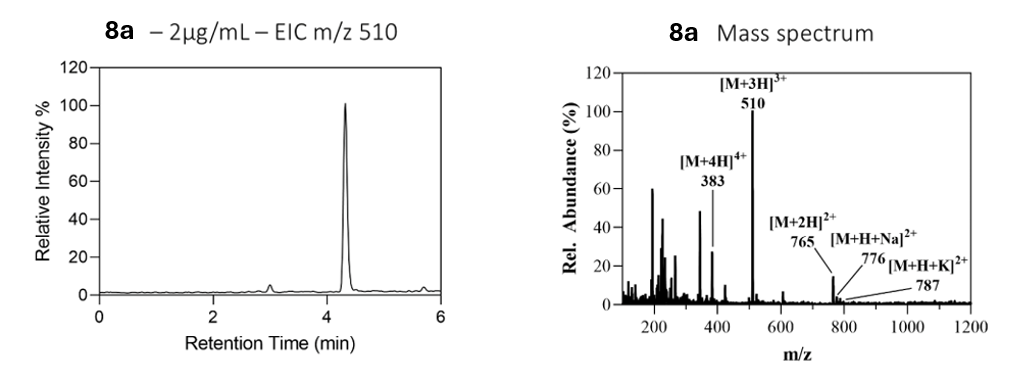


**Figure S23.** LC-MS characterization of bioconjugate **8a**


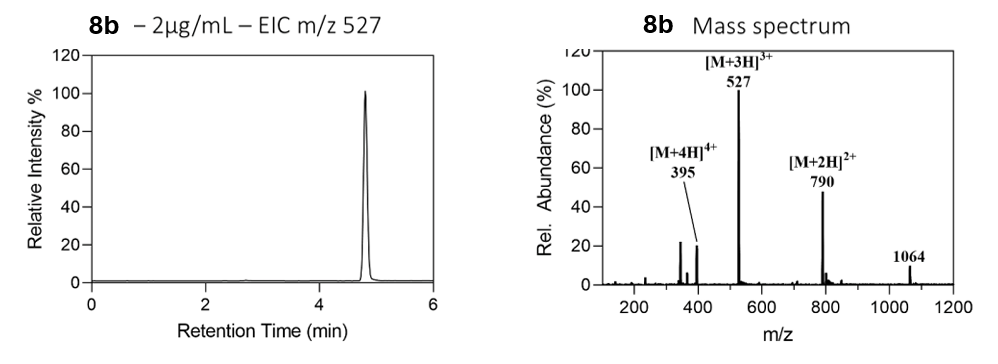


**Figure S24.** LC-MS characterization of bioconjugate **8b**


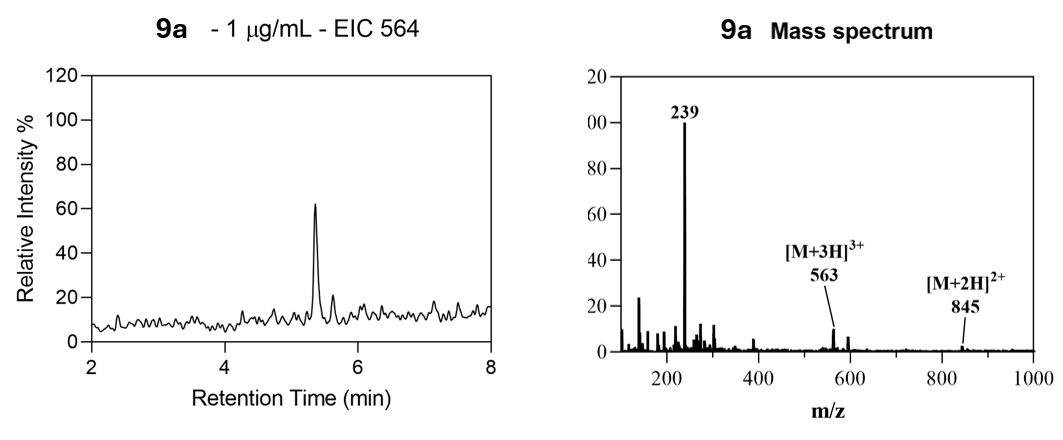


**Figure S25.** LC-MS characterization of bioconjugate **9a**


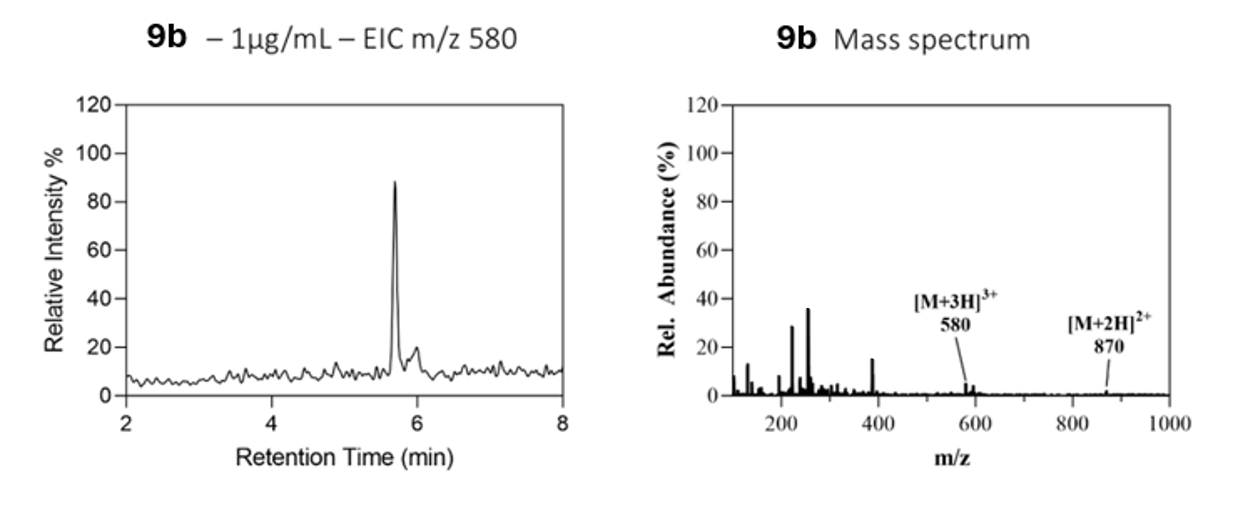


**Figure S26.** LC-MS characterization of bioconjugate **9b**

# Stability studies in DMSO/H_2_O


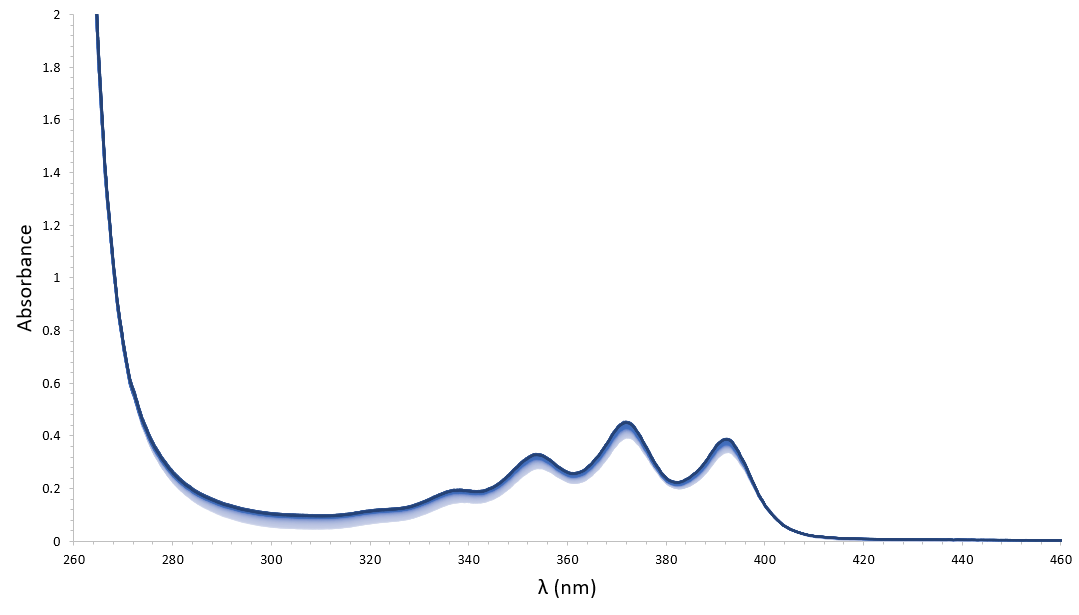


**Figure S27.** Spectra of **1a** in DMSO/H_2_O 1:1 ([**1a**]= 10^-5^M) recorded over 24 hours at 37 °C. The spectra were recorded every 30 min from 0 (**___**) to 24 (**___**) hours.


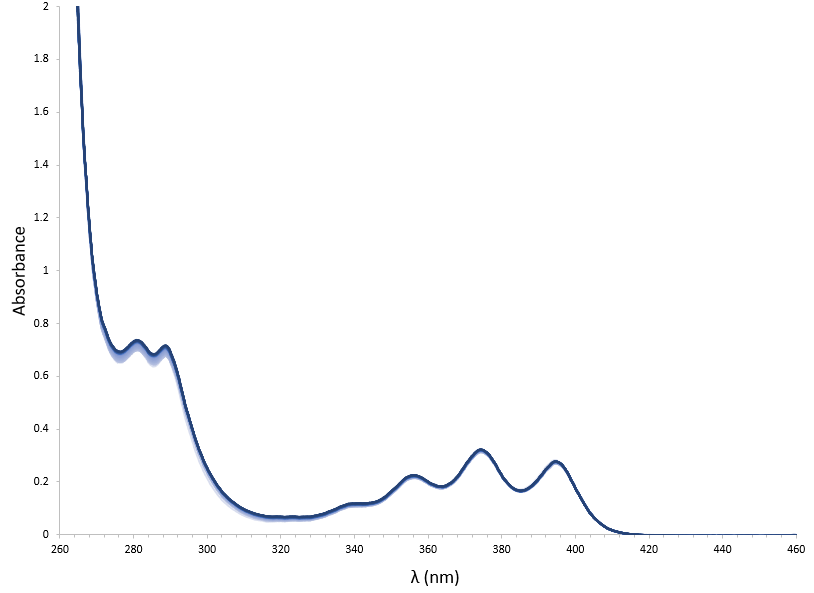


**Figure S28.** Spectra of **1b** in DMSO/H_2_O 1:1 ([**1b**]= 10^-5^M) recorded over 24 hours at 37 °C. The spectra were recorded every 30 min from 0 (**___**) to 24 (**___**) hours.


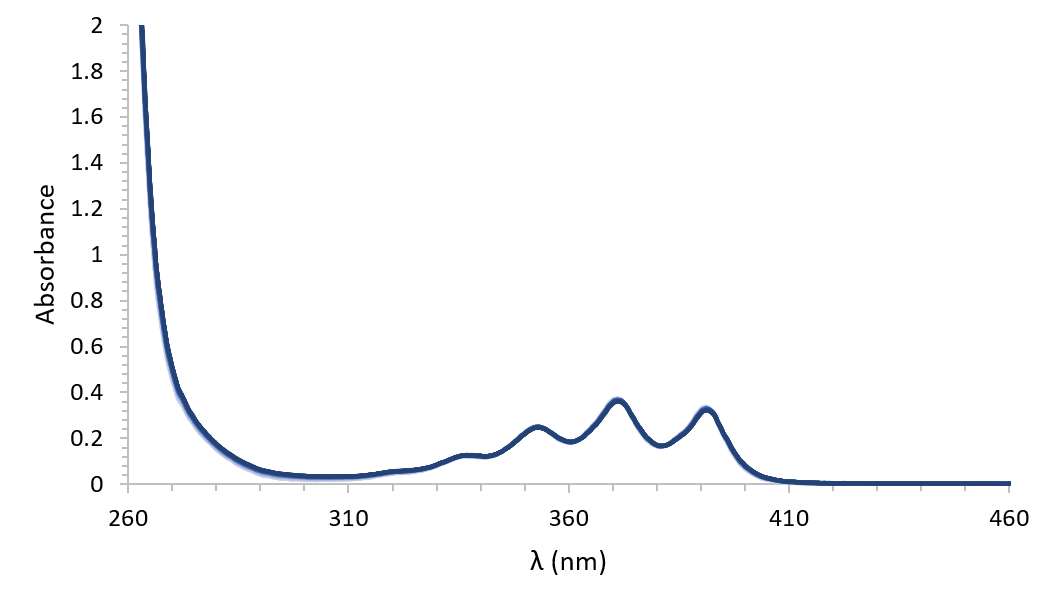


**Figure S29.** Spectra of **2a** in DMSO/H_2_O 1:1 ([**2a**]= 10^-5^M) recorded over 24 hours at 37 °C. The spectra were recorded every 30 min from 0 (**___**) to 24 (**___**) hours.


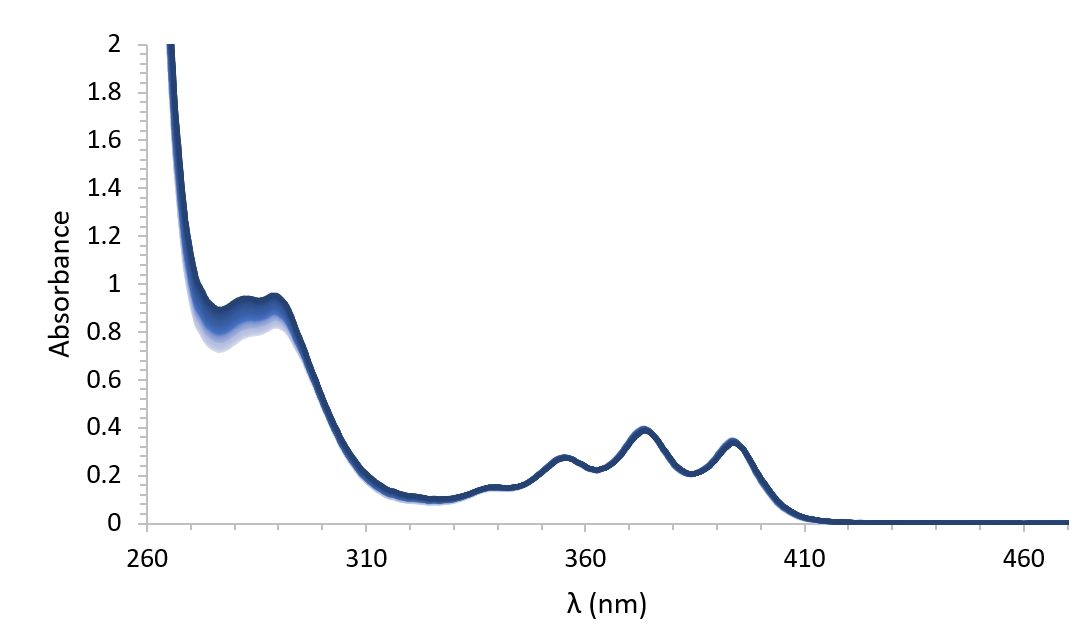


**Figure S30.** Spectra of **2b** in DMSO/H_2_O 1:1 ([**2b**]= 10^-5^M) recorded over 24 hours at 37 °C. The spectra were recorded every 30 min from 0 (**___**) to 24 (**___**) hours.

# Fluorescence interaction studies with HSA


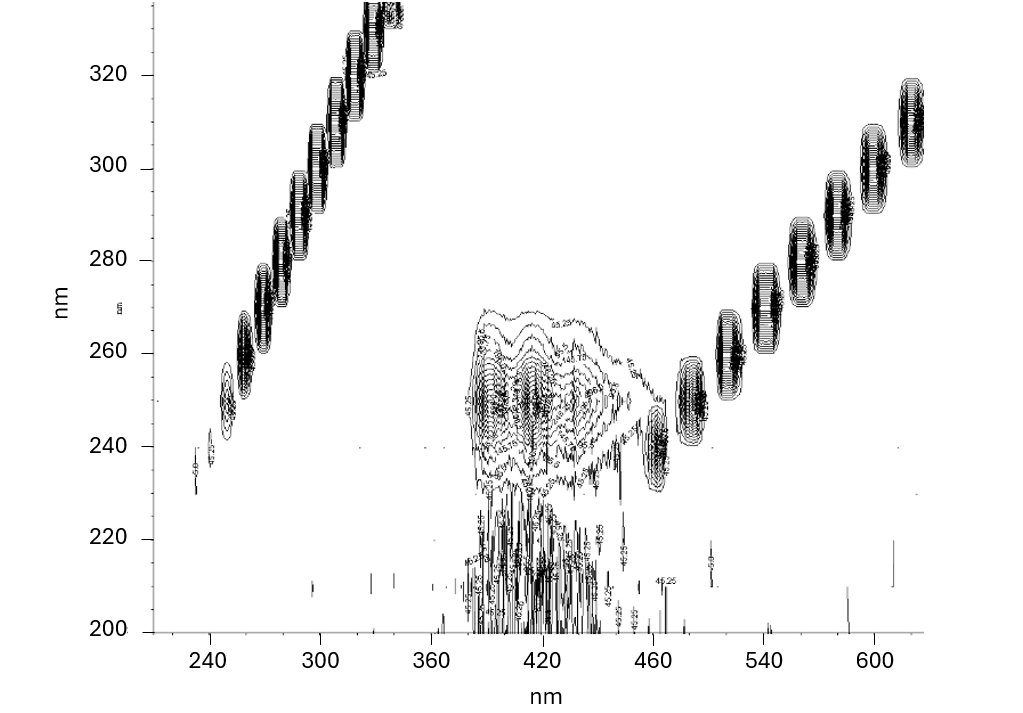


**Figure S31.** 3D Fluorescence spectra of complex **2a** at different excitation wavelengths (x-axis= emission wavelength; y-axis= excitation wavelength; z-axis = intensity.


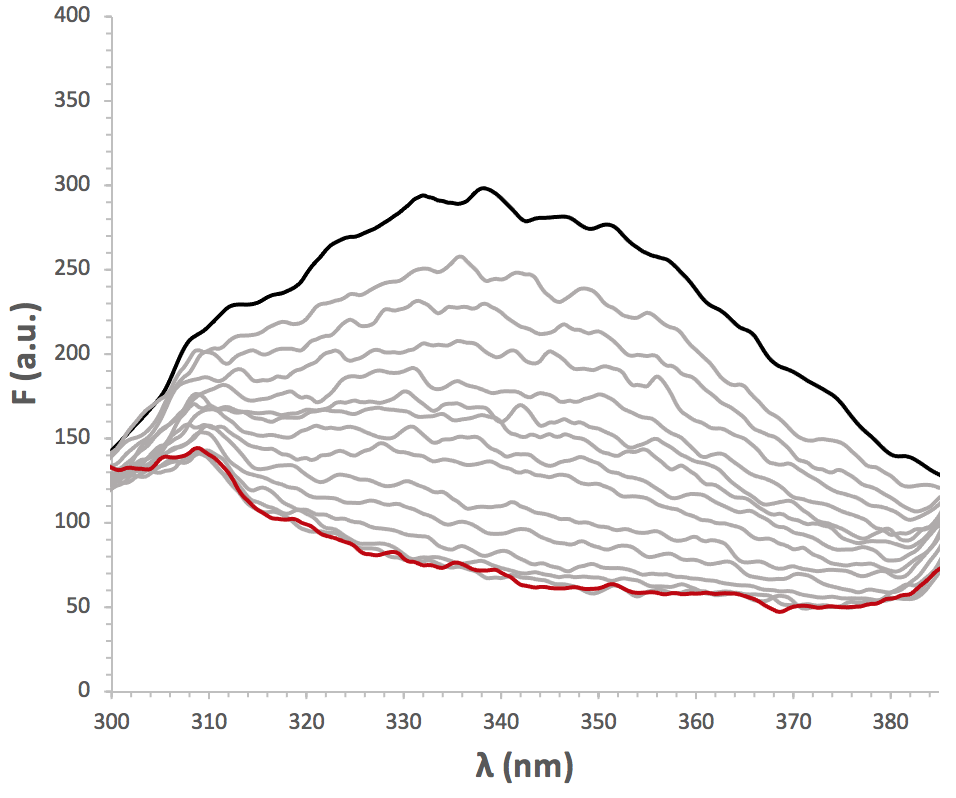


**Figure S32**. Fluorescence spectra, corrected for dilution and inner filter effect, of HSA in the presence of increasing amounts of complex **1b**. C_HSA_ 10^-6^ M, C**_1b_** from 0 M (**___**) to 1.12 x 10^-5^ M (**___**); aqueous buffer NaCac 2.5 mM, pH = 7.0, T = 37.0 °C; slits exc/em 3 nm/3nm.





**Figure S33.** Graphs of Equation 1 for **1a** /HSA at 37 °C (black circles) and **1b**/HSA at 50 °C (blue squares) systems; C_HSA_ = 10^-6^ M; NaCac 2.5 mM, pH = 7.0, λ_em_= 330 nm, λ_ex_= 280 nm.

# Mass spectrometry interaction studies with HSA


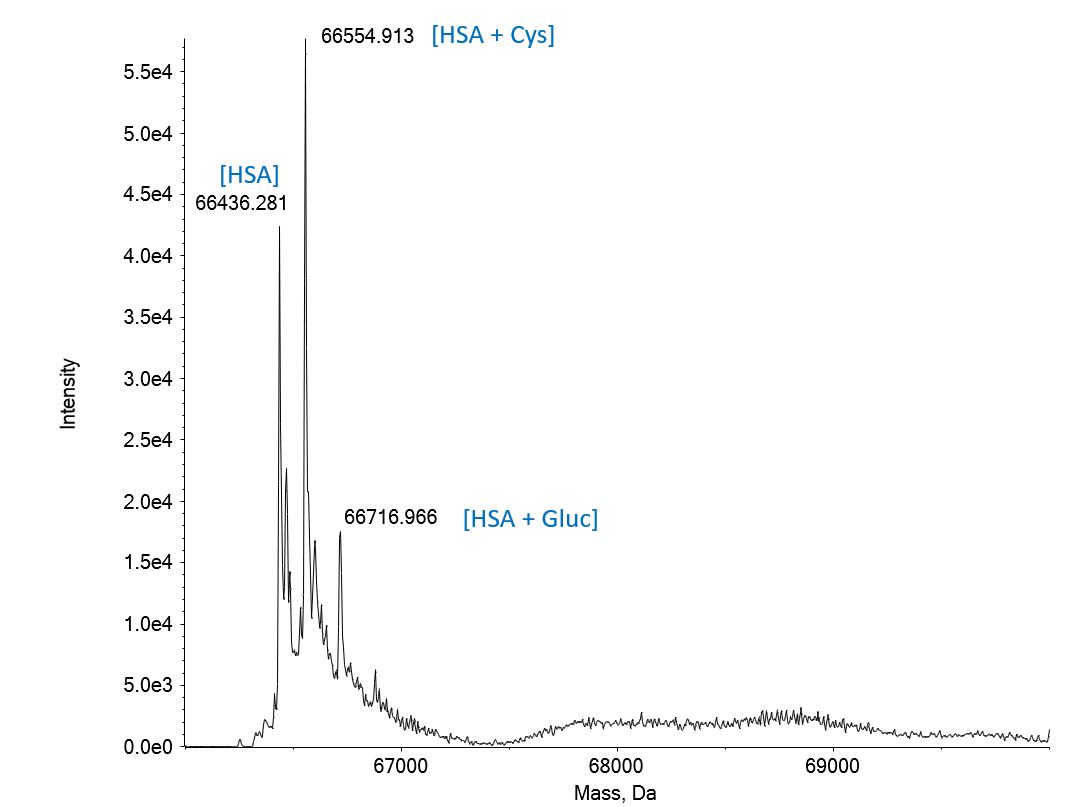


**Figure S34.** Deconvoluted ESI-Q-TOF mass spectrum of HSA solution 10^-4^ M in 20 mM ammonium acetate solution, pH 6.8.


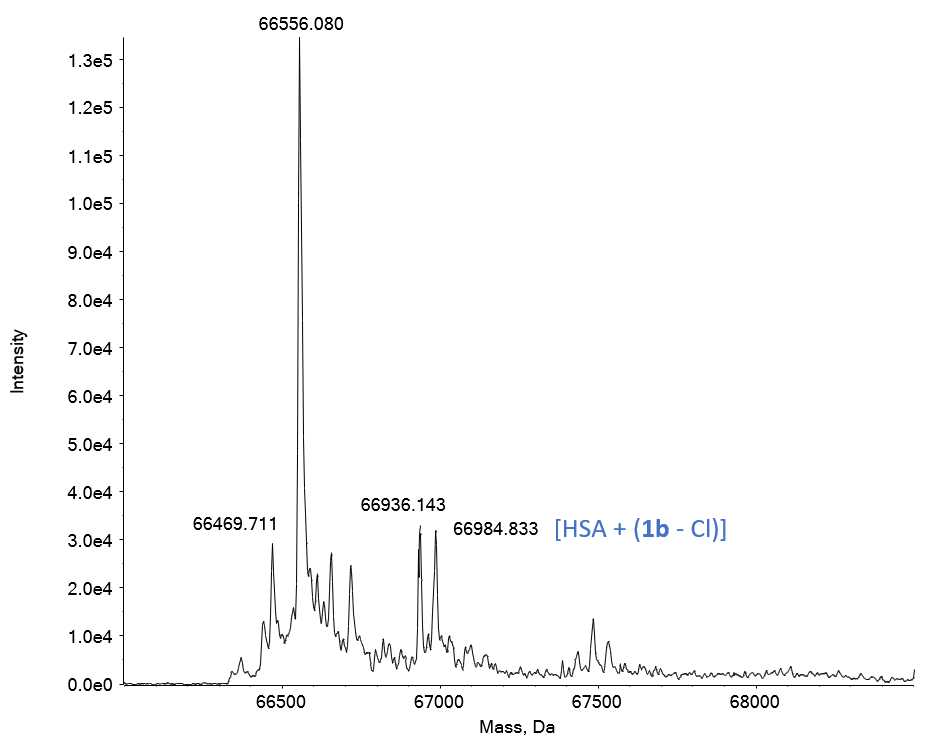


**Figure S35.** Deconvoluted ESI-Q-TOF mass spectrum of HSA solution 10^-4^ M incubated for 4 h at 37 °C with **1b** (2:1 metal to protein ratio) in 20 mM ammonium acetate solution, pH 6.8.


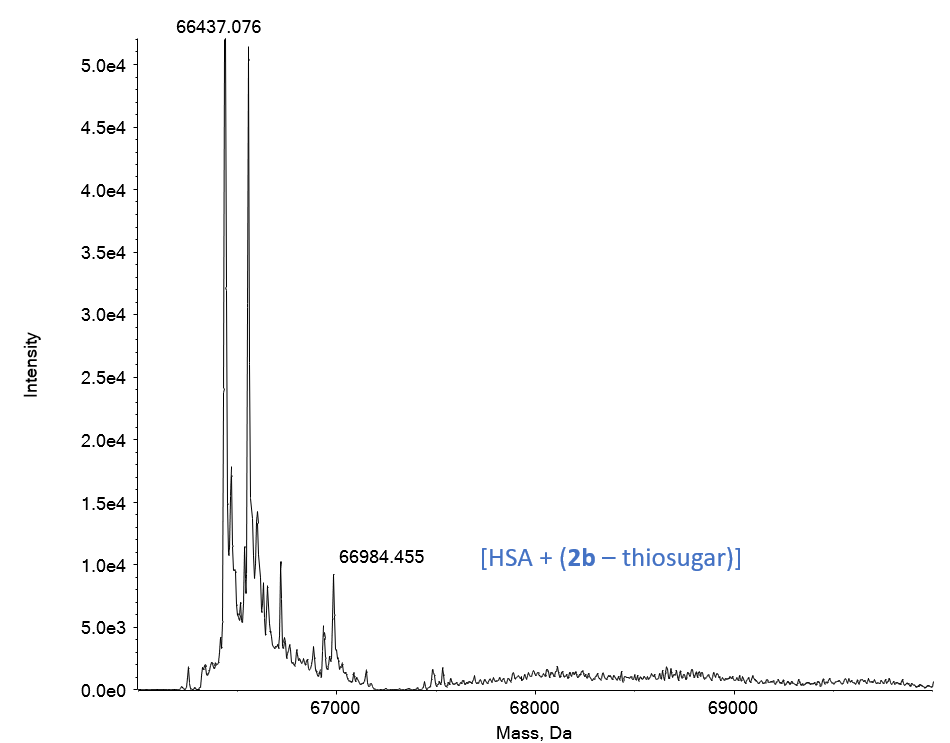


**Figure S36.** Deconvoluted ESI-Q-TOF mass spectrum of HSA solution 10^-4^ M incubated for 4 h at 37 °C with **2b** (2:1 metal to protein ratio) in 20 mM ammonium acetate solution, pH 6.8.

# Cytotoxicity curves


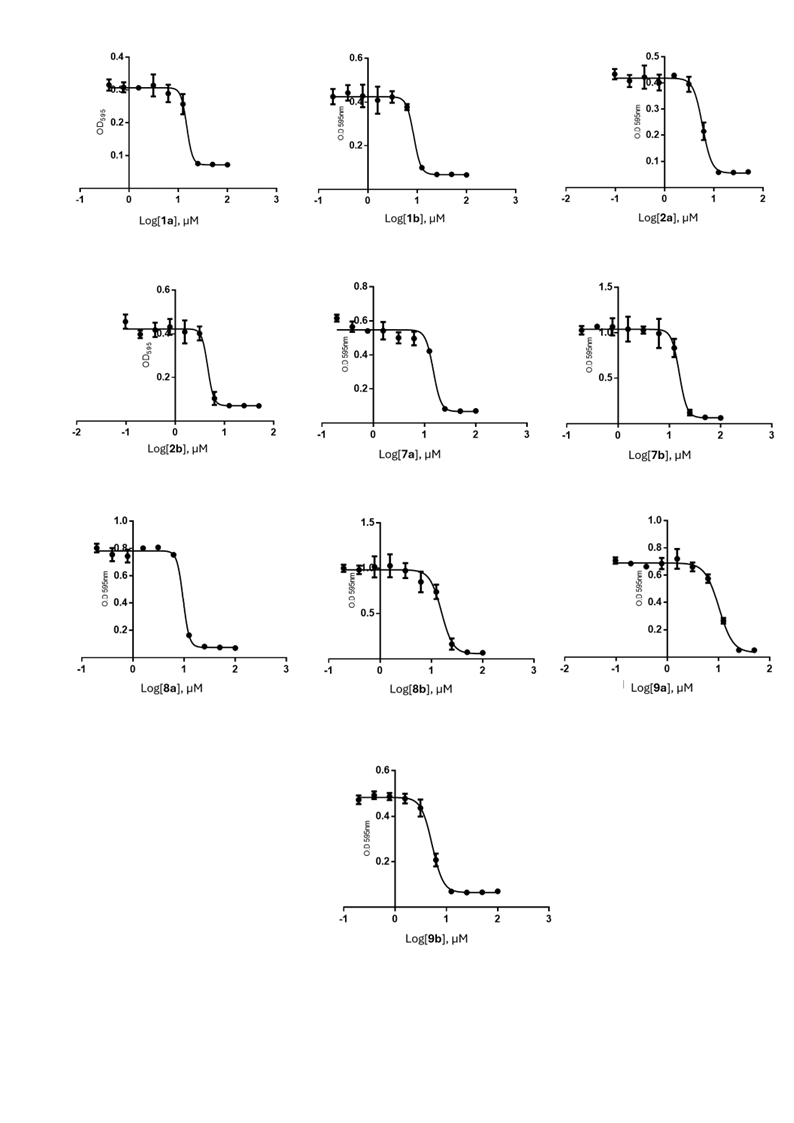


**Figure S37.** Dose-response curve (by MTT assay) after 72 h exposure of complexes **1a-b**, **2a-b**, **7a-b**, **8a-b**, **9a-b** in A2780/S (ovarian adenocarcinoma) cancer cell line. The curves are the results of three biological independent experiments. The statistical analysis was performed using GraphPad Prism 6.0. The obtained IC50 values (µM) were also reported in Tables 2 and 3.

**
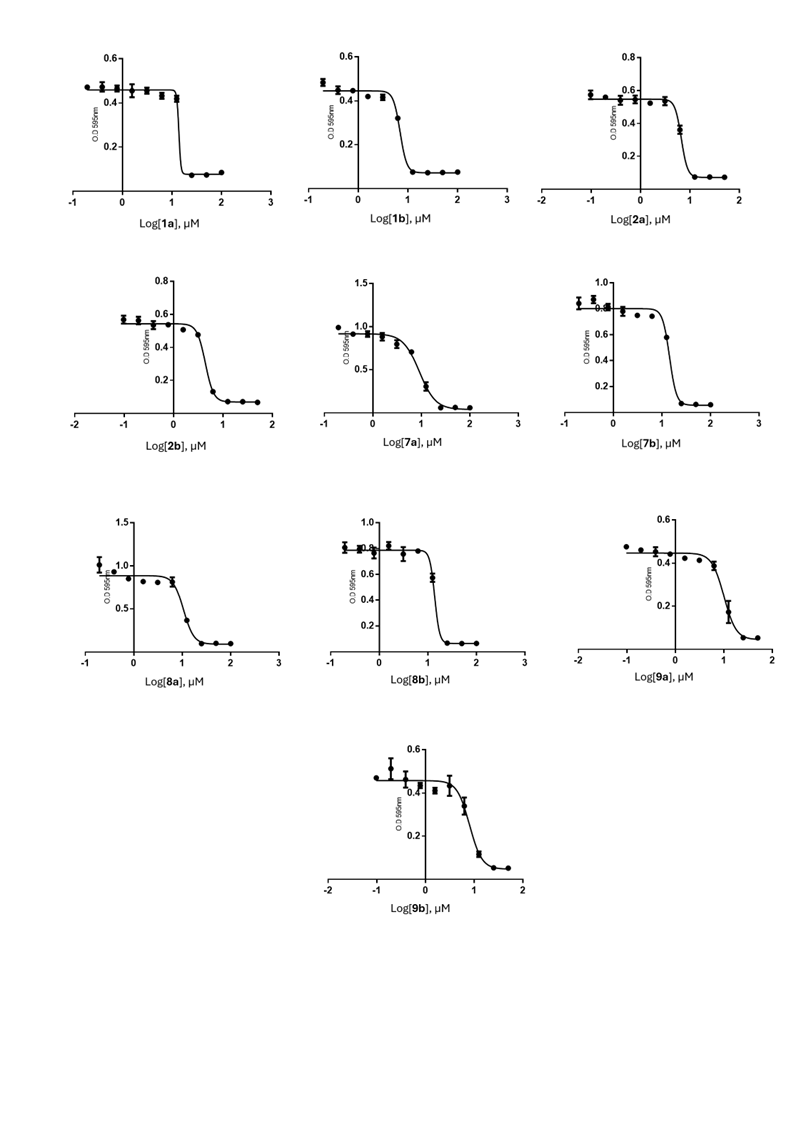
**

**Figure S38.** Dose-response curve (by MTT assay) after 72 h exposure of complexes **1a-b**, **2a-b**, **7a-b**, **8a-b**, **9a-b** in A2780/R (ovarian adenocarcinoma cisplatin resistant) cancer cell line. The curves are the results of three biological independent experiments. The statistical analysis was performed using GraphPad Prism 6.0. The obtained IC50 values (µM) were also reported in Tables 2 and 3.

**
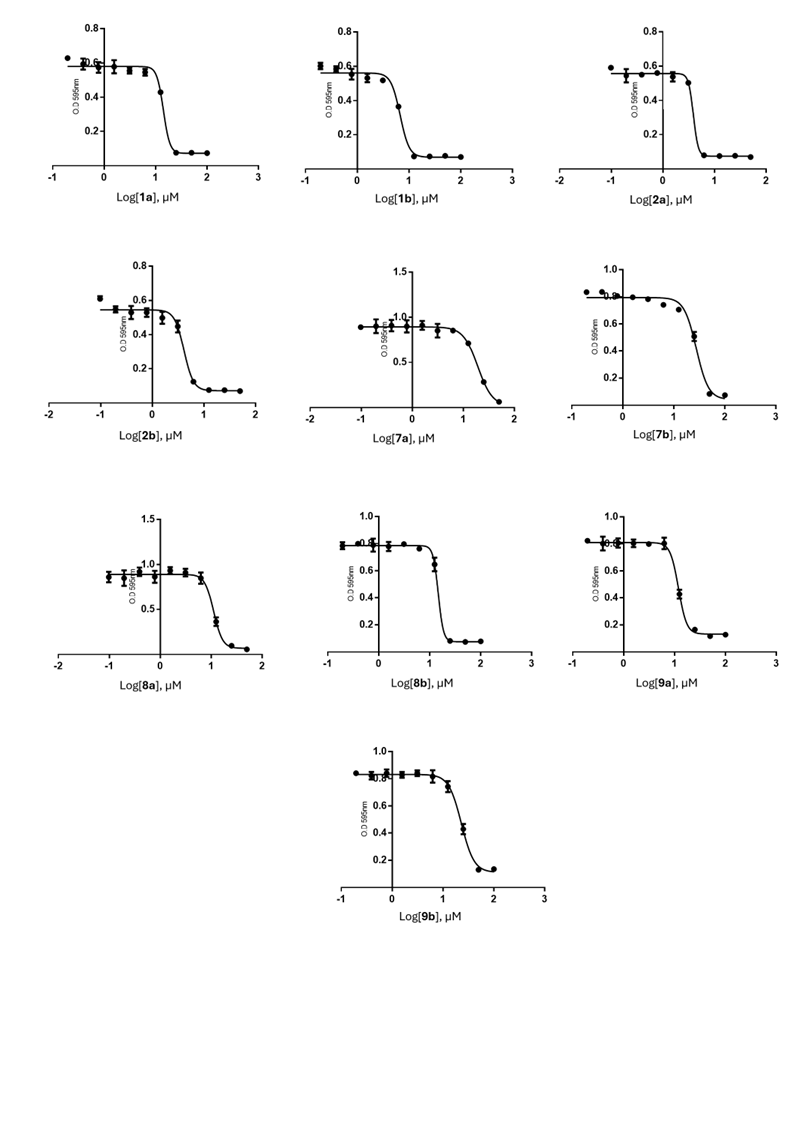
**

**Figure S39.** Dose-response curve (by MTT assay) after 72 h exposure of complexes **1a-b**, **2a-b**, **7a-b**, **8a-b**, **9a-b** in SKOV-3 (ovarian cystadenocarcinoma) cancer cell line. The curves are the results of three biological independent experiments. The statistical analysis was performed using GraphPad Prism 6.0. The obtained IC50 values (µM) were also reported in Tables 2 and 3.

**
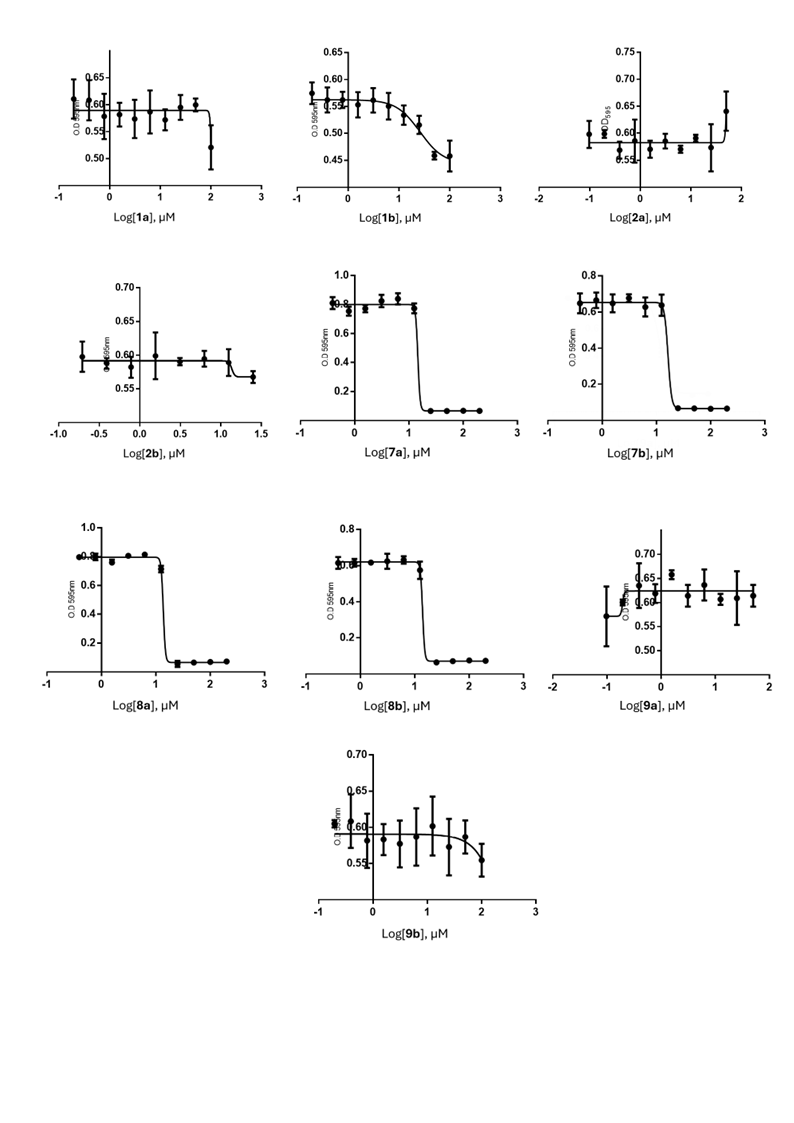
**

**Figure S40.** Dose-response curve (by MTT assay) after 72 h exposure of complexes **1a-b**, **2a-b**, **7a-b**, **8a-b**, **9a-b** in HSkMC (human skeletal muscle cells) healthy cell line. The curves are the results of three biological independent experiments. The statistical analysis was performed using GraphPad Prism 6.0. The obtained IC50 values (µM) were also reported in Tables 2 and 3.

# Confocal microscopy studies


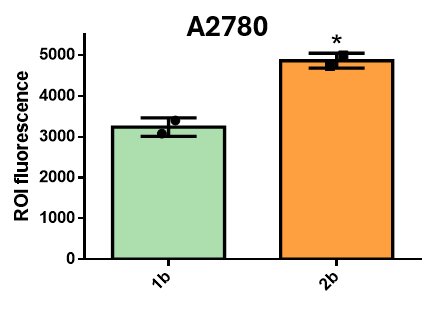

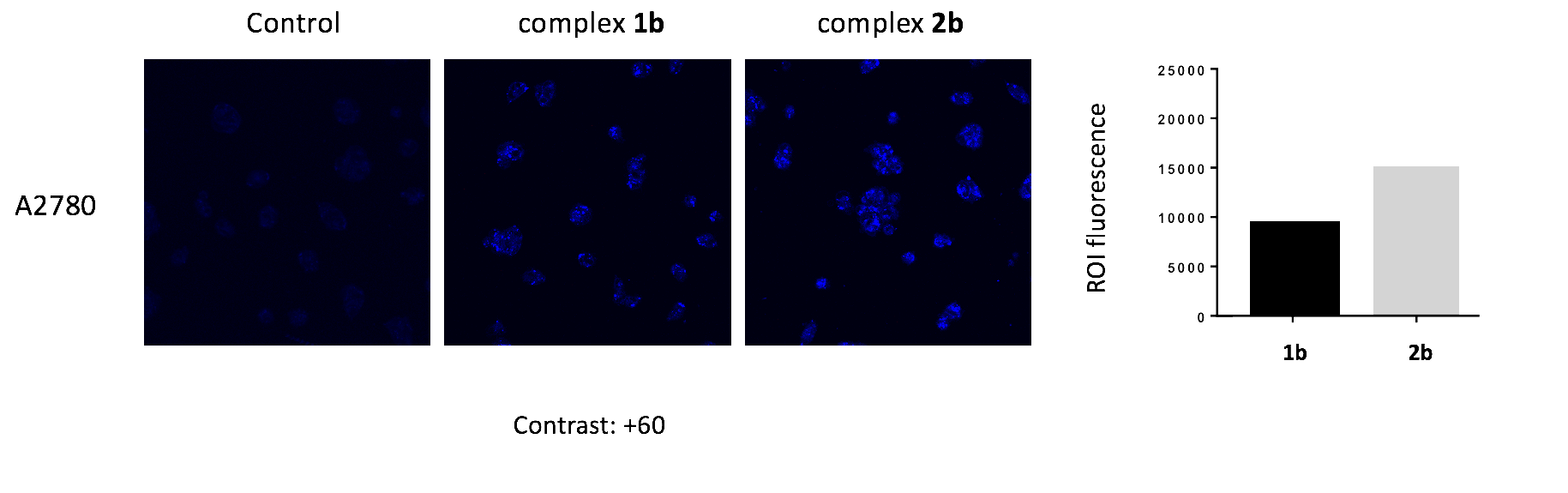


**Figure S41.** Fluorescence images, obtained through confocal microscopy, of A2780/S cells treated with complexes **1b** and **2b**, at a concentration 10 µM for 15 minutes.

**Figure S42**. A comparison between the fluorescence percentage emitted by the A2780/S cells treated with complexes **1a**, **2a**, **1b**, **2b**.

# Fluorescence-Activated Cell Sorting (FACS) studies


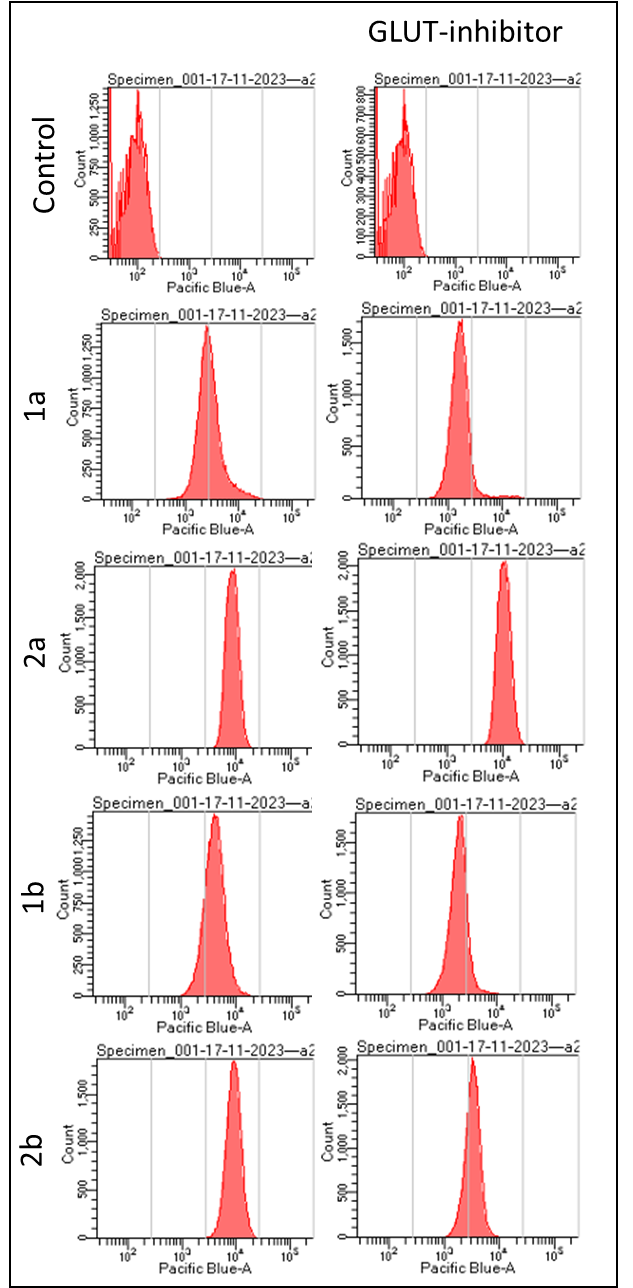

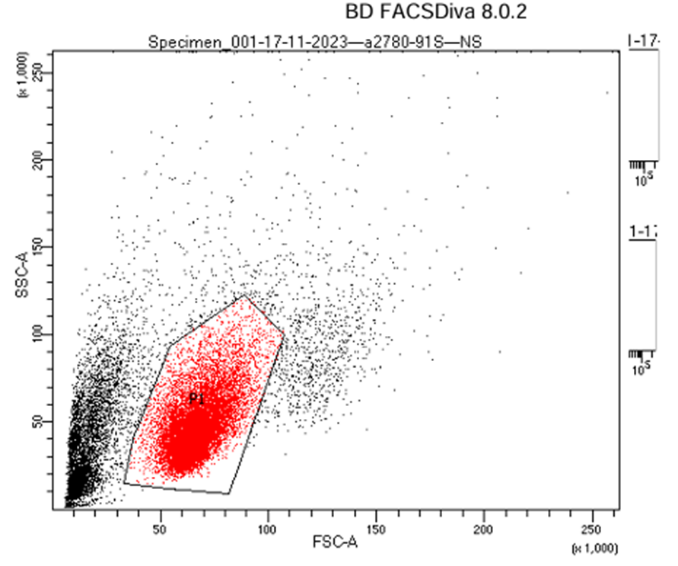


**Figure S43**. Representative FACS scatter dot plot (on the left) obtained by interpolating the Side Scatter (SSC-A) and Forward Scatter (FSC-A) values of A2780/S cells. SSC-A measures side-scattered light, which is proportional to the internal granularity of the cell. FSC-A measures forward scattered light, proportional to cell size. The distribution of cells according to these two properties allowed selection by gating of the P1 cell population (red) for analysis. Each point on the graph represents a single cell. In the right panel, representative fluorescence emission peaks in the Pacific Blue channel (421 nm) of A2780/S cells treated with complexes **1a**, **2a**, **1b**, and **2b** at a concentration of 10 µM for 10 min in the absence or presence of the GLUT-1 inhibitor.

# Experimental parameters for peptide synthesis and purification

**Table S2.** Instrumental parameters used for the MW-SPPS

| Step | Temperature (°C) | Power (W) | Time (s) |
| --- | --- | --- | --- |
| Deprotection | 75 | 175 | 15 |
|  | 90 | 30 | 50 |
| Standard Coupling | 75 | 175 | 15 |
|  | 90 | 30 | 110 |

**Table S3.** Instrumental parameters used for the MW-SPPS His

| Step | Temperature (°C) | Power (W) | Time (s) |
| --- | --- | --- | --- |
| Deprotection | 25 | 0 | 600 |
| Coupling | 25 | 0 | 120 |
|  | 50 | 50 | 480 |

**Table S4.** Instrumental parameters used for peptide purification. CV=column volumes

| Peptides | Flash method | ESI-MS  [M+H]^+^(*m/z*)  Found (calc) | ULPC gradient (Rt, min) |
| --- | --- | --- | --- |
| **P1** | 0-50 % B in 8 CV | 520.3 (520.5) | Isocratic 1% B in 5 min (2.85 |
| **P2** | 0 -50 % B in 7 CV | 1031.6 (1031.2) | 5-20% B in 10 min (3.92) |
| **P3** | 10-50 % B in 15 CV | 1189.6 (1189.5) | 10-90%B in 5 min (1.49) |
